# Me_3_Al-mediated domino nucleophilic addition/intramolecular cyclisation of 2-(2-oxo-2-phenylethyl)benzonitriles with amines; a convenient approach for the synthesis of substituted 1-aminoisoquinolines

**DOI:** 10.3762/bjoc.17.186

**Published:** 2021-11-16

**Authors:** Krishna M S Adusumalli, Lakshmi N S Konidena, Hima B Gandham, Krishnaiah Kumari, Krishna R Valluru, Satya K R Nidasanametla, Venkateswara R Battula, Hari K Namballa

**Affiliations:** 1 GVK Biosciences Private Limited, Medicinal Chemistry Laboratory, Hyderabad 500076, India; 2 Department of Engineering Chemistry, Andhra University College of Engineering (A), Andhra University, Visakhapatnam 530003, India; 3 Department of Chemistry, Hunter College, City University of New York, 695 Park Avenue, NY 10065, USA

**Keywords:** 1-aminoisoquinolines, CWJ-a-5, intramolecular cyclisation, 2-(2-oxo-2-phenylethyl)benzonitriles, nucleophilic addition

## Abstract

A simple and efficient protocol for the construction of 1-aminoisoquinolines was achieved by treating 2-(2-oxo-2-phenylethyl)benzonitriles with amines in the presence of Me_3_Al. The reaction proceeds via a domino nucleophilic addition with subsequent intramolecular cyclisation. This method provides a wide variety of substituted 1-aminoisoquinolines with good functional group tolerance. Furthermore, the synthetic utility of this protocol was demonstrated in the successful synthesis of the antitumor agent CWJ-a-5 in gram scale.

## Introduction

**Figure 1:**
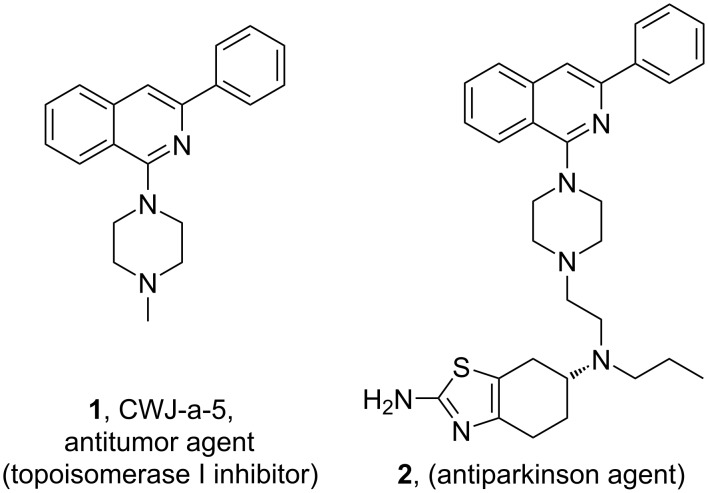
Biologically active 1-aminoisoquinolines.

Heterocyclic compounds have always been recognized as the frameworks of interest in organic and medicinal fields. Particularly, aza-heteroarenes have attracted burgeoning interest in the research community owing to their structural and biological significance [1–4]. The isoquinoline template represents a huge family of aza-heterocycles with unparalleled structural diversity, and is considered to be associated with a huge range of applications in medicinal and materials sciences [5–12]. 1-Amino substituted isoquinoline derivatives are extensively studied owing to their therapeutic applications in medicinal chemistry such as antimalarial, anti-Parkinson and antitumor activity (Figure 1) [13–17]. They also display remarkable enzymatic inhibitory activities on topoisomerase I, [18] mutant B-Raf [19] and exhibit antagonistic activities towards adenosine A3 [20] and PDE4B [21] receptors. They are useful in the synthesis of phosphorescent materials [22–24], fluorosensors [25]. and also found as chiral ligands in a variety of transition metal catalysts [26–30].

**Scheme 1:**
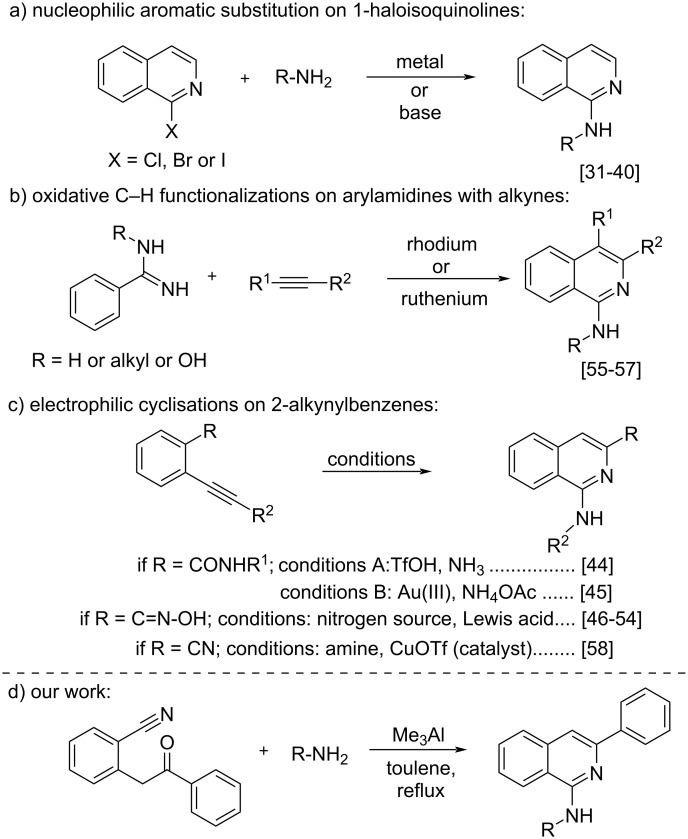
Comparison of our work with the previous approaches for the synthesis of 1-aminoisoquinolines.

Given the pharmacological promiscuity of this scaffold, extensive efforts from different groups led to the development of several approaches for the efficient construction of these heterocyclic frameworks (Scheme 1). Traditional preparations for 1-aminoisoquinolines include nucleophilic substitution of 1-haloisoquinolines with amines either employing a base [31–35] or a transition metal catalyst [36–40]. However, pre-functionalization of isoquinolines to the corresponding halogenated isoquinolines is the main limitation associated with these protocols as they require noxious halogenated acids for their starting materials preparation. Alternative strategies include, amination of isoquinoline *N*-oxides [41–42], condensation of lithiated *o*‐tolualdehyde *tert*‐butylimines with nitriles [43], electrophilic cyclization of 2-alkynylbenzamides [44–45] or 2-alkynylbenzaldoximes [46–54], oxidative C–H functionalizations (coupling) on aryl and heteroaryl amidines with alkynes catalyzed by either rhodium or ruthenium [55–57], or a metal-catalyzed aminative cyclization of 2-alkynylbenzonitriles with secondary amines [58]. Despite the advantages associated with the aforementioned protocols such as the functional group tolerance and huge substrate scope, they are associated with few limitations including: utilization of metals, transition metals, and difficulties in accessing the starting materials, which provoke the attention of the synthetic community for the development of simple and efficient methodologies towards the construction of these heterocyclic frameworks.

On the other hand, organonitrile, the polar unsaturated carbon−nitrogen multiple bond, recognized as one of the most versatile chemotypes in both the laboratory and industry because of their vital role displayed in various transformations [1–4]. They capture a major area in the synthesis of a wide array of heterocyclic compounds by creating C–C, C–N, C–O and C–S bonds due to their ability to act as electrophiles. The cyano group is considered as a versatile functional group in various organic syntheses because of its participation in various electrophilic, necleophilic and bipolar cycloaddition reactions and also serves as a precursor for the generation of important functional groups like amines, aldehydes, ketones and carboxylic acids. Even though the nitrile functional group is prevalent in the transformation into different functional groups, the synthetic approaches that incorporate the nitrogen atom of the cyano group into heterocyclic products is still challenging for the synthetic community. In an effort to develop a synthetic strategy for 1-aminoisoquinolines with increased selectivity and step economy by minimizing the generation of byproducts, we hypothesized that if suitably tailored benzonitriles **3** were cyclized in an intramolecular fashion by installing nuclophilic nitrogen onto the nitrile functionality would generate 1-aminoisoquinolines. Herein we describe our efforts on a Me_3_Al-mediated nucleophilic addition followed by an intramolecular cyclisation of 2-(2-oxo-2-phenylethyl)benzonitriles with amines to deliver 1-aminoisoquinolines and its successful application in the synthesis of antitumor agent CWJ-a-5.

## Results and Discussion

Initially we targeted the synthesis of 2-(2-oxo-2-phenylethyl)benzonitrile (**3a**) by reacting 2-methylbenzonitrile with the appropriate ester of benzoic acid in the presence of a base. After having the starting material in hand, we commenced our investigations for the synthesis of 1-aminoisoquinolines by treating 2-(2-oxo-2-phenylethyl)benzonitrile (**3a**) with aniline (**4a**) in the presence of different Lewis acids under varying reaction parameters. Formation of no desired product was observed when the reaction was carried out in BF_3_·OEt_2_ in toluene under reflux conditions (Table 1, entry 1). To our delight, the expected product **5a** was formed in 18% yield in the presence of TiCl_4_ (Table 1, entry 2). AlCl_3_ was also found to be inefficient for this transformation under similar reaction conditions yielding the desired product only in 16% yield (Table 1, entry 3). Interestingly, a substantial improvement in the yield of the reaction was observed by switching to Me_3_Al in toluene at 110 °C, delivering 85% of the desired product in 8 h (Table 1, entry 4). Moreover, TMS-OTf was also found to be not much effective as MeAl_3_ leading to generation of the desired product in comparably lesser yields than Me_3_Al (Table 1, entry 5). After identifying the suitable Lewis acid for this transformation, we next moved to optimize other reaction parameters such as solvent and temperature. From the list of solvents tested, it is clear that toluene was the solvent of choice, better than DCM, DCE, THF and dioxane (Table 1, entries 5–9). The temperature of the reaction also has notifiable impact on the yields, where increasing the reaction temperature beyond 110 °C or decreasing the reflux temperature led to a slight decrease in the yields of the product (Table 1, entries 10 and 11). No desired product was observed when the reaction was performed at room temperature (Table 1, entry 12).

**Table 1:** Optimization of the reaction conditions for the synthesis of 1-aminoisoquinolines.^a^

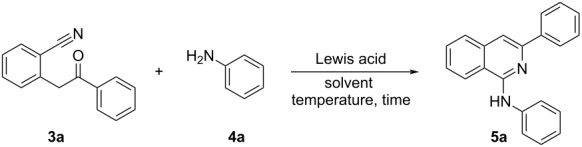					

Entry	Lewis acid (equiv)	Solvent	Temperature (°C)	Time (h)	Yield (%)^b^

1	BF_3_·OEt_2_ (2)	toluene	110	8	–
2	TiCl_4_ (2)	toluene	110	8	18
3	AlCl_3_ (2)	toluene	110	8	16
**4**	**Me** **_3_** **Al (2)**	**toluene**	**110**	**8**	**85**
5	TMS-OTf (2)	toluene	110	8	45
6	Me_3_Al (2)	DCM	40	8	34
7	Me_3_Al (2)	dioxane	100	8	50
8	Me_3_Al (2)	DCE	80	8	48
9	Me_3_Al (2)	THF	60	8	27
10	Me_3_Al (2)	toluene	90	8	63
11	Me_3_Al (2)	toluene	130	8	82
12	Me_3_Al (2)	toluene	rt	12	–

^a^Reaction conditions: **3a** (1 equiv), **4a** (1.5 equiv) in the presence of Lewis acid (2 equiv). ^b^Isolated yield.

**Scheme 2:**
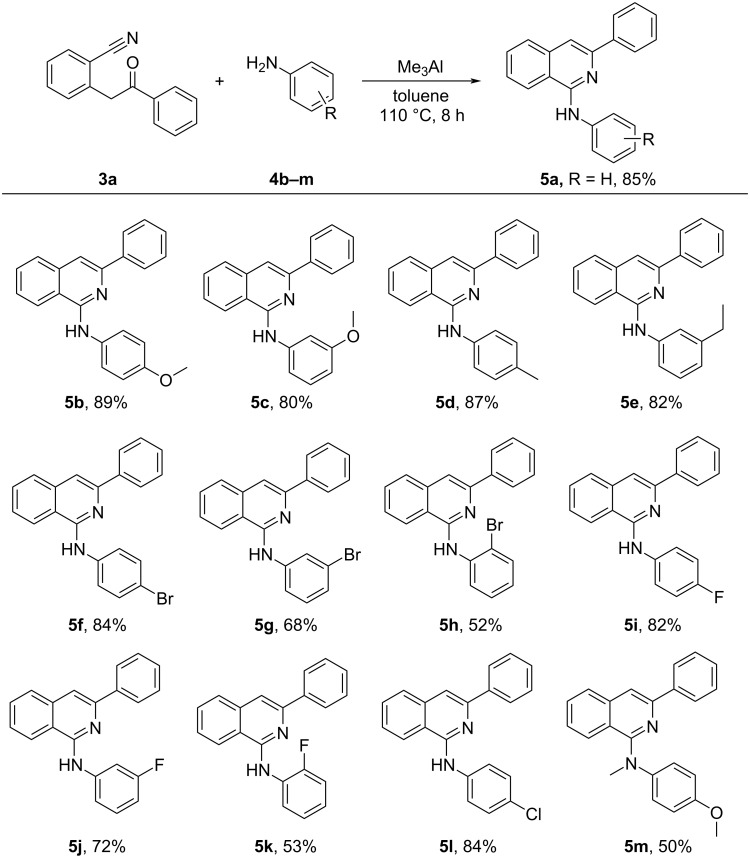
Substrate scope of anilines for the synthesis of 1-aminoisoquinolines (**5a**–**m**). Reaction conditions: **3** (1 equiv), **4** (1.5 equiv), Me_3_Al (2 equiv) in toluene at 110 °C for 8 h. Isolated yields are shown.

With the optimal reaction conditions in hand, we next explored the substrate scope of this protocol. Initially, 2-(2-oxo-2-phenylethyl)benzonitrile (**3a**) was treated with various anilines under the optimized reaction conditions (Scheme 2). The yields of the reactions were not influenced significantly by the electronic effects of the substituents. However, the steric effects of the substituents have influenced the yields of the reaction substantially. Comparably better yields were observed with electron donating substituents than the electron withdrawing halo groups on the aniline ring (Scheme 2, **5b**–**m**). Importantly, the steric effects on the aniline ring have huge impact on the reaction efficiency and efficacy, where *para*- and *meta*-substituents have minimal impact on the yields of the reaction delivering the corresponding products in comparable yields (Scheme 2). While least yields were observed with *ortho*-substituted anilines (Scheme 2, **5h** and **5k**), which can be rationalized by the steric hindrance created by the *ortho*-substituents. It is also worth mentioning that secondary anilines also reacted with 2-(2-oxo-2-phenylethyl)benzonitrile (**3a**) and delivered the corresponding product **5m**, albeit in lesser yields.

**Scheme 3:**
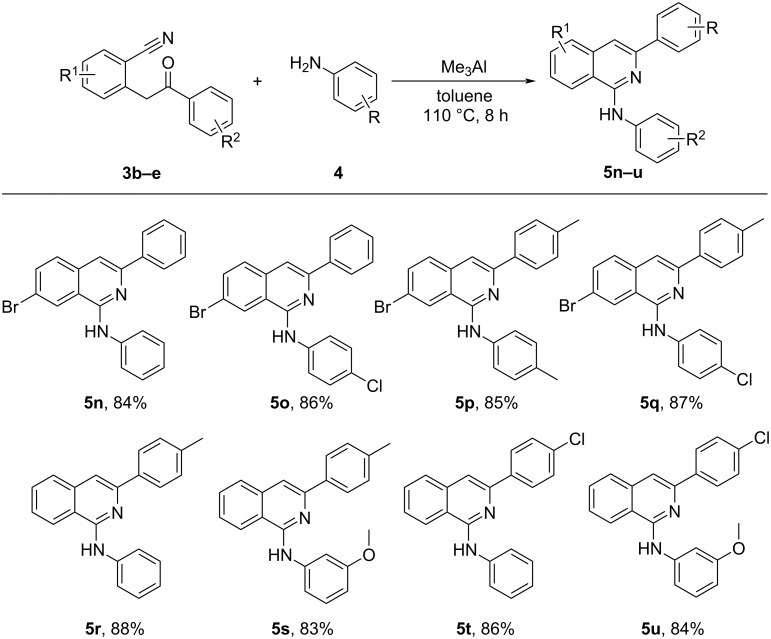
Substrate scope of 2-(2-oxo-2-phenylethyl)benzonitrile (**3b–e**) for the synthesis of 1-aminoisoquinolines (**5n**–**u**). Reaction conditions: **3** (1 equiv), **4** (1.5 equiv), Me_3_Al (2 equiv) in toluene at 110 °C for 8 h. Isolated yields are shown.

Later, the substrate scope of 2-(2-oxo-2-phenylethyl)benzonitriles was also examined. Scheme 3 summarizes the scope of 2-(2-oxo-2-phenylethyl)benzonitriles (**3b**–**e**) towards the domino nucleophilic addition followed by an intramolecular cyclisation of 2-(2-oxo-2-phenylethyl)benzonitriles with amines under optimal reaction conditions. Accordingly, 2-(2-oxo-2-phenylethyl)benzonitriles substituted with various groups (Br, Cl and methyl) on both the benzene rings were treated with different anilines to yield respective products (**5a**–**m**) in good yields (Scheme 3). Examination of the effect of the substituents on the reaction revealed that the substituents on both the benzene rings of 2-(2-oxo-2-phenylethyl)benzonitriles have no significant impact on the yields of the reaction delivering the corresponding products in almost similar yields (**3b**–**e**, Scheme 3).

**Scheme 4:**
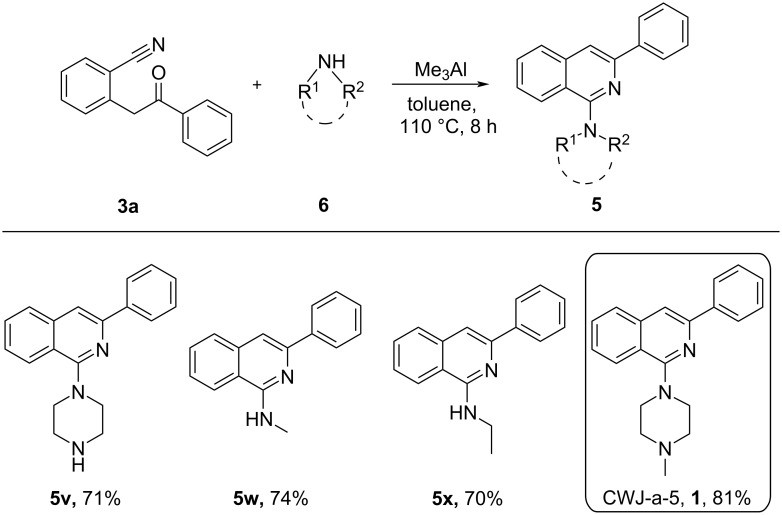
Substrate scope of aliphatic amines for the synthesis of 1-aminoisoquinolines (**5v**–**x**), gram-scale synthesis of antitumor agent CWJ-a-5 (**1**). Reaction conditions: **3** (1 equiv), **4** (1.5 equiv), Me_3_Al (2 equiv) in toluene at 110 °C for 8 h. Isolated yields are shown.

Interestingly, different alkylamines such as methylamine, ethylamine and piperazines were also found to be compatible with the present protocol delivering the corresponding 1-aminoisoquinolines (**5v**–**x**) in good yields (Scheme 4). The synthetic utility of this method was further extended towards the gram-scale synthesis of the antitumor agent CWJ-a-5. Accordingly, 2-(2-oxo-2-phenylethyl)benzonitrile (**3a**) was treated with 1-methylpiperazine (**6**) under the optimized reaction conditions for 8 h, which delivered antitumor agent CWJ-a-5 (**1**) in 81% yield (Scheme 4).

**Scheme 5:**
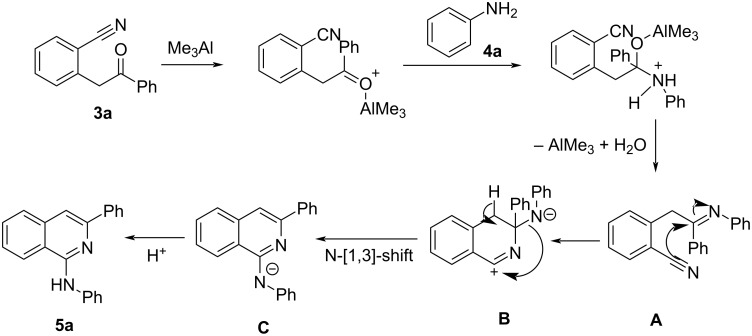
Proposed mechanism for the synthesis of 1-aminoisoquinoline **5a**.

The mechanism for the formation of 1-aminoisoquinolines was depicted in Scheme 5. Initially, 2-(2-oxo-2-phenylethyl)benzonitrile (**3**) condenses with amine/aniline in the presence of Me_3_Al to afford imine intermediate **A.** Intermediate **A** then underdoes an intramolecular cyclisation to afford intermediate **B**. This intermediate **B** then undergoes an *N-*[1,3]-shift leading to the generation of intermediate **C**, which subsequently abstracts a proton to yield the product **5**.

## Conclusion

In summary, an efficient Me_3_Al-mediated domino nucleophilic addition with a subsequent intramolecular cyclisation on 2-(2-oxo-2-phenylethyl)benzonitriles with amines was developed allowing access to widely substituted 1-aminoisoquinolines. Furthermore, the synthetic utility of this protocol was demonstrated in the successful synthesis of the antitumor agent CWJ-a-5 in gram scale. Good to higher yields and a wide substrate scope are the key advantages associated with the current protocol. Further biological investigations of the synthesized compounds are currently underway.

## Supporting Information

File 1 Experimental and analytical data.
