# Supplementary material for: Me3Al-mediated domino nucleophilic addition/intramolecular cyclisation of 2-(2-oxo-2-phenylethyl)benzonitriles with amines; a convenient approach for the synthesis of substituted 1-aminoisoquinolines
Source: Beilstein J Org Chem. 2021 Nov 16;17:2765–72. doi: 10.3762/bjoc.17.186 (PMC8609244; doi:10.3762/bjoc.17.186)

BEILSTEIN JOURNAL OF ORGANIC CHEMISTRY

Supporting Information

for

Me_3Al-mediated domino nucleophilic addition/intramolecular cyclisation of 2-(2-oxo-2-phenylethyl)benzonitriles with amines; a convenient approach for the synthesis of substituted 1-aminoisoquinolines

Krishna M. S. Adusumalli, Lakshmi N. S. Konidena, Hima B. Gandham, Krishnaiah Kumari, Krishna R. Valluru, Satya K. R. Nidasanametla, Venkateswara R. Battula and Hari K. Namballa

Beilstein J. Org. Chem. **2021,** *17,* 2765–2772. doi:10.3762/bjoc.17.186

Experimental and analytical data

License and Terms: This is a supporting information file under the terms of the Creative Commons Attribution License (https://creativecommons.org/licenses/by/4.0). Please note that the reuse, redistribution and reproduction in particular requires that the author(s) and source are credited and that individual graphics may be subject to special legal provisions.
The license is subject to the *Beilstein Journal of Organic Chemistry* terms and conditions: (https://www.beilstein-journals.org/bjoc/terms)

Contents

Experimental.....S2

Materials and methods.....S2

Preparation of starting materials (**3a–e**).....S2

Spectral data for starting materials (**3a–e**).....S2–S3

Experimental procedures and characterization of compounds (**5a–u**).....S3–S9

^{1}H NMR spectra of starting materials (**3a–e**).....S10–S12

^{1}H NMR and ^{13}C NMR spectra of compounds (**5a–u**).....S13–S41

Experimental

1. Materials and methods

Reagents were commercially available (Sigma-Aldrich) with analytical grade and used as purchased without further purification. Solvents were purified according to well-known laboratory methods and freshly distilled prior to use. All reactions were carried out using flame-dried glassware and under an inert atmosphere (dry N_2). Reaction mixtures were monitored by thin layer chromatography (TLC) using silica gel 60-F_{254} plates (Merck, Italy). Spots on the TLC plates were visualized with a UV lamp (254 nm) and by spraying with 0.2% ninhydrin in ethanol and charring after elution. Nuclear magnetic resonance (^{1}H and ^{13}C NMR) spectra were recorded on a 400 MHz spectrometer (Bruker) in DMSO using TMS as an internal standard. Chemical shifts (δ) are reported in ppm, coupling constants (*J* values) are reported in Hertz (Hz) and the peak patterns are indicated as follows: s, singlet; d, doublet; t, triplet; q, quintet; dd, doublet of doublet; dt, doublet of triplet. The IR values are reported in reciprocal centimetres (cm^{-1}) using a Bruker Alpha FT-IR spectrometer. Mass spectra were recorded in a LCQ Fleet mass spectrometer. Mass spectral data were compiled using MS (ESI).

2. Preparation of starting materials:

2.1. General procedure for the preparation of 2-(2-oxo-2-phenylethyl)benzonitrile (3a):

To a stirred slurry of NaH (60% dispersion in mineral oil, 6.83 g, 170.8 mmol) in DME (50 mL) were added 2-methylbenzonitrile (5 g, 42.7 mmol) and methyl benzoate (6.4 g, 46.9 mmol) and stirred at 60 °C for 15 min, followed by addition of catalytic amount of MeOH, the resulting reaction mixture was refluxed for 10 h. After completion of the reaction (monitored by TLC), the mixture was cooled to 0 °C, water (20 mL) was added to quench the reaction and then 2 M HCL (20 mL) was added and extracted with ethyl acetate (2 × 50 mL). The combined organic layer was washed with water, brine, dried over Na_2SO_4 and concentrated in vacuo and the resulting product was purified by column chromatography (silica gel, 100–200 mesh, 4–6% ethyl acetate/hexane) to afford **3a.**

In analogous way compounds **3b–e**[1] were synthesized.

Spectral data for 2-(2-oxo-2-phenylethyl)benzonitrile (3a)[2]**:**

Off white solid, 62%, **TLC** (eluent: ethyl acetate/pet ether 1:9 v/v): $R_{f=}$ 0.45; **^{1}H NMR** (400 MHz, DMSO) δ: 8.04 (d, *J* = 7.6 Hz, 2H), 7.82 (d, *J* = 7.2 Hz, 1H), 7.56 - 7.62 (m, 2H), 7.47 - 7.54 (m, 2H), 7.36 - 7.40 (m, 2H), 4.54 (s, 2H); **ESI-MS**: $[M+H]^+$ 222.24.

Spectral data for 5-bromo-2-(2-oxo-2-phenylethyl)benzonitrile (3b):

Off white solid, 48%, **TLC** (eluent: ethyl acetate/pet ether 1:9 v/v): $R_{f=}$ 0.4; **^{1}H NMR** (500 MHz, $CDCl_3$) δ: 8.03 - 8.05 (m, 2H), 7.82 (d, J = 2.0 Hz,1H), 7.69 - 7.71 (m, 2H), 7.50 - 7.53 (m, 2H), 7.27 (s, 1H), 4.51 (s, 2H); **ESI-MS**: $[M+H]^+$ 314.04.

Spectral data for 5-bromo-2-(2-oxo-2-(*p*-tolyl)ethyl)benzonitrile (3c):

Off white solid, 62%, **TLC** (eluent: ethyl acetate/pet ether 1:9 v/v): $R_{f=}$ 0.45; **^{1}H NMR** (500 MHz, $CDCl_3$) δ: 7.93 (d,J = 8.0 Hz, 1H), 7.81 (d,J = 2.0 Hz, 1H), 7.68 - 7.70 (m, 1H), 7.30 (d,J = 7.5 Hz, 1H), 7.27 (s, 1H), 4.47 (s, 2H), 2.43 (s, 3H); **ESI-MS**: $[M+H]^+$ 300.16.

Spectral data for 2-(2-oxo-2-(*p*-tolyl)ethyl)benzonitrile (3d):

Off white solid, 60%, **TLC** (eluent: ethyl acetate/pet ether 1:9 v/v): $R_{f=}$ 0.5; **^{1}H NMR** (400 MHz, $CDCl_3$) δ: 7.95 (d, J = 8.4 Hz, 1H), 7.67 - 7.70 (m, 1H), 7.54 - 7.58 (m, 1H), 7.36 - 7.40 (m, 2H), 7.29 - 7.31 (m, 2H), 4.52 (s, 2H), 2.43 (s, 3H); **ESI-MS**: $[M+H]^+$ 236.27.

Spectral data for 2-(2-(4-chlorophenyl)-2-oxoethyl)benzonitrile (3e):

pale yellow solid, 61%, **TLC** (eluent: ethyl acetate/pet ether 1:9 v/v): $R_{f=}$ 0.5; **^{1}H NMR** (500 MHz, $CDCl_3$) δ: 7.97 - 8.00 (m, 2H), 7.67 - 7.70 (m, 1H), 7.55 - 7.59 (m, 1H), 7.45 - 7.49 (m, 2H), 7.36 - 7.42 (m, 2H), 4.51 (s, 2H); **ESI-MS**: $[M+H]^+$ 256.42.

3. Experimental procedures and characterization of compounds:

3.1. General procedure for the one pot synthesis of compounds (5a–u):

To a magnetically stirred solution of aniline (**4a**, 310 mg, 3.4 mmol) in toluene (5 mL) was added trimethyl aluminium (2M in toluene, 2.3 mL, 2 mmol) at 0 °C, and then stirred at rt for 1 h, followed by the addition of 2-(2-oxo-2-phenylethyl)benzonitrile (**3a**, 500 mg, 2.26 mmol) at the same temperature and stirred for another 1 h, then the reaction mixture was heated at 110 °C for 8 h. After completion of the reaction (monitored by TLC), reaction mixture was cooled to 0 °C, water (20 mL) was added and extracted with ethyl acetate (2 × 15 mL). The combined organic layer was washed with water, brine, dried over Na_2SO_4 and concentrated under reduced pressure, the crude compound was purified by column chromatography (silica gel, 100–200 mesh, 5–8% methanol/DCM) to afford N,3-diphenylisoquinolin-1-amine (**5a**).

In analogous way compounds **5b–u** were synthesized.

1-(4-Methylpiperazin-1-yl)-3-phenylisoquinoline (CWJ-a-5, 1): Off white solid, 81%, **M.P:** 72- 74 °C; **TLC** (eluent: MeOH/DCM 10:90 v/v): R_f = 0.2; **IR** (KBr): 3419, 3058, 2920, 2832, 2794, 1615, 1560, 1407, 1265, 1063, 1141, 771, 690 cm^{-1}; **^{1}H NMR** (500 MHz, $CDCl_3$) δ: 8.16 - 8.18 (m, 2H), 8.07 (d, J = 8.0 Hz, 1H), 7.8 (d, J = 8.0 Hz, 1H), 7.71 (s, 1H), 7.57 - 7.60 (m, 1H), 7.45 - 7.49 (m, 3H), 7.36 – 7.39 (m, 1H), 3.6 (bs, 4H), 2.75 (bs, 4H), 2.43 (s,

3H); ^{13}**C NMR** (100 MHz, $CDCl_3$) δ: 160.4, 148.2, 139.6, 139.0, 129.6, 128.5, 128.2, 127.6, 126.6, 125.7, 125.3, 120.5, 111.2, 55.0, 50.8, 46.0; **ESI-MS**: $[M+H]^+$ 304.32.

***N*,3-Diphenylisoquinolin-1-amine (5a)**[3]**:** Off white solid, 85%, **M.P:** 280 °C; **TLC** (eluent: MeOH/DCM 10:90 v/v): R_f = 0.3; **IR** (KBr): 3776, 3706, 3044, 1616, 1478, 760 cm^{-1}; ^{1}**H NMR** (400 MHz, DMSO) δ: 8.76 (d, *J* = 8.4 Hz, 1H), 8.02 - 8.08 (m, 2H), 7.83 -7.87 (m, 1H), 7.51 - 7.53 (m, 2H), 7.39 - 7.47 (m, 3H), 7.37 (s, 1H), 7.23 (s, 5H); ^{13}**C NMR** (100 MHz, DMSO) δ: 155.3, 142.2, 136.1, 135.1, 134.0, 130.3, 130.1, 129.8, 128.9, 128.7, 127.6, 126.2, 117.8, 112.8; **ESI-MS:** $[M+H]^+$ 297.38.

***N*-(4-Methoxyphenyl)-3-phenylisoquinolin-1-amine (5b):** Off white solid, 89%, **M.P:** 276 °C; **TLC** (eluent: MeOH/DCM 10:90 v/v): R_f = 0.3; **IR** (KBr): 3609, 3485, 3065, 1971, 1627, 1508, 1257, 1028, 764 cm^{-1}; ^{1}**H NMR** (400 MHz, DMSO) δ: 9.87 (bs, 1H), 8.90 (d, *J*= 7.6 Hz, 1H), 8.03 -8.07 (m, 2H), 7.82 -7.85 (m, 1H), 7.43 (d, *J* = 8.4 Hz, 2H), 7.35 (s, 1H), 7.26 (s, 5H), 6.98 (d, *J* = 8.8 Hz, 2H), 3.73 (s, 3H); ^{13}**C NMR** (100 MHz, DMSO) δ: 160.0, 155.7, 142.7, 135.8, 135.0, 134.2, 130.1, 129.8, 128.8, 128.7, 128.6, 127.7, 127.6, 126.2, 117.7, 115.2, 112.7, 55.3; **ESI-MS**: $[M+H]^+$ 327.48.

***N*-(3-Methoxyphenyl)-3-phenylisoquinolin-1-amine (5c):** Off white solid, 80%, **M.P:** 254 °C; **TLC** (eluent: MeOH/DCM 10:90 v/v): R_f = 0.3; **IR** (KBr): 3447, 3271, 3020, 2762, 1969, 1641, 1486, 1047, 703 cm^{-1}; ^{1}**H NMR** (400 MHz, DMSO) δ: 9.79 (bs, 1H), 8.88 (d, *J*= 8.4 Hz, 1H), 8.03 - 8.08 (m, 2H), 7.83 -7.87 (m, 1H), 7.24 - 7.37 (m, 8H), 7.04 - 7.06 (m, 1H), 6.95 - 6.97 (m, 1H), 3.7 (s, 3H); ^{13}**C NMR** (100 MHz, DMSO) δ: 160.2, 155.2, 142.2, 136.9, 135.7, 135.1, 134.0, 130.9, 129.8, 128.9, 128.8, 127.6, 126.1, 120.7, 117.7, 116.2, 114.8, 112.8, 55.4; **ESI-MS:** $[M+H]^+$ 327.51.

3-Phenyl-*N*-(*p*-tolyl)isoquinolin-1-amine (5d): Off white solid, 87%, **M.P:** 296 °C; **TLC** (eluent: MeOH/DCM 10:90 v/v): R_f = 0.25; **IR** (KBr): 3743, 3416, 3043, 1640, 1514, 1027, 765 cm^{-1}; ^{1}**H NMR** (400 MHz, DMSO) δ: 9.90 (bs, 1H), 8.90 (d, *J*= 8.4 Hz, 1H), 8.02 - 8.07 (m, 2H), 7.82 -7.85 (m, 1H), 7.36 - 7.40 (m, 3H), 7.25 - 7.27 (m, 7H), 2.27 (s, 3H); ^{13}**C NMR** (100 MHz, DMSO) δ: 155.4, 142.3, 139.7, 135.7, 135.0, 134.0, 133.5, 130.6, 129.7, 128.8, 128.7, 128.5, 127.6, 127.5, 126.2, 117.6, 112.8, 20.7; **ESI-MS:** $[M+H]^+$ 311.12.

***N*-(3-Ethylphenyl)-3-phenylisoquinolin-1-amine (5e):** Off white solid, 82%, **M.P:** 248 °C; **TLC** (eluent: MeOH/DCM 10:90 v/v): R_f = 0.3; **IR** (KBr): 3432, 3038, 2349,1645, 1506, 1164,756 cm^{-1}; ^{1}**H NMR** (500 MHz, DMSO) δ: 9.67 (bs, 1H), 8.76 (d, *J*= 8.5Hz, 1H), 8.02 - 8.08 (m, 2H), 7.84 -7.87 (m, 1H), 7.32 - 7.38 (m, 4H), 7.19 - 7.24 (m, 6H), 2.49 - 2.50 (m, 2H),

1.04 (m, 3H); **^{13}C NMR** (100 MHz, DMSO) δ: 155.2, 145.9, 142.3, 136.0, 135.7, 135.1, 134.0, 129.9, 129.8, 129.7, 128.9, 128.6, 128.3, 127.6, 127.5, 126.3, 125.9, 117.7, 112.8, 27.9, 15.1; **ESI-MS**: $[M+H]^+$ 325.19

***N*-(4-Bromophenyl)-3-phenylisoquinolin-1-amine (5f):** Off white solid, 84%, **M.P:** 316 °C; **TLC** (eluent: MeOH/DCM 10:90 v/v): R_f = 0.25; **IR** (KBr): 3743, 3434, 3025, 2925, 1642, 1477, 758cm^{-1}; **^{1}H NMR** (400 MHz, DMSO) δ: 9.71 (bs, 1H), 8.94(d, *J*= 8.4 Hz, 1H), 8.02 - 8.07 (m, 2H), 7.82 - 7.86 (m, 1H), 7.66 (d, *J*= 8.4 Hz, 2H), 7.51 (d, *J*= 8.4 Hz, 2H), 7.36 (s, 1H), 7.25 - 7.27 (m, 5H); **^{13}C NMR** (100 MHz, DMSO) δ: 155.4, 141.9, 135.7, 135.5, 135.12, 133.8, 133.2, 131.1, 129.8, 128.90, 127.80, 127.5, 126.3, 123.7, 117.8, 112.7; **ESI-MS**: $[M+2H]^+$ 377.31.

***N*-(3-Bromophenyl)-3-phenylisoquinolin-1-amine (5g):** Off white solid, 68%, **M.P:** 300 °C; **TLC** (eluent: MeOH/DCM 10:90 v/v): R_f = 0.25; **IR** (KBr): 3430, 3034, 2916, 2363, 1644, 1475, 1072, 770 cm^{-1}; **^{1}H NMR** (500 MHz, DMSO) δ: 9.73 (bs, 1H), 8.77 (d, *J*= 8.0 Hz, 1H), 8.02 - 8.21 (m, 2H), 7.85 - 7.88 (m, 1H), 7.55 - 7.62 (m, 2H), 7.37 - 7.41 (m, 2H), 7.26 - 7.28 (m, 5H); **^{13}C NMR** (100 MHz, DMSO) δ: 155.4, 141.9, 137.4, 135.8, 135.2, 133.7, 133.3, 132.0, 131.8, 129.9, 128.9, 128.2, 127.7, 127.6, 126.3, 122.4, 117.8, 112.7; **ESI-MS:** $[M+2H]^+$ 377.27.

***N*-(2-Bromophenyl)-3-phenylisoquinolin-1-amine (5h):** Off white solid, 52%, **M.P:** 260 °C; **TLC** (eluent: MeOH/DCM 10:90 v/v): R_f = 0.2; **IR** (KBr); 3745, 3426, 3018, 1642, 1480, 760 cm^{-1}; **^{1}H NMR** (500 MHz, DMSO) δ: 9.88 (bs, 1H), 8.80 (d, *J*= 8 Hz, 1H), 8.06 - 8.11 (m, 2H), 7.89 (t, *J* = 7.0 Hz, 1H), 7.83 (d, *J* = 7.0 Hz, 1H), 7.75 (d, *J* = 8.0 Hz, 1H), 7.51 (t, *J* = 7.5 Hz, 1H), 7.44 (s, 1H), 7.38 (t, *J* = 7.5 Hz, 1H), 7.28 - 7.32 (m, 5H); **^{13}C NMR** (100 MHz, DMSO) δ: 154.8, 141.4, 135.7, 135.5, 134.7, 134.0, 132.9, 132.6, 131.7, 129.5, 129.4, 129.2, 129.1, 127.7, 126.5, 121.7, 117.4, 113.2; **ESI-MS**: $[M+2H]^+$ 377.29.

***N*-(4-Fluorophenyl)-3-phenylisoquinolin-1-amine (5i):** Off white solid, 82%, **M.P:** 320 °C; **TLC** (eluent: MeOH/DCM 10:90 v/v): R_f = 0.3; **IR** (KBr): 3745, 3647, 3434, 3038, 1679, 1498, 769 cm^{-1}; **^{1}H NMR** (400 MHz, DMSO) δ: 9.69 (bs, 1H), 8.76 (d, *J*= 8.4 Hz, 1H), 8.02 - 8.09 (m, 2H), 7.84 - 7.88 (m, 1H), 7.59 - 7.63 (m, 2H), 7.24 - 7.38 (m, 8H); **^{13}C NMR** (100 MHz, DMSO) δ:162.5 (d, *J* = 245.6 Hz), 155.6, 142.2, 135.7, 135.1, 133.9, 132.4, 131.5, 131.4, 129.8, 128.8, 127.7, 127.5, 126.3, 117.8, 117.2 (d, *J* = 23.2 Hz), 112.6; **^{19}F NMR** (376 MHz, DMSO) δ: -110.64; **ESI-MS:** $[M+H]^+$ 315.32.

***N*-(3-Fluorophenyl)-3-phenylisoquinolin-1-amine (5j):** Off white solid, 72%, **M.P:** 292 °C; **TLC** (eluent: MeOH/DCM 10:90 v/v): R_f = 0.3; **IR** (KBr): 3740, 3641, 3440, 3030, 1680, 1494, 765 cm^{-1}; **^{1}H NMR** (400 MHz, DMSO) *δ*: 9.54 (bs, 1H), 8.84 (d, *J*= 8.4 Hz, 1H), 8.03 - 8.09 (m, 2H), 7.87 (t, *J* = 6.8 Hz, 1H), 7.58-7.60 (d, *J* = 9.2 Hz, 1H), 7.46 - 7.52 (m, 1H), 7.27 - 7.37 (m, 8H); **^{13}C NMR** (100 MHz, DMSO) *δ*: 162.3 (d, *J* = 244.7 Hz), 155.3, 141.9, 137.3, 135.8, 135.2, 133.7, 131.9, 131.8, 129.8, 129.0, 128.9, 127.8 (d, *J* = 8.6 Hz), 126.1, 125.4, 117.8, 117.7 (d, *J* = 11.7 Hz), 117.4, 117.0, 116.8, 112.7; **^{19}F NMR** (376 MHz, DMSO) δ: -130.99; **ESI-MS:** $[M+H]^+$ 315.45.

***N*-(2-Fluorophenyl)-3-phenylisoquinolin-1-amine (5k):** pale yellow solid, 53%, **M.P:** 265 °C ; **TLC** (eluent: MeOH/DCM 10:90 v/v): R_f = 0.25; **IR** (KBr): 3743, 3491, 3015, 1643, 1495, 767 cm^{-1}; **^{1}H NMR** (400 MHz, DMSO) *δ*: 10.14 (bs, 1H), 8.95 (d, *J*= 8.4 Hz, 1H), 8.04 - 8.12 (m, 2H), 7.88 (t, *J* = 7.6 Hz, 1H), 7.72 (t, *J* = 7.6 Hz, 1H), 7.50 - 7.52 (m, 1H), 7.33 (s, 1H), 7.26 - 7.29 (m, 7H); **^{13}C NMR** (100 MHz, DMSO) δ: 156.6 (d, *J* = 248.2 Hz), 155.5, 155.3, 141.8, 135.7, 135.6, 133.4, 133.1, 130.9, 129.2, 127.9, 127.8, 126.4, 126.1, 123.5, 123.3, 117.5, 117.2 (d, *J* = 19.9 Hz), 113.0; **^{19}F NMR** (376 MHz, DMSO) δ: -120.99; ESI-MS: [M+H]+ 315.12.

***N*-(4-Chlorophenyl)-3-phenylisoquinolin-1-amine (5l):** Off white solid, 84%, **M.P:** 314 °C; **TLC** (eluent: MeOH/DCM 10:90 v/v): R_f = 0.35; **IR** (KBr): 3744, 3436, 3027, 1644, 1485, 1088, 756 cm^{-1}; **^{1}H NMR** (400 MHz, DMSO) *δ*: 9.72 (bs, 1H), 8.78 (d, *J*= 8.4 Hz, 1H), 8.03 - 8.07 (m, 2H), 7.85 - 7.89 (m, 1H), 7.54 - 7.60 (m, 4H), 7.38 - 7.29 (m, 6H); **^{13}C NMR** (125 MHz, DMSO) *δ*: 155.4, 142.0, 135.7, 135.1, 135.1, 134.9, 133.8, 130.9, 130.3, 129.8, 129.0, 128.9, 127.8, 127.7, 126.1, 117.8, 112.8; **ESI-MS**: $[M+H]^+$ 331.10.

***N*-(4-Methoxyphenyl)-*N*-methyl-3-phenylisoquinolin-1-amine (5m):** Light brown solid, 50%, **M.P:** 100 °C; **TLC** (eluent: MeOH/DCM 10:90 v/v): R_f =0.3; **IR** (KBr): 3785, 3411, 3050, 2350, 1593, 1497, 1015, 758 cm^{-1}; **^{1}H NMR** (400 MHz, DMSO) *δ*: 8.24 (d, *J*= 6.8 Hz, 1H), 7.99 (s, 1H), 7.89 - 7.91 (m, 2H), 7.43 - 7.56 (m, 5H), 7.22 - 7.26 (m, 1H), 7.01 - 7.03 (m, 2H), 6.87 - 6.89 (m, 2H), 3.72 (s, 3H), 3.56 (s, 3H); **^{13}C NMR** (100 MHz, DMSO) *δ*: 156.9, 155.9, 146.7, 143.8, 138.9, 138.5, 129.7, 128.6, 128.4, 127.7, 126.3, 126.2, 125.8, 125.0, 120.0, 114.8, 110.8, 55.2, 42.7; **ESI-MS:** $[M+H]^+$ 341.52.

7-Bromo-N,3-diphenylisoquinolin-1-amine (5n): Off white solid, 84%, **M.P:** 296 °C; **TLC** (eluent: MeOH/DCM 10:90 v/v): R_f = 0.25; **IR** (KBr): 3352, 3041, 1635, 1485, 1076, 764 cm^{-1}; **^{1}H NMR** (400 MHz, DMSO) *δ*: 9.86 (bs, 1H), 9.17 (s, 1H), 8.22 - 8.25 (m,1H), 7.99 (d,

J = 8.8 Hz, 1H), 7.39 - 7.51 (m, 6H), 7.23 (s, 5H); ^{13}**C NMR** (100 MHz, DMSO) *δ*: 154.5, 142.8, 137.9, 135.9, 134.7, 133.8, 130.4, 130.2, 129.7, 128.8, 128.8, 128.4, 127.7, 121.9, 119.3, 112.4; **ESI-MS**: $[M+2H]^+$ 377.32.

7-Bromo-*N*-(4-chlorophenyl)-3-phenylisoquinolin-1-amine (5o): Off white solid, 86%, **M.P:** 320 °C; **TLC** (eluent: MeOH/DCM 10:90 v/v): R_f= 0.3; **IR** (KBr): 3744, 3439, 3090, 2362, 1647, 1485, 1087, 762 cm^{-1}; ^{1}**H NMR** (400 MHz, DMSO) *δ*: 9.29 (s, 1H), 8.23 (d, *J* = 8 Hz,1H), 7.99 (d, *J* = 8.4 Hz, 1H), 7.54 - 7.55 (m, 4H), 7.39 (s, 1H), 7.26 (s, 5H); ^{13}**C NMR** (100 MHz, DMSO) *δ*: 154.7, 142.6, 137.9, 135.0, 134.9, 134.7, 133.6, 130.8, 130.3, 129.8, 129.6, 129.0, 128.6, 127.8, 121.8, 119.4, 112.3; **ESI-MS**: $[M+H]^+$ 409.27.

7-Bromo-*N*,3-di-*p*-tolylisoquinolin-1-amine (5p): Off white solid, 85%, **M.P:** 300 °C; **TLC** (eluent: MeOH/DCM 10:90 v/v): R_f= 0.35; **IR** (KBr): 3468, 3028, 2034, 1640, 1511, 1022, 719 cm^{-1}; ^{1}**H NMR** (400 MHz, DMSO) *δ*: 9.33 (bs, 1H), 9.19 (s, 1H), 8.2 - 8.25 (m,1H), 7.97 (d, *J* = 8.8 Hz, 1H), 7.27 - 7.38 (m, 5H), 7.04 - 7.13 (m, 4H), 2.29 (s, 3H), 2.21 (s, 3H); ^{13}**C NMR** (100 MHz, DMSO) *δ*: 154.7, 143.0, 139.9, 138.3, 137.8, 134.8, 133.5, 131.1, 130.8, 129.6, 128.4, 128.3, 128.3, 121.7, 119.2, 112.5, 20.8, 20.7; **ESI-MS:** $[M+H]^+$ 403.35.

7-Bromo-*N*-(4-chlorophenyl)-3-(*p*-tolyl)isoquinolin-1-amine (5q): Off white solid, 87%, **M.P:** 314 °C; **TLC** (eluent: MeOH/DCM 10:90 v/v): R_f= 0.4; **IR** (KBr): 3745, 3442, 3091, 2361, 1648, 1512, 1090, 722 cm^{-1}; ^{1}**H NMR** (400 MHz, DMSO) *δ*: 9.96 (bs, 1H), 9.23 (s, 1H), 8.22 (d, *J* =8.8 Hz, 1H), 7.98 (d, *J* = 8.4 Hz, 1H), 7.56 (s, 4H), 7.34 (s, 1H), 7.07 - 7.13 (m, 4H), 2.23 (s, 3H); ^{13}**C NMR** (100 MHz, DMSO) *δ*: 154.7, 142.7, 138.5, 137.9, 135.3, 135.0, 134.8, 130.8, 130.8, 130.4, 129.7, 128.5, 128.4, 121.8, 119.3, 112.4, 20.7; **ESI-MS:** $[M+H]^+$423.26

***N*-Phenyl-3-(*p*-tolyl)isoquinolin-1-amine (5r):** Off white solid, 88%, **M.P:** 300 °C; **TLC** (eluent: MeOH/DCM 10:90 v/v): R_f = 0.3; **IR** (KBr): 3700, 3625, 3468, 3030, 1675, 1486, 1027, 762 cm^{-1}; ^{1}**H NMR** (500 MHz, DMSO) *δ*: 9.83 (bs, 1H), 8.88 (d, *J* =8.5 Hz, 1H), 8.02 - 8.07 (m, 2H), 7.82 - 7.85 (m, 1H), 7.50 - 7.52 (m, 2H), 7.41 - 7.48 (m, 3H), 7.33 (s, 1H), 7.12 (d, *J* = 8.0 Hz, 2H), 7.03 (d, *J* = 8.0 Hz, 2H), 2.2 (s, 3H); ^{13}**C NMR** (125 MHz, DMSO) *δ*: 155.3, 142.3, 138.2, 136.1, 135.8, 135.1, 131.2, 130.3, 130.2, 129.7, 128.8, 128.2, 127.6, 126.2, 117.6, 112.8, 20.7; **ESI-MS:** $[M+H]^+$ 311.42.

***N*-(3-Methoxyphenyl)-3-(*p*-tolyl)isoquinolin-1-amine (5s):** Off white solid, 83%, **M.P:** 256 °C; **TLC** (eluent: MeOH/DCM 10:90 v/v): R_f = 0.25; **IR** (KBr): 3699, 3603, 3419, 3027, 1643, 1481, 1031, 757 cm^{-1}; ^{1}**H NMR** (400 MHz, DMSO) *δ*: 9.87 (bs, 1H), 8.92 (d, *J* =8.4 Hz, 1H),

8.01 - 8.07 (m, 2H), 7.83 (t, *J* = 6.4 Hz, 1H), 7.17 - 7.36 (m, 5H), 6.96 - 7.06 (m, 4H), 3.71 (s, 3H), 2.22 (s, 3H); **^{13}C NMR** (100 MHz, DMSO) *δ*: 160.3, 155.2, 142.3, 138.2, 137.0, 135.8, 135.0, 131.2, 130.9, 129.7, 128.8, 128.2, 127.5, 126.2, 120.7, 117.6, 116.3, 114.7, 112.8, 55.4, 20.7; **ESI-MS:** $[M+H]^+$ 341.43.

3-(4-Chlorophenyl)-*N*-phenylisoquinolin-1-amine (5t): Off white solid, 86%, **M.P:** 286 °C; **TLC** (eluent: MeOH/DCM 10:90 v/v): R_f = 0.3; **IR** (KBr): 3771, 3429,3042, 1648, 1472, 1080, 764 cm^{-1}; **^{1}H NMR** (500 MHz, DMSO) *δ*: 9.98 (s, 1H), 8.92 (d, *J* =8.5 Hz, 1H), 8.03 - 8.08 (m, 2H), 7.86 (t, *J* = 7.0 Hz, 1H), 7.54 (d, *J* = 7.0 Hz, 2H), 7.42 - 7.49 (m, 3H), 7.39 (s, 1H), 7.27 - 7.32 (m, 4H); **^{13}C NMR** (125 MHz, DMSO) *δ*: 155.3, 141.0, 136.0, 135.6, 135.2, 133.6, 131.7, 130.5, 130.3, 129.1, 128.9, 127.8, 127.7, 126.3, 117.9, 113.0; **ESI-MS:** $[M+H]^+$ 331.12.

3-(4-Chlorophenyl)-*N*-(3-methoxyphenyl)isoquinolin-1-amine (5u): Off white solid, 84%, **M.P:** 270 °C; **TLC** (eluent: MeOH/DCM 10:90 v/v): R_f = 0.25; **IR** (KBr): 3771, 3435, 3024, 1643, 1492, 1093, 763 cm^{-1}; **^{1}H NMR** (400 MHz, DMSO) *δ*: 9.97 (bs, 1H), 8.93 (d, *J* = 8.4Hz, 1H), 8.02 - 8.08 (m, 2H), 7.84 - 7.87 (m, 1H), 7.33 - 7.38 (m, 6H), 7.26 (s, 1H), 6.98 - 7.06 (m, 2H), 3.72 (s, 3H); **^{13}C NMR** (100 MHz, DMSO) *δ*: 160.3, 155.2, 140.9, 136.8, 135.6, 135.1, 133.6, 132.9, 131.7, 131.0, 129.1, 127.7, 127.6, 126.3, 120.7, 117.8, 116.4, 114.7, 112.9, 55.5; **ESI-MS:** $[M+H]^+$ 361.42.

3-Phenyl-1-(piperazin-1-yl)isoquinoline (5v): Off white solid, 71%, **M.P:** 248 °C; **TLC** (eluent: MeOH/DCM 10:90 v/v): R_f = 0.1; **IR** (KBr): 3747, 3421, 3059, 2704, 2459, 1618, 1562, 1392, 1020, 765, 686 cm^{-1}; **^{1}H NMR** (400 MHz, DMSO) *δ*: 9.23 (bs, 1H), 8.20 - 8.22 (m, 2H), 8.15 (d, *J* = 8.4, 1H), 8.10 (s, 1H), 7.99 (d, *J* = 8, 1H), 7.73 – 7.76 (m, 1H), 7.59 - 7.63 (m, 1H), 7.46 - 7.53 (m, 2H), 7.40 – 7.44 (m, 1H),3.64 – 3.65 (m, 4H), 3.38 – 3.41 (m, 4H); **^{13}C NMR** (100 MHz, DMSO) *δ*: 159.3, 146.9, 138.7, 138.6, 130.4, 128.7, 128.5, 127.8, 126.7, 126.2, 125.1, 119.7, 111.9, 47.7, 42.8; **ESI-MS:** $[M+H]^+$ 290.30.

***N*-Methyl-3-phenylisoquinolin-1-amine (5w):** Pale yellow solid, 74%, **M.P:** 290 °C; **TLC** (eluent: MeOH/DCM 10:90 v/v): R_f = 0.3; **IR** (KBr): 3348, 3054, 2715, 1668, 1574, 1447, 1158, 763, 705 cm^{-1}; **^{1}H NMR** (400 MHz, DMSO) *δ*: 9.63 (bs, 1H), 8.88 (d, *J* = 8.4, 1H), 7.94 – 8.00 (m, 5H), 7.27 (s, 1H), 3.60 (s, 3H); **^{13}C NMR** (100 MHz, DMSO) *δ*: 155.0, 142.6, 135.1, 134.4, 134.1, 129.7, 129.2, 128.8, 128.7, 127.4, 125.5, 117.6, 113.2; **ESI-MS:** $[M+H]^+$ 235.15.

***N*-Ethyl-3-phenylisoquinolin-1-amine (5x):** Off white solid, 70%, **M.P:** 296 °C; **TLC** (eluent: MeOH/DCM 10:90 v/v): R_f = 0.3; **IR** (KBr): 3741, 3410, 3057, 1649, 1571, 1381, 1151, 763, 698, 650 cm^{-1}; **^{1}H NMR** (500 MHz, DMSO) δ: 9.61 (bs, 1H), 8.87 (d, *J* = 8.5, 1H), 7.93 – 8.00 (m, 2H), 7.81 (t, *J* = 7.5, 15, 1H), 7.60 (s, 5H), 7.23 (s, 1H), 4.16 (q, *J* = 6.5, 20, 2H), 1.14 (t, *J* = 7, 14, 3H); **^{13}C NMR** (125 MHz, DMSO) δ: 153.6, 142.0, 134.9, 134.6, 134.0, 129.7, 129.4, 128.8, 128.7, 127.4, 125.6, 118.0, 113.8, 44.3, 12.1; **ESI-MS:** $[M+H]^+$ 249.25

References:

1. K. Yashiro, K. Sakata, I. Hachiya, M. Shimizu, *Heterocycles*, 2016, **92**, 2032-2046.
2. T. Wang, N. Jiao, *J. Am. Chem. Soc.* **2013**, *135*(*32*), 11692-11695.
3. W. Li, Y. Wang, T. Lu, *Tetrahedron* **2012**, *68*(*34*), 6843-6848.

^{1}H NMR Spectrum of **3a**

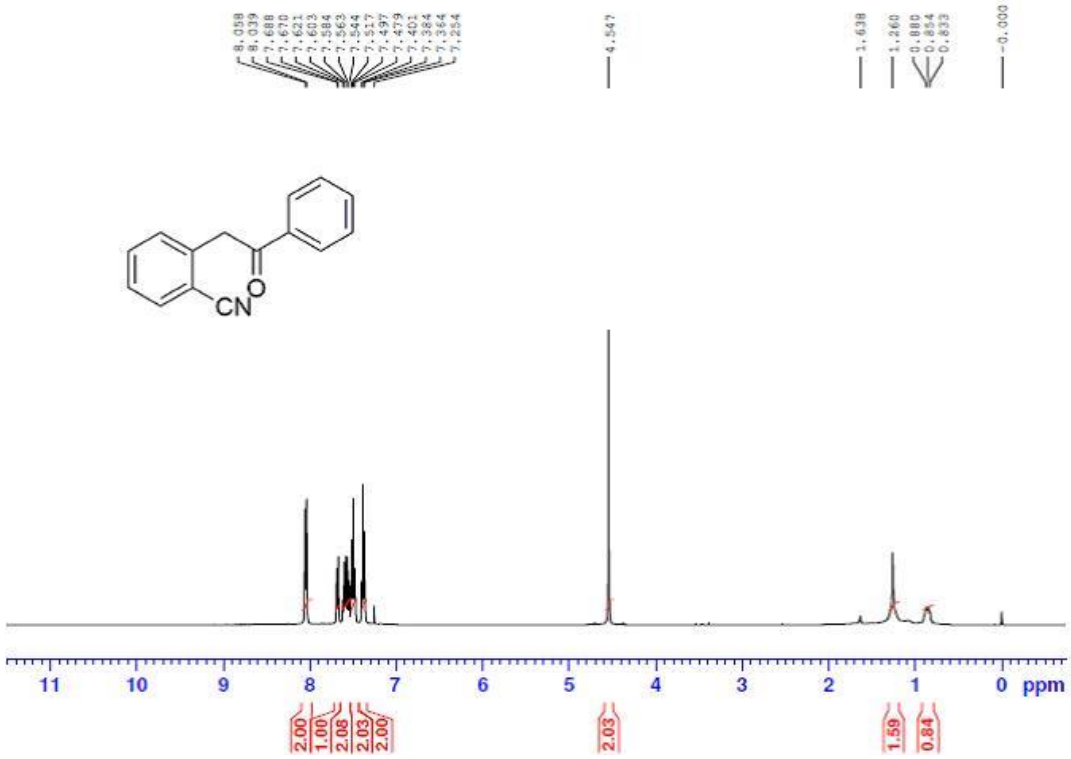

^{1}H NMR Spectrum of **3b**

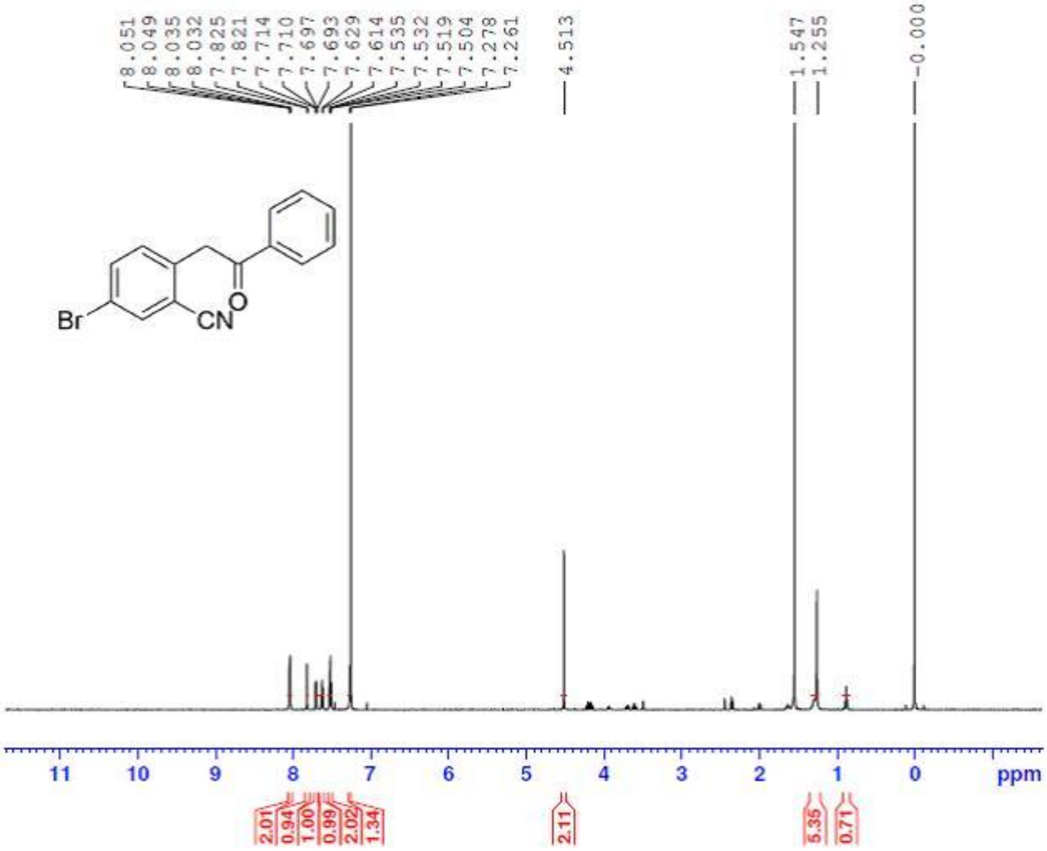

^{1}H NMR Spectrum of **3c**

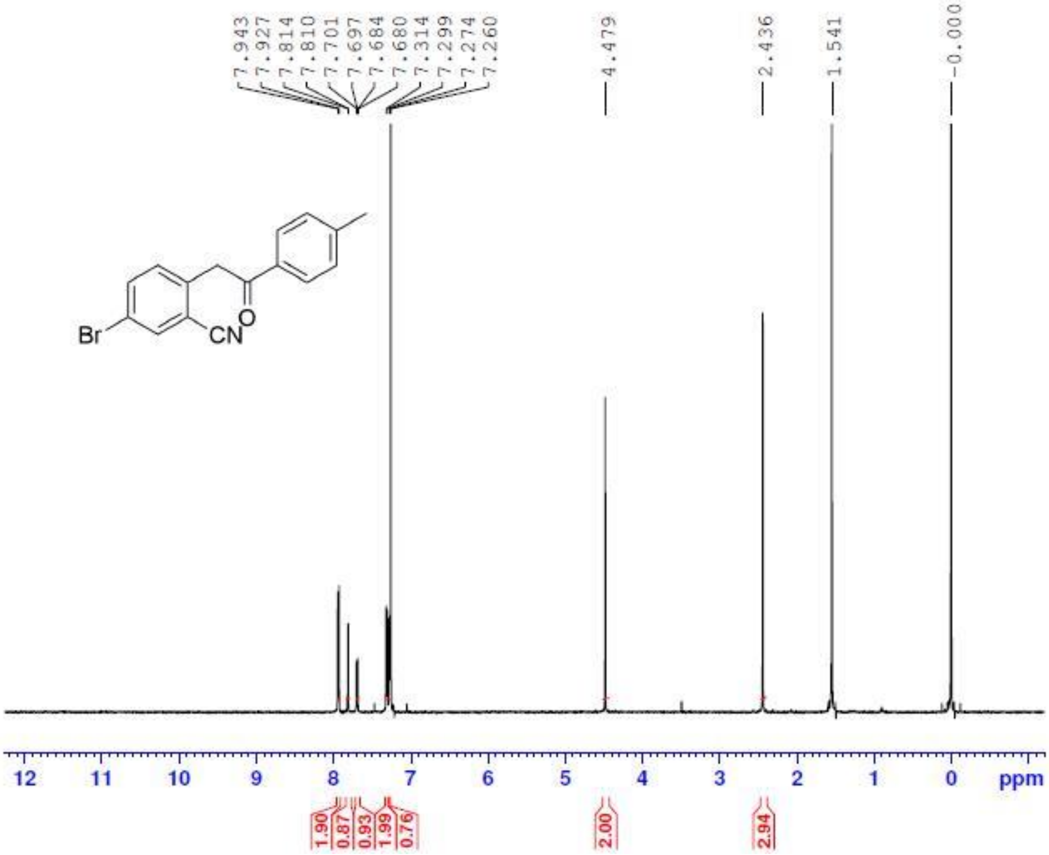

^{1}H NMR Spectrum of **3d**

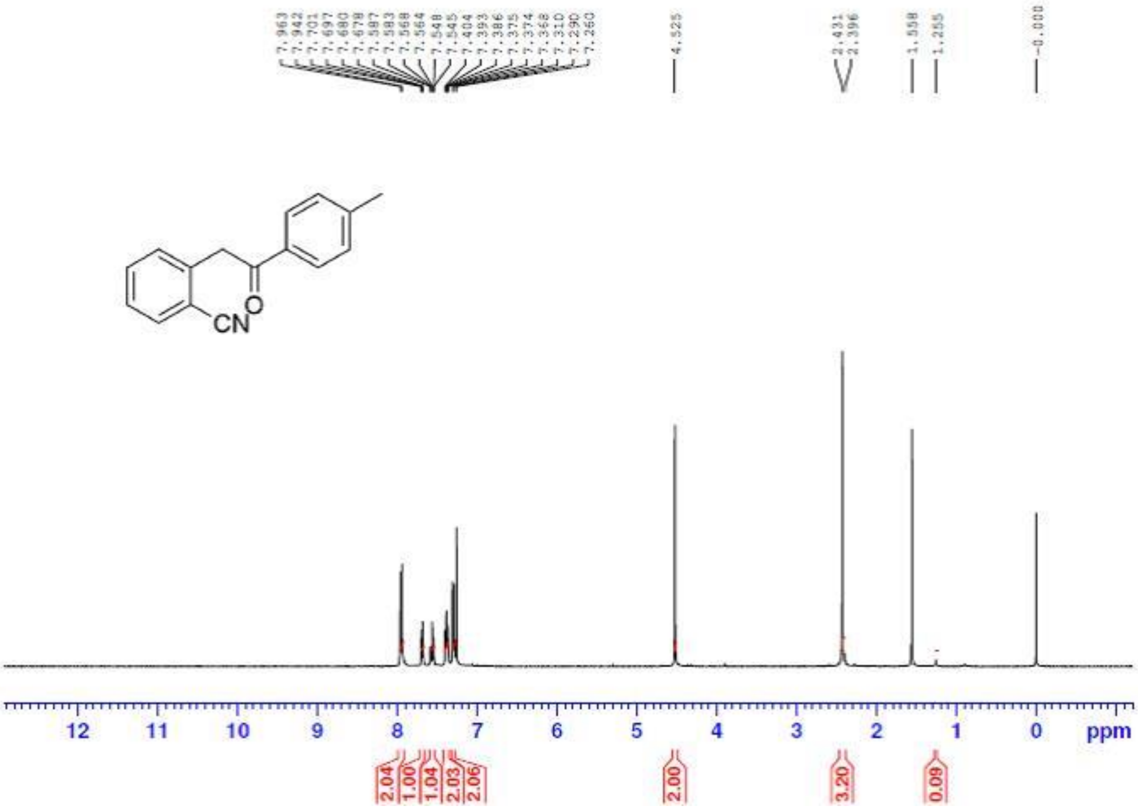

^{1}H NMR Spectrum of **3e**

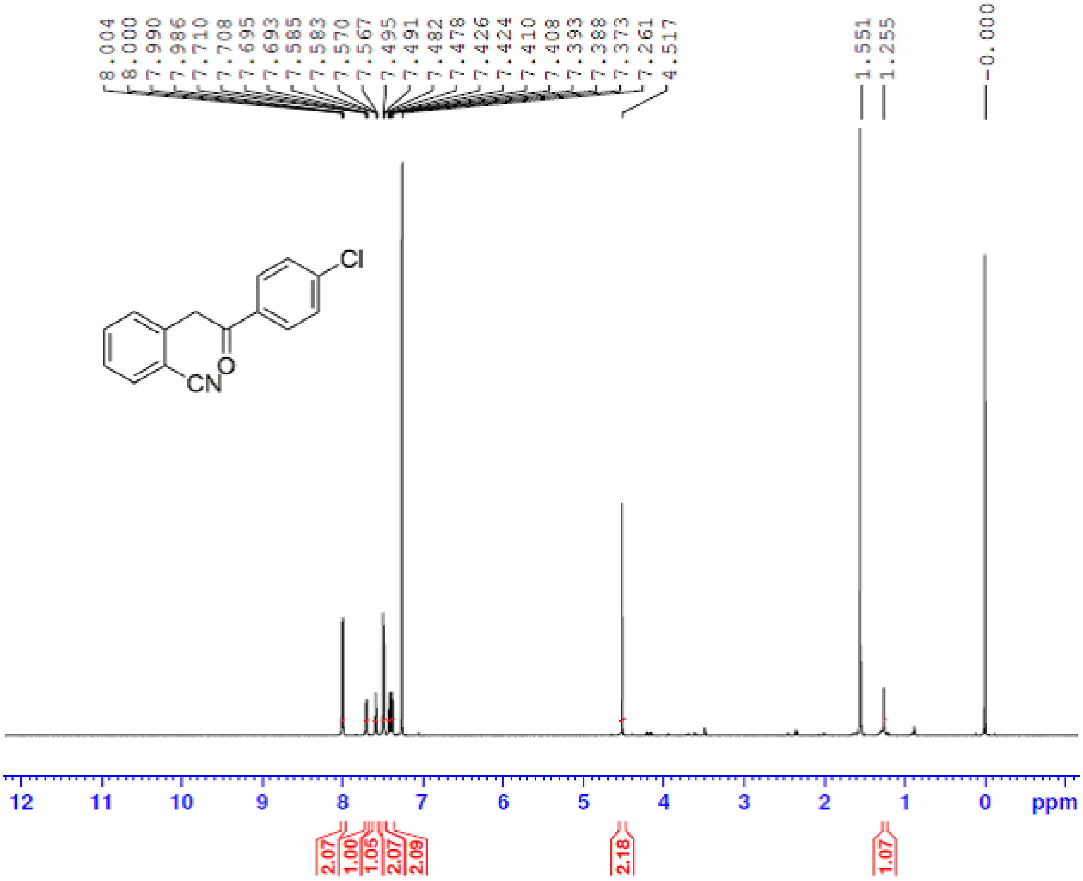

^{1}H NMR Spectrum of **CWJ-a-5**

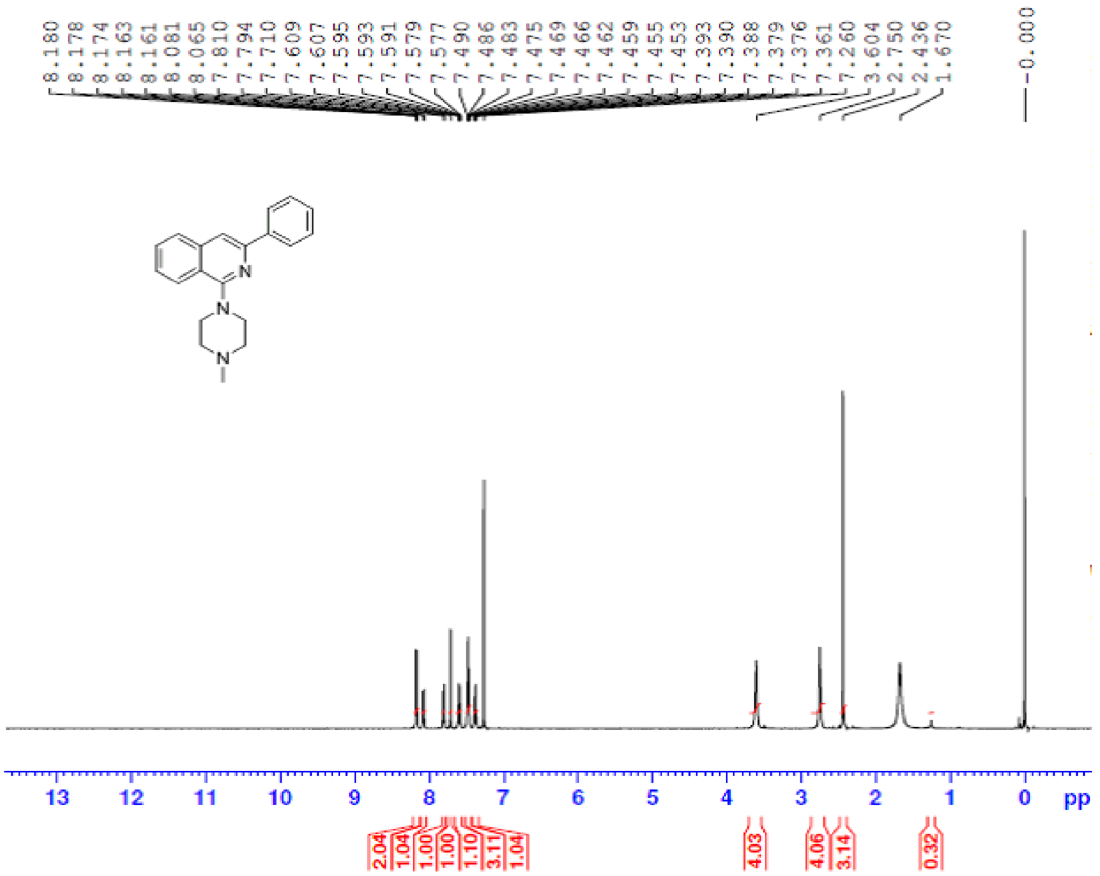

^{13}C NMR Spectrum of **CWJ-a-5**

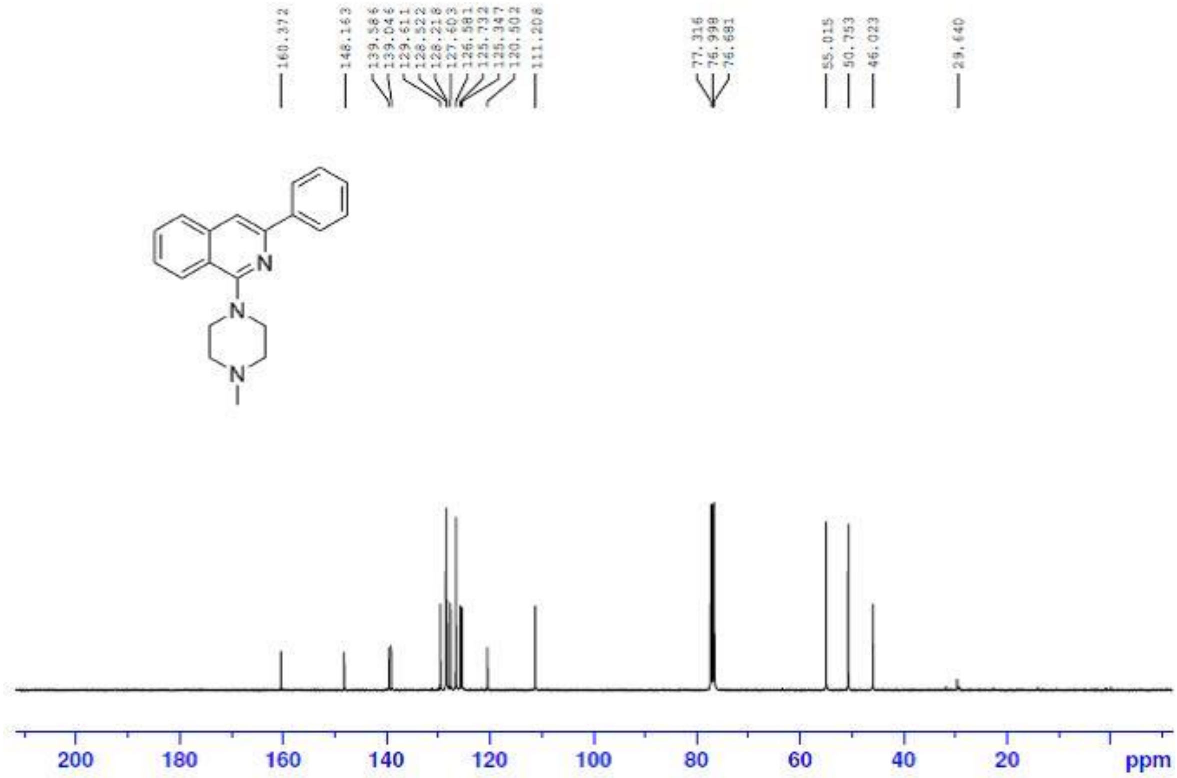

FT-IR Spectrum of **CWJ-a-5**

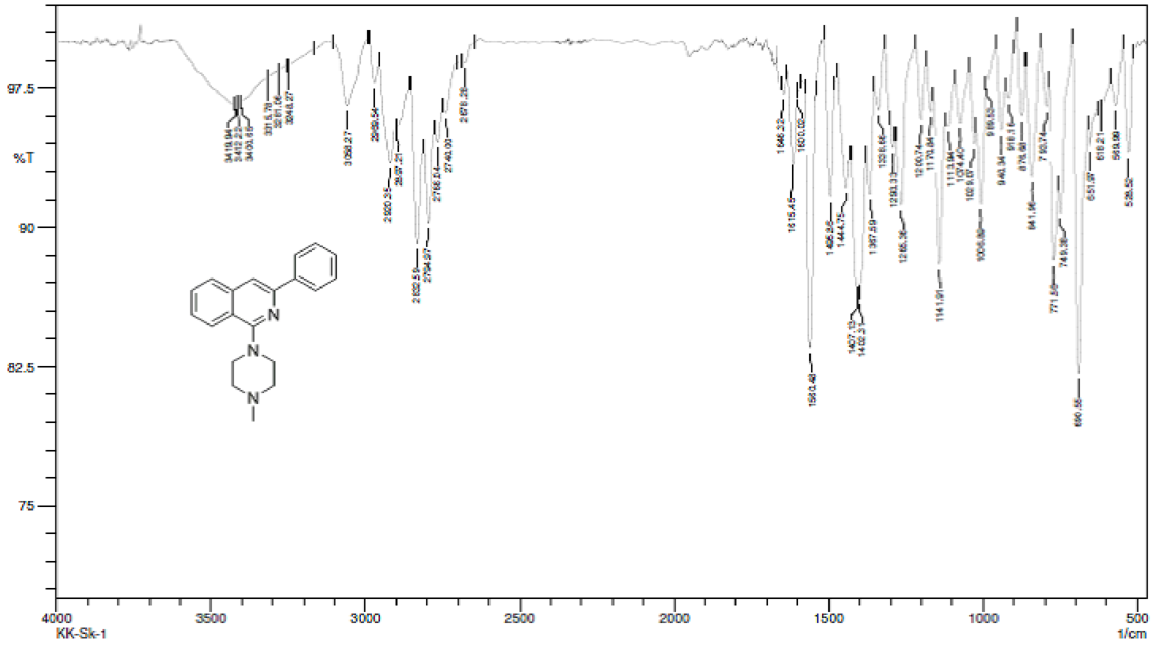

LC-MS Spectrum of **CWJ-a-5**

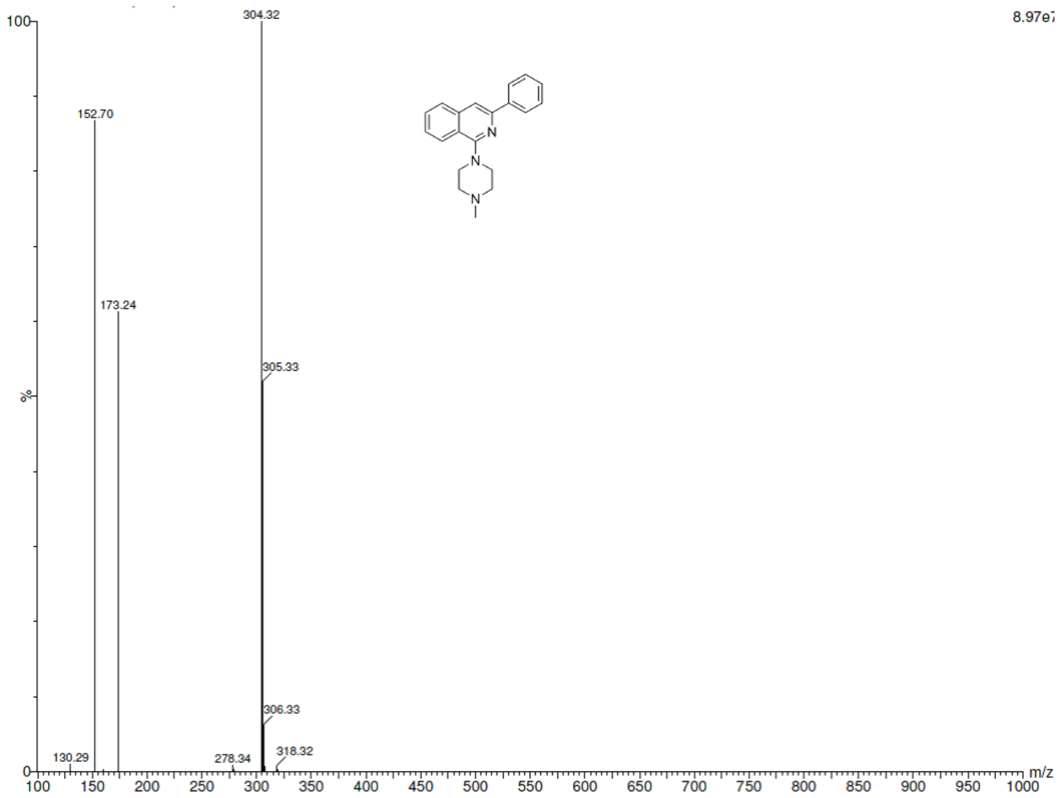

^{1}H NMR Spectrum of **5a**

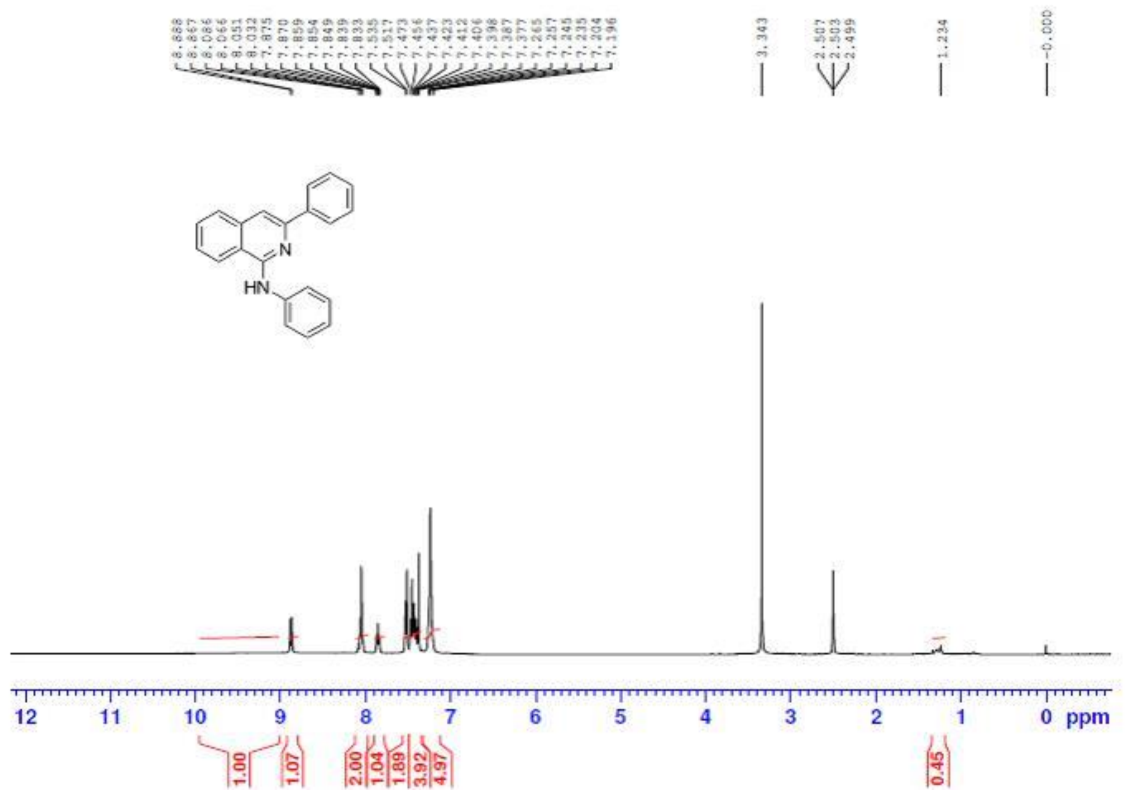

^{13}C NMR Spectrum of **5a**

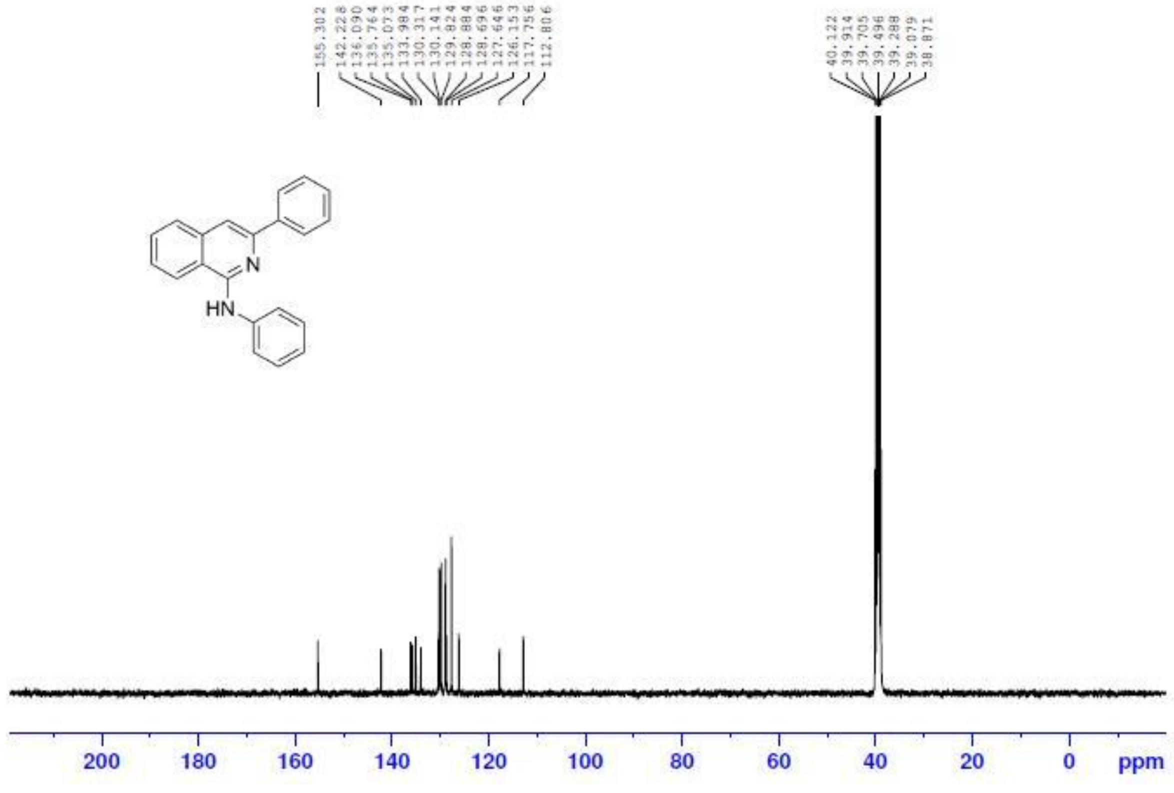

^{1}H NMR Spectrum of **5b**

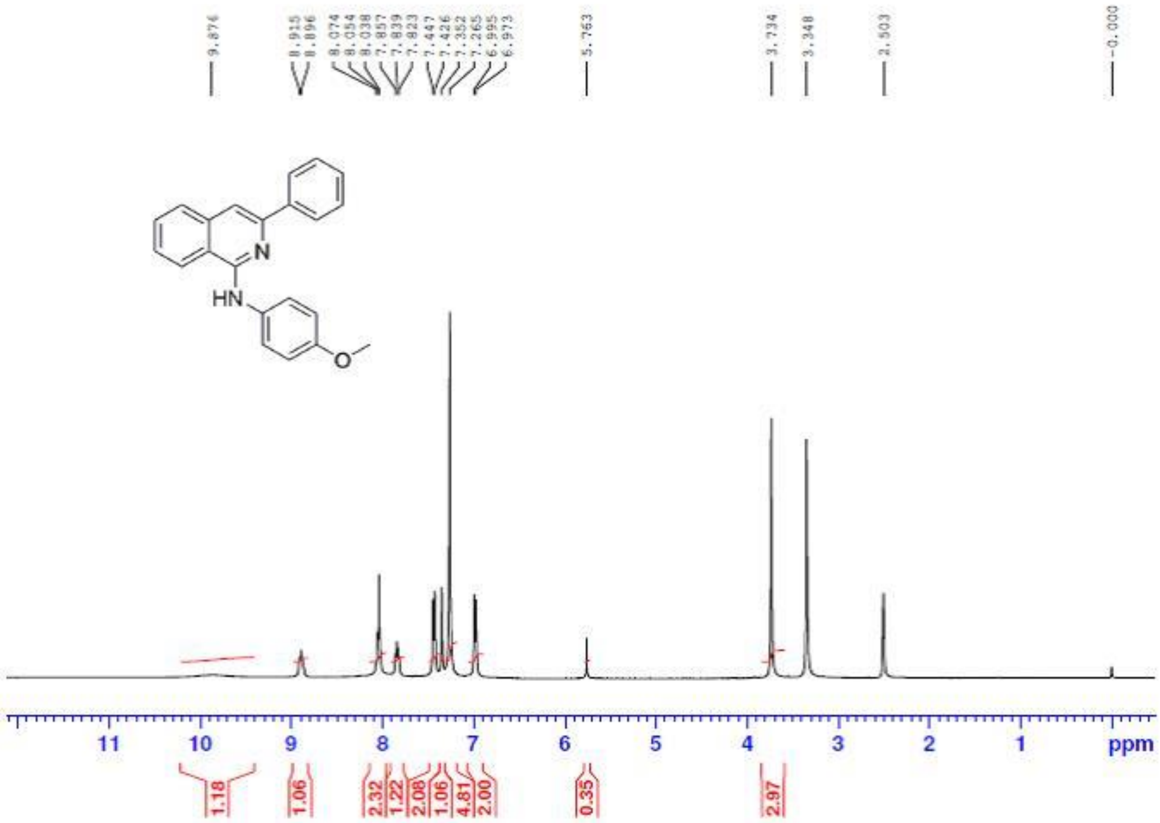

^{13}C NMR Spectrum of **5b**

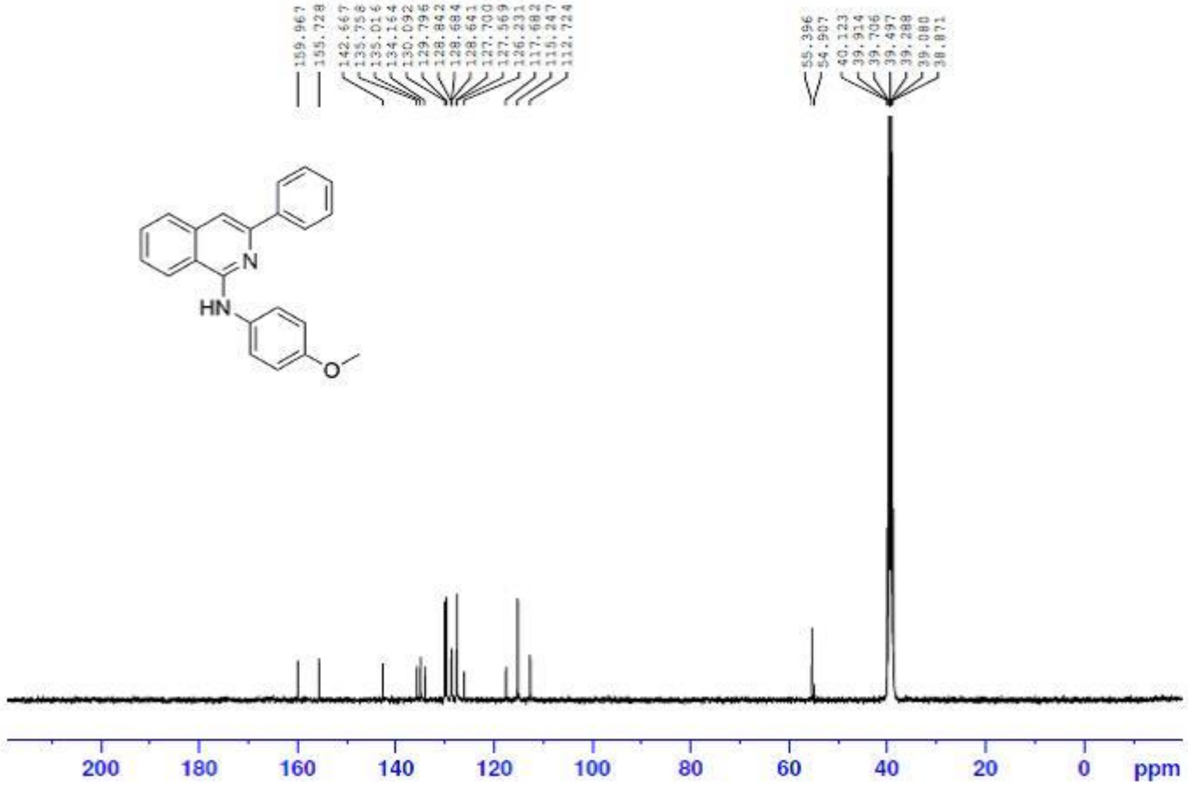

^{1}H NMR Spectrum of **5c**

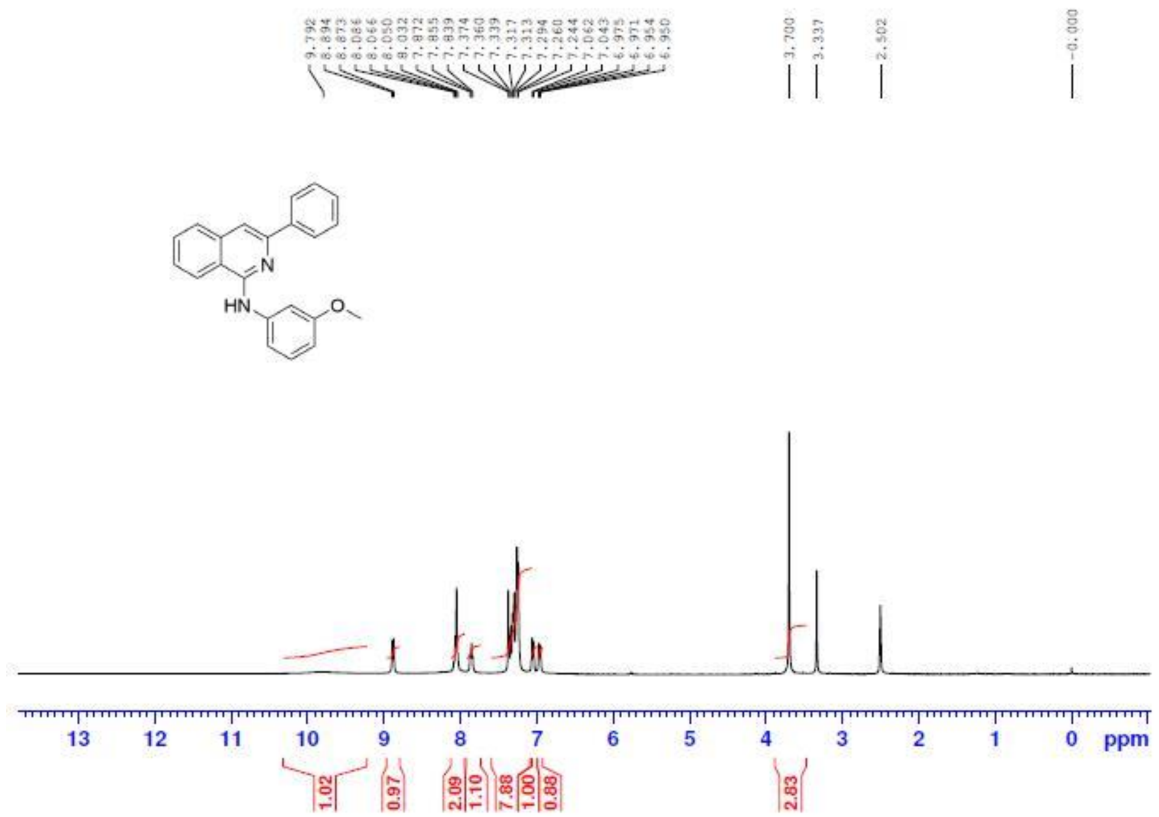

^{13}C NMR Spectrum of **5c**

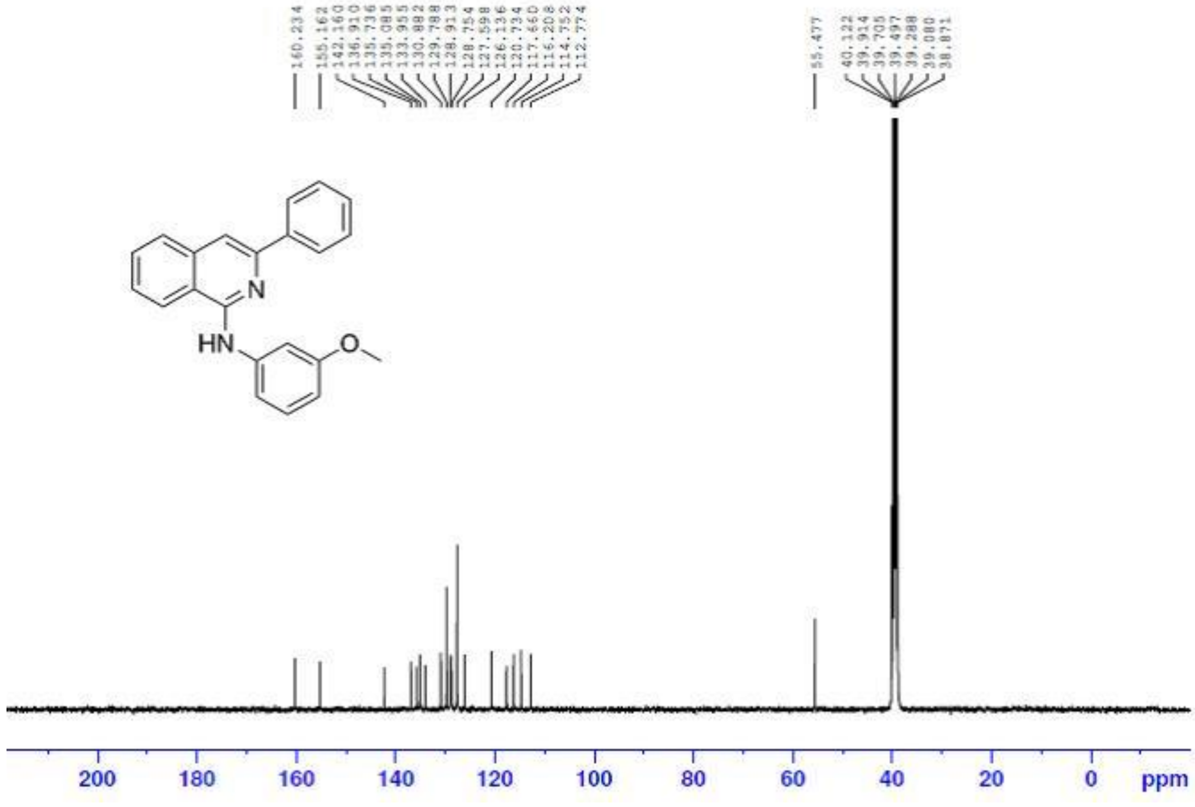

^{1}H NMR Spectrum of **5d**

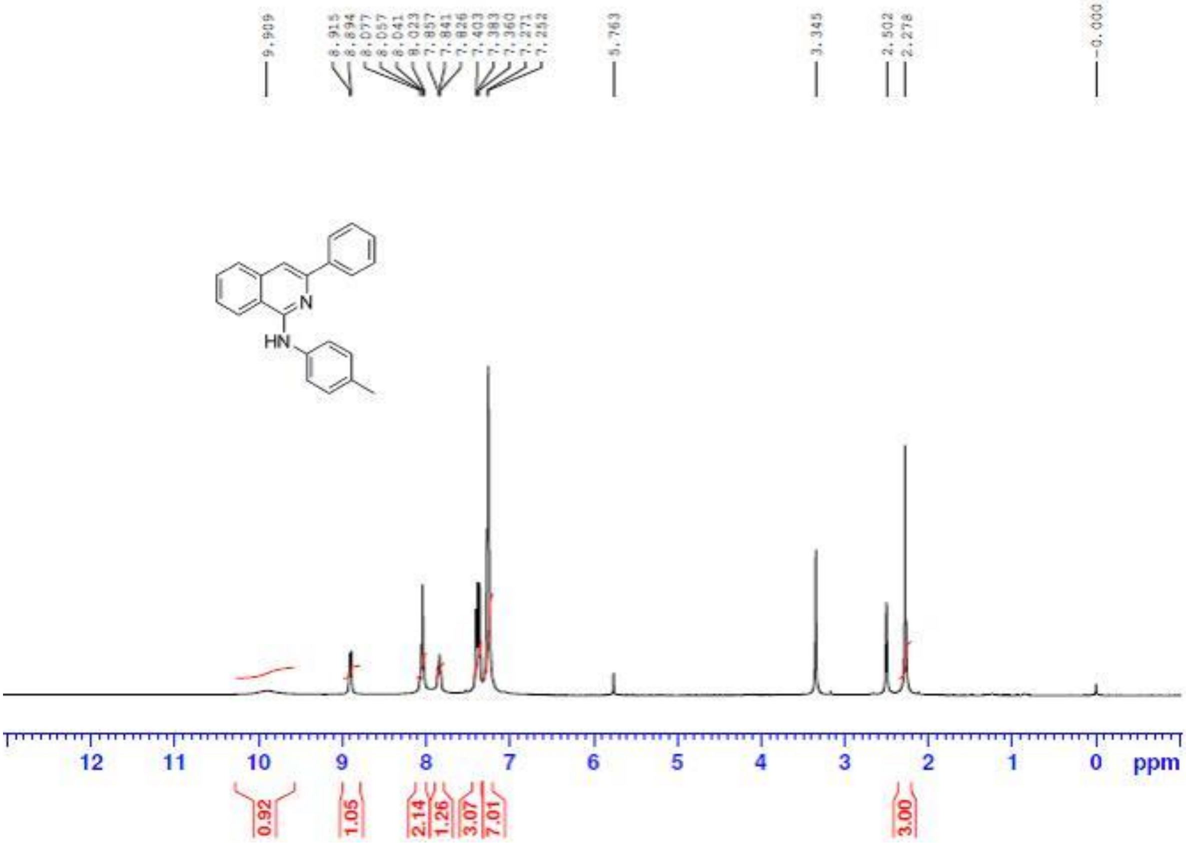

^{13}C NMR Spectrum of **5d**

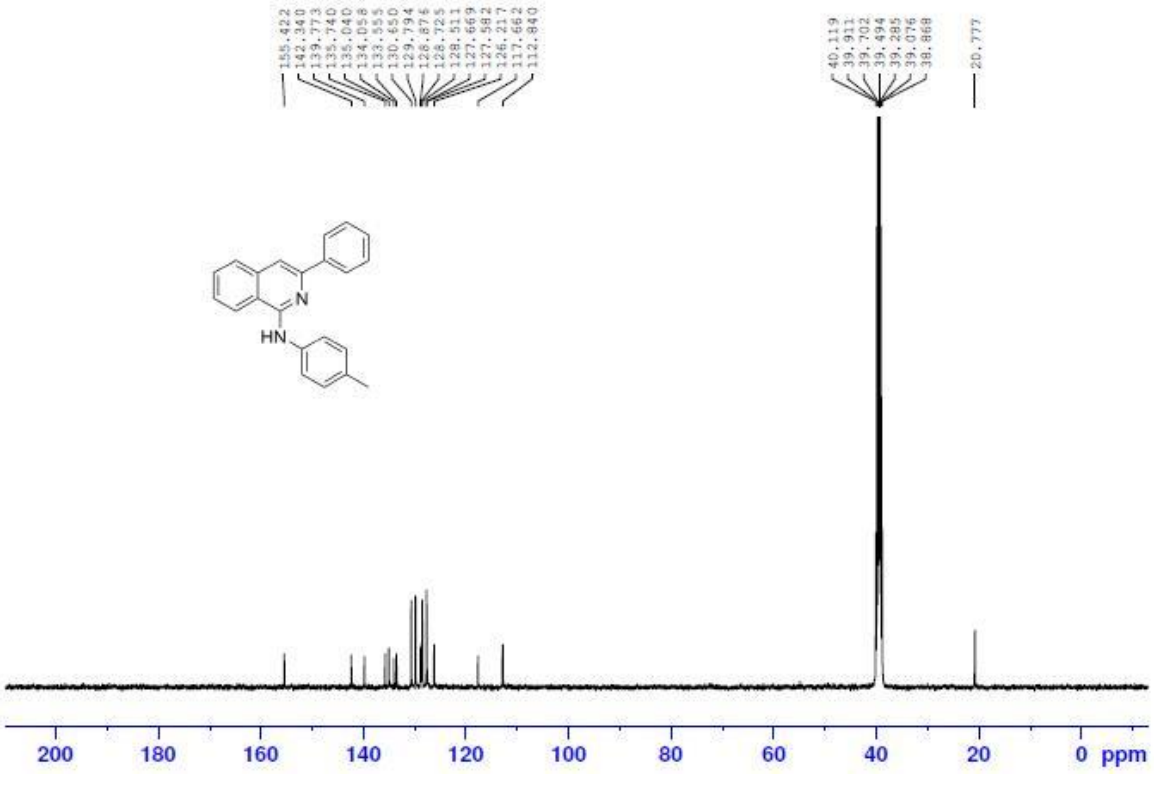

^{1}H NMR Spectrum of **5e**

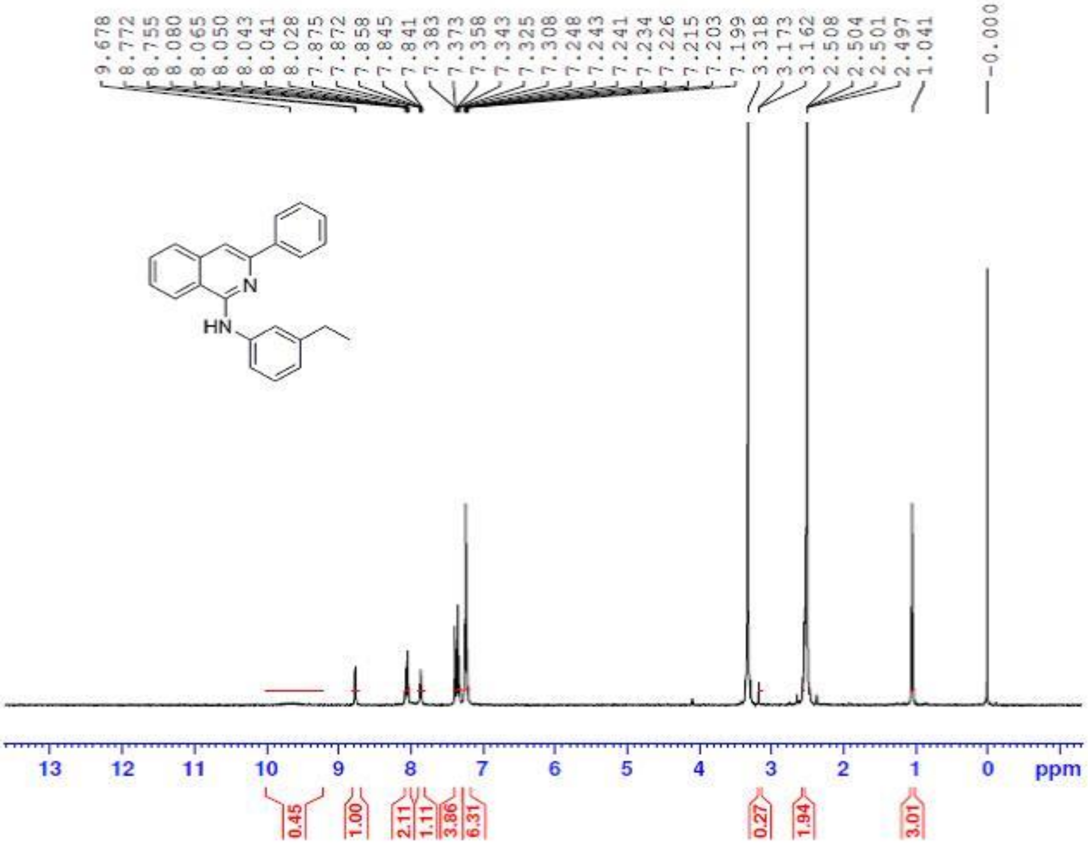

^{13}C NMR Spectrum of **5e**

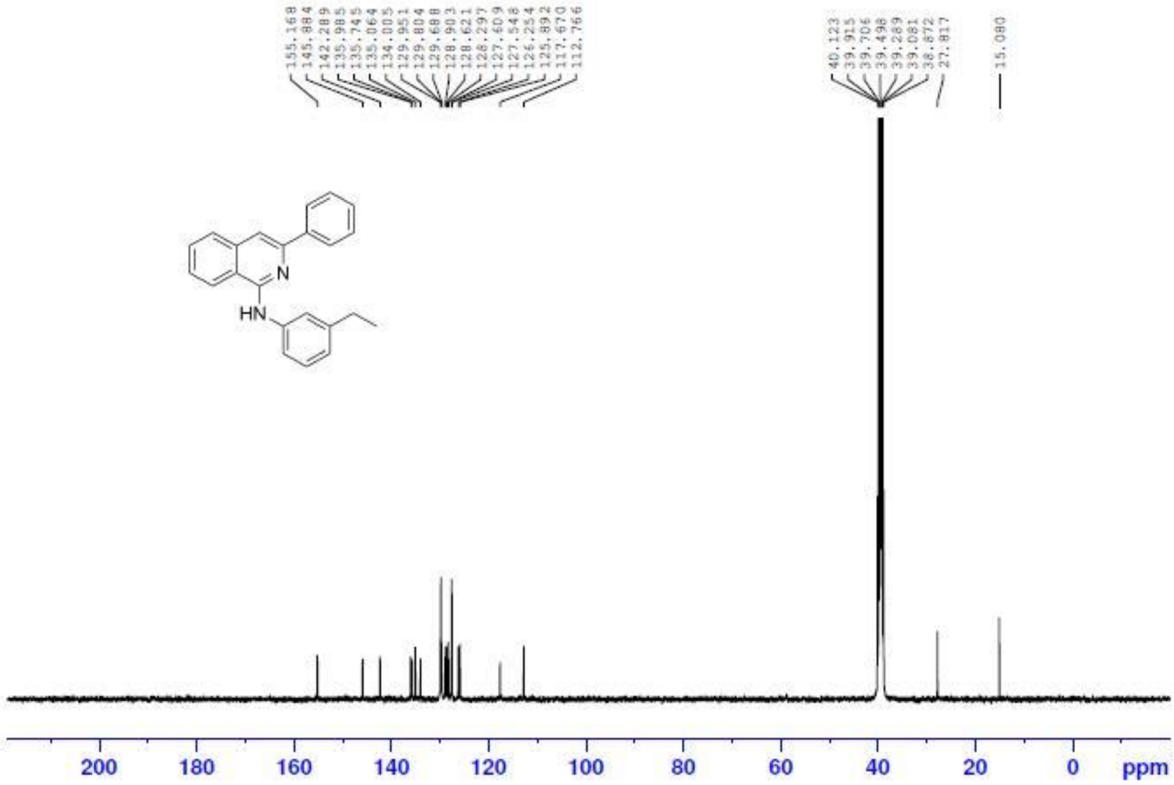

^{1}H NMR Spectrum of **5f**

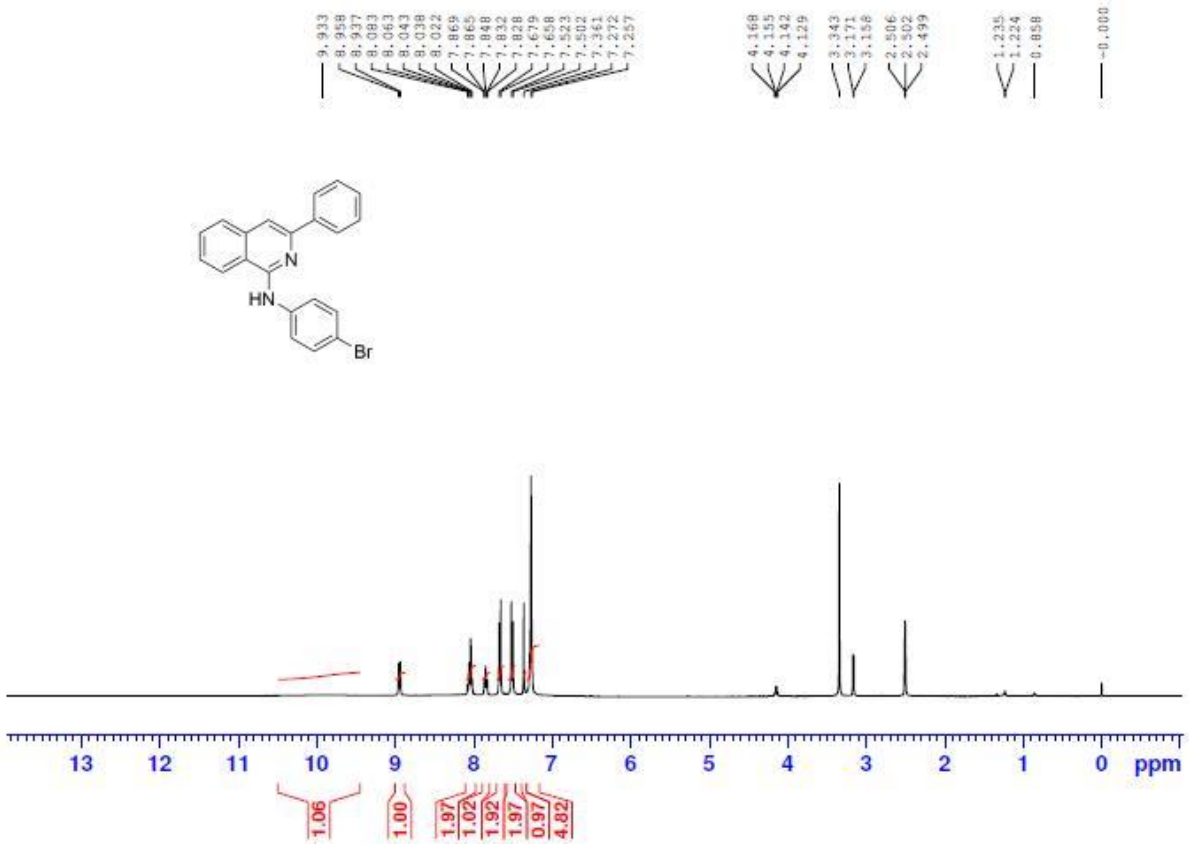

^{13}C NMR Spectrum of **5f**

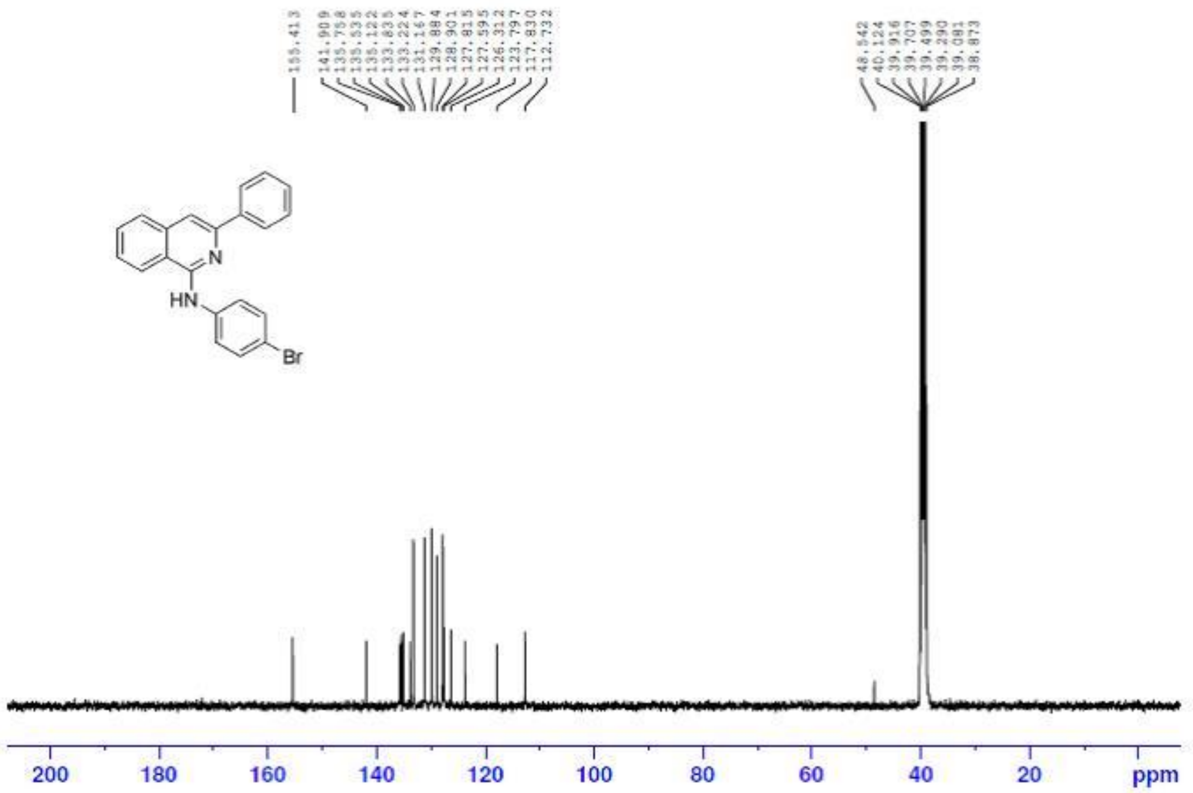

^{1}H NMR Spectrum of **5g**

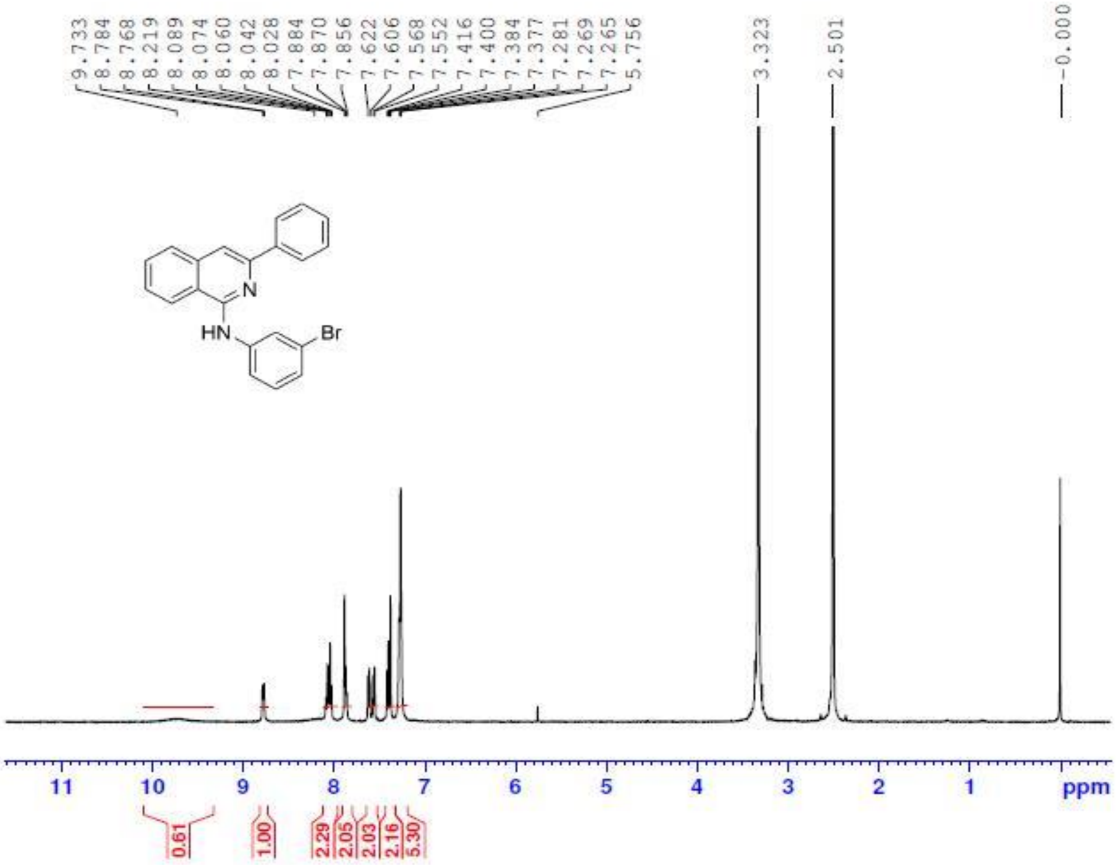

^{13}C NMR Spectrum of **5g**

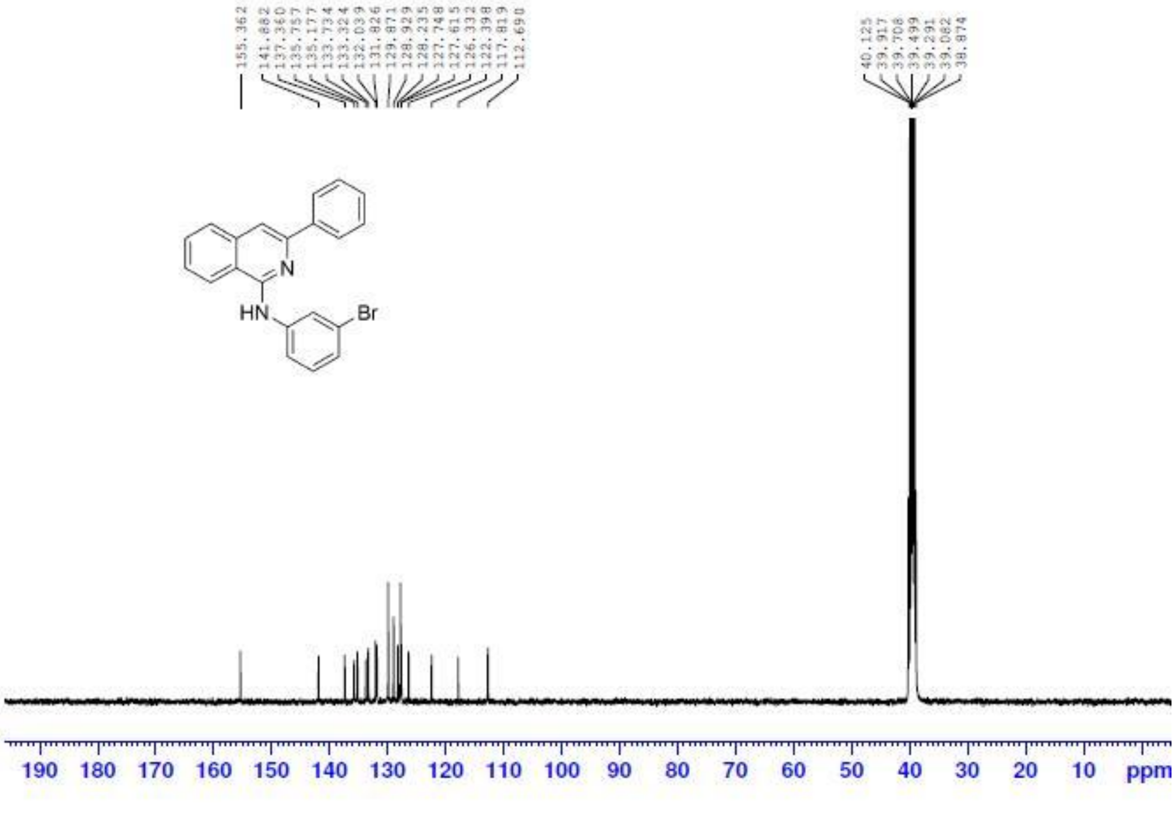

^{1}H NMR Spectrum of **5h**

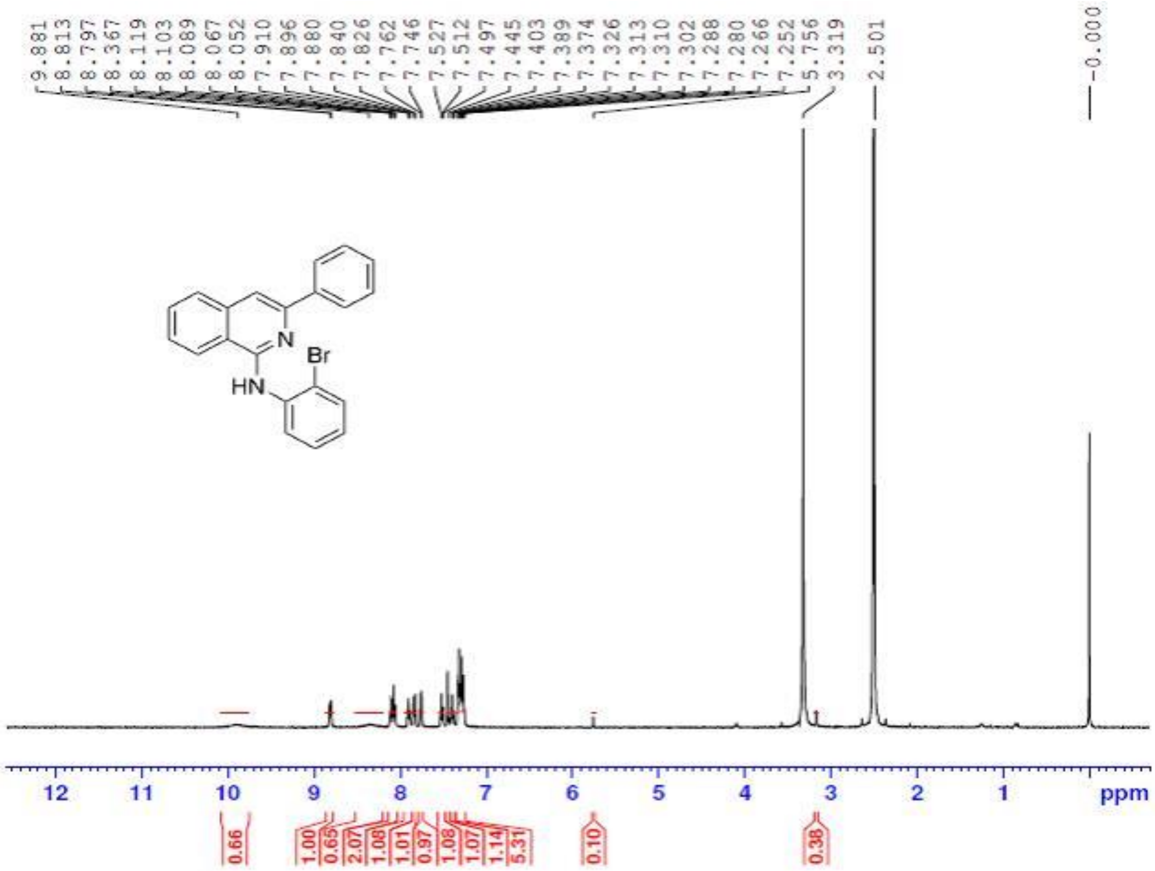

^{13}C NMR Spectrum of **5h**

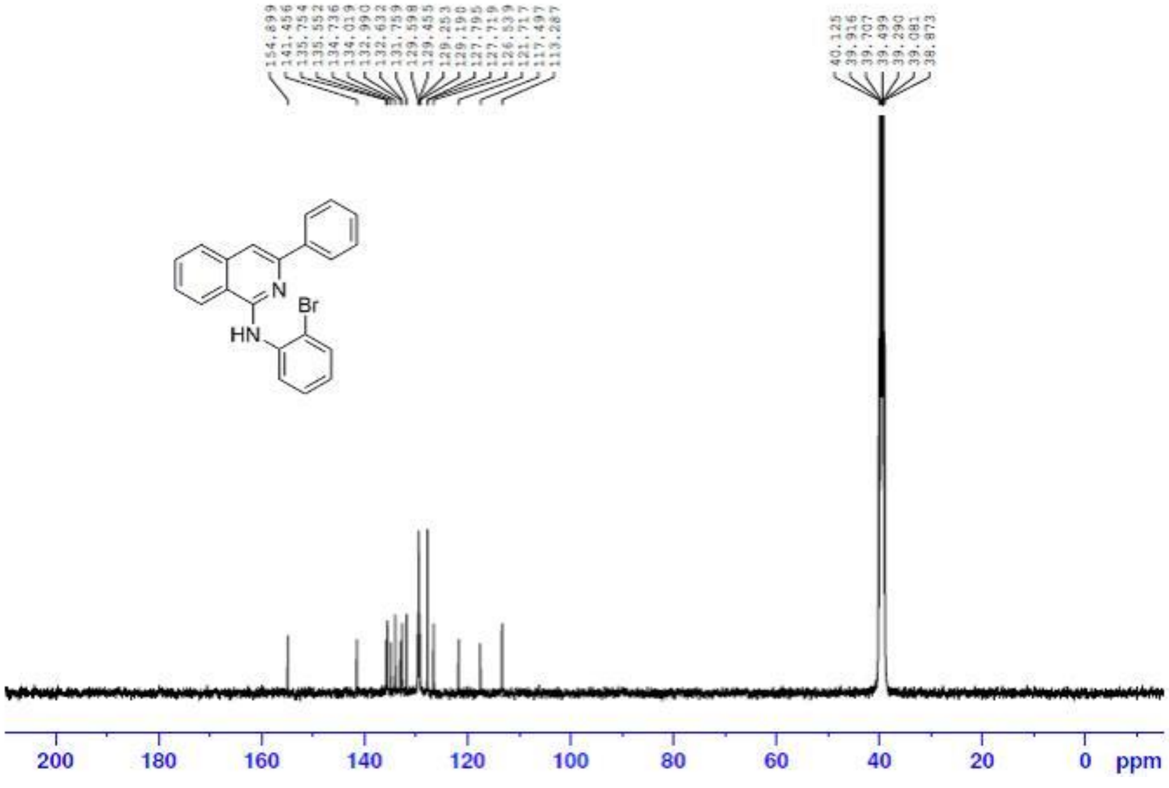

^{1}H NMR Spectrum of **5i**

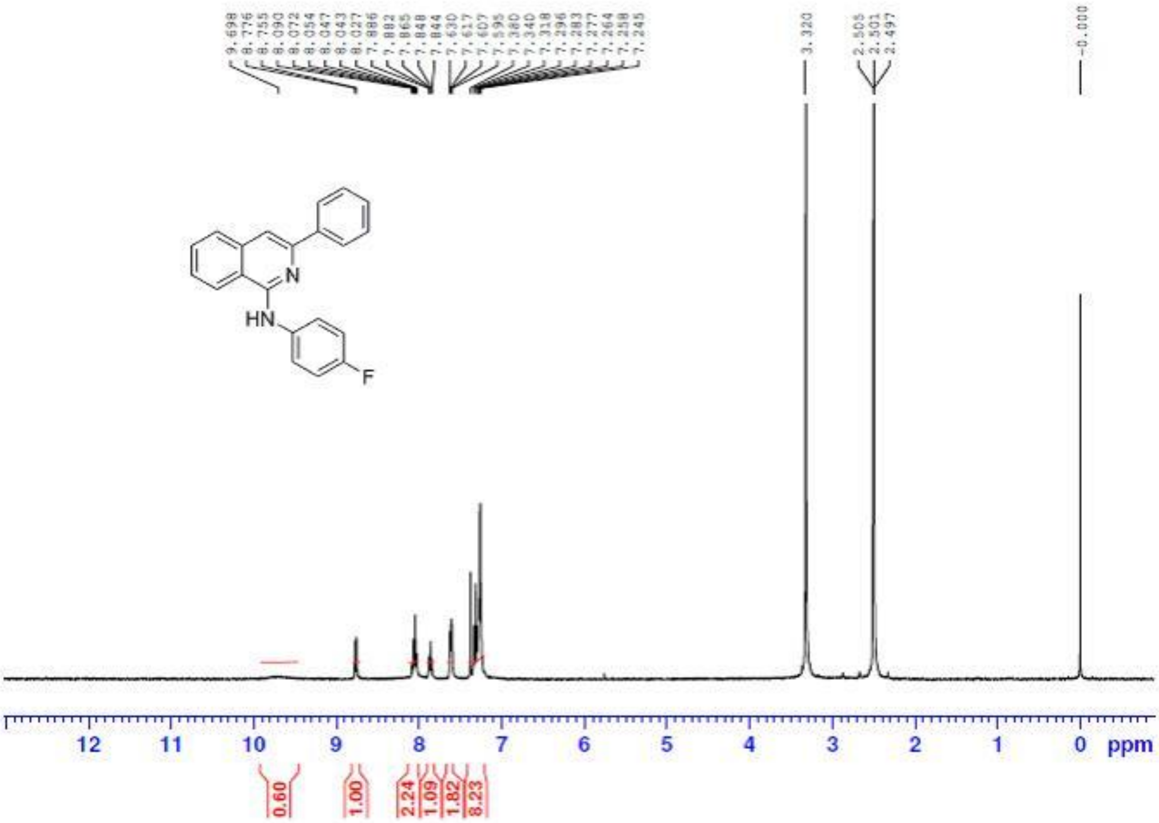

^{13}C NMR Spectrum of **5i**

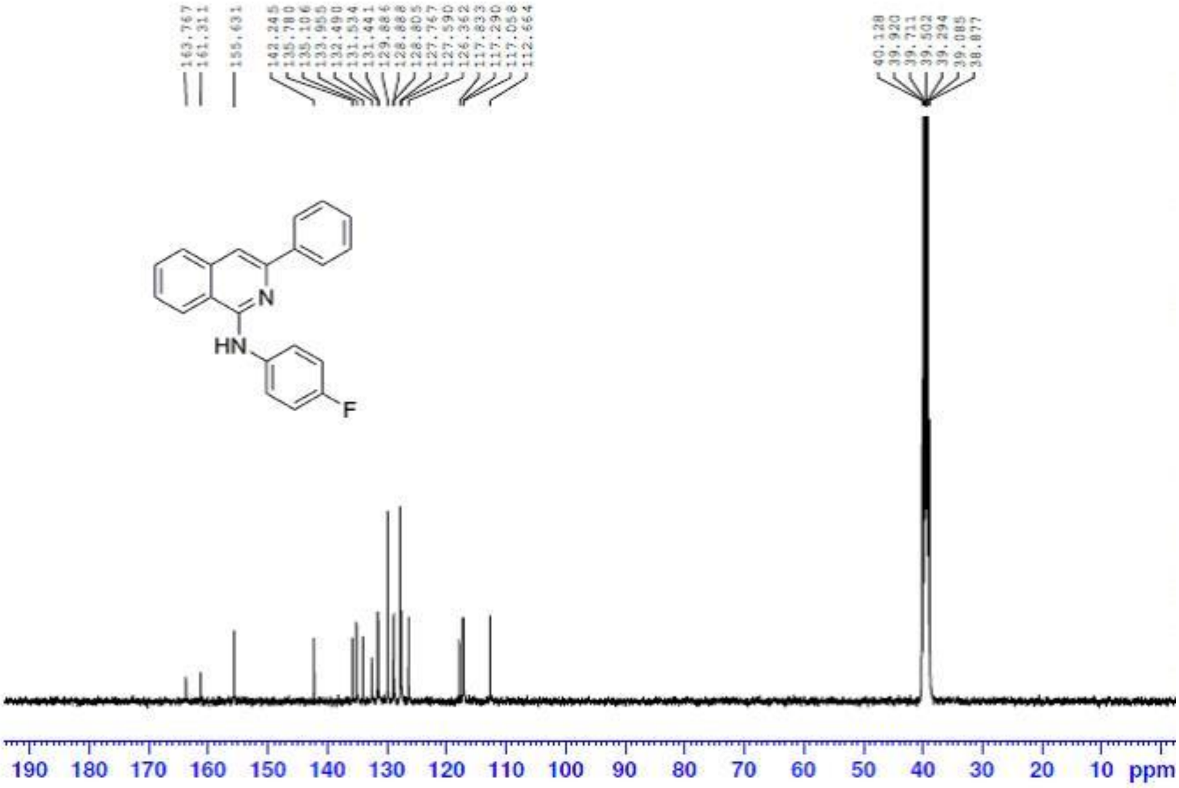

^{19}F-NMR Spectrum of **5i**

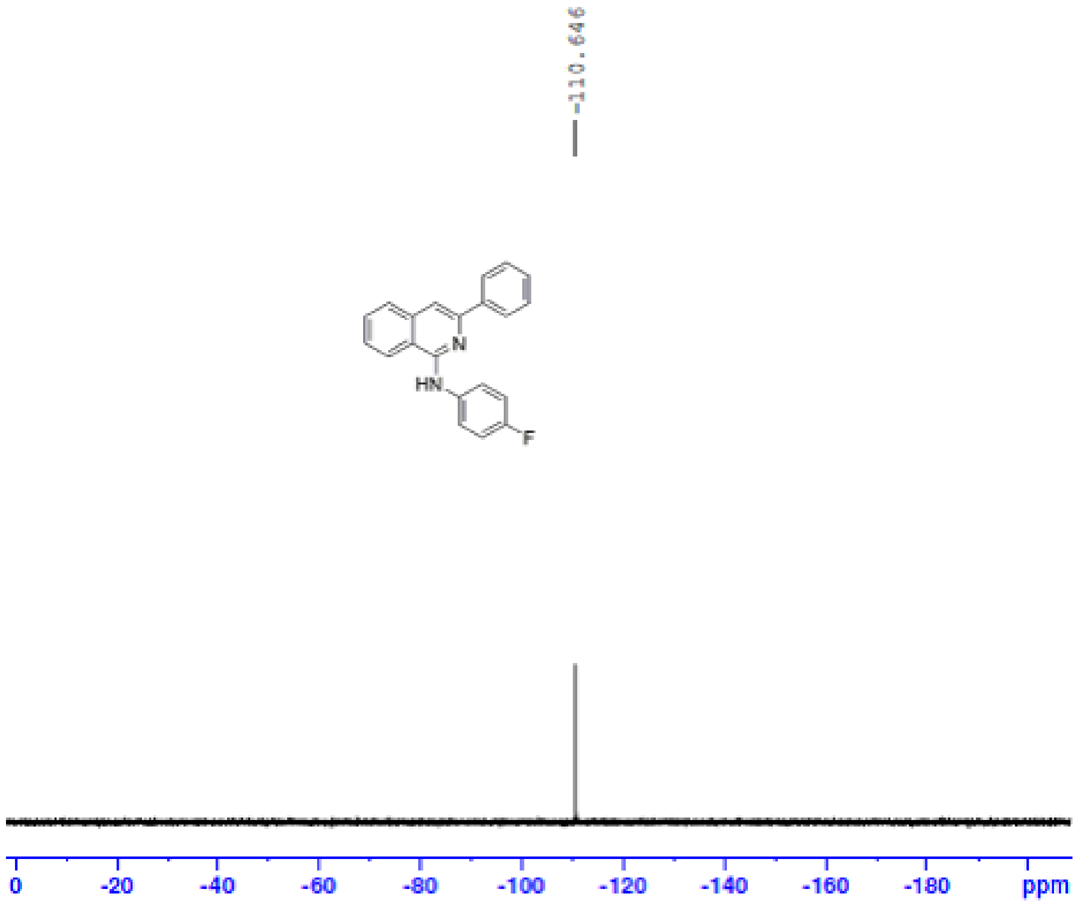

^{1}H NMR Spectrum of **5j**

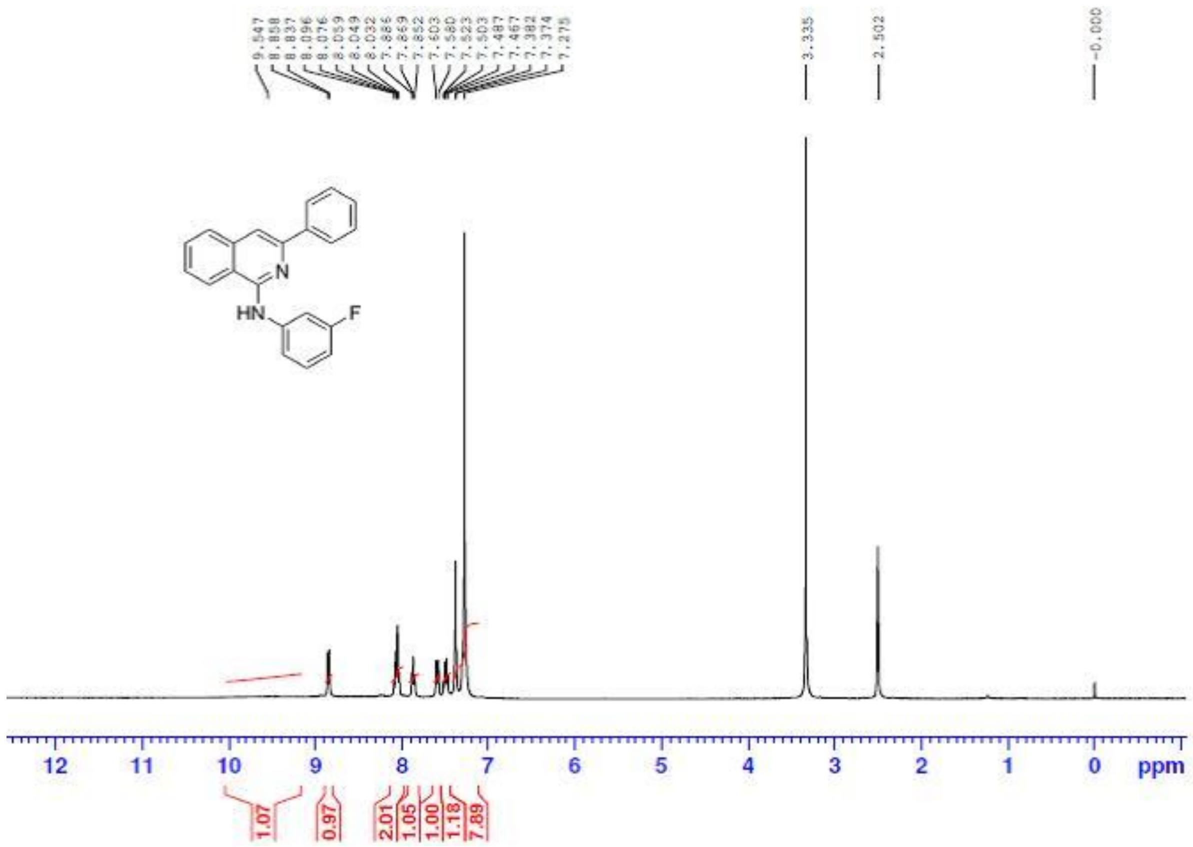

^{13}C NMR Spectrum of **5j**

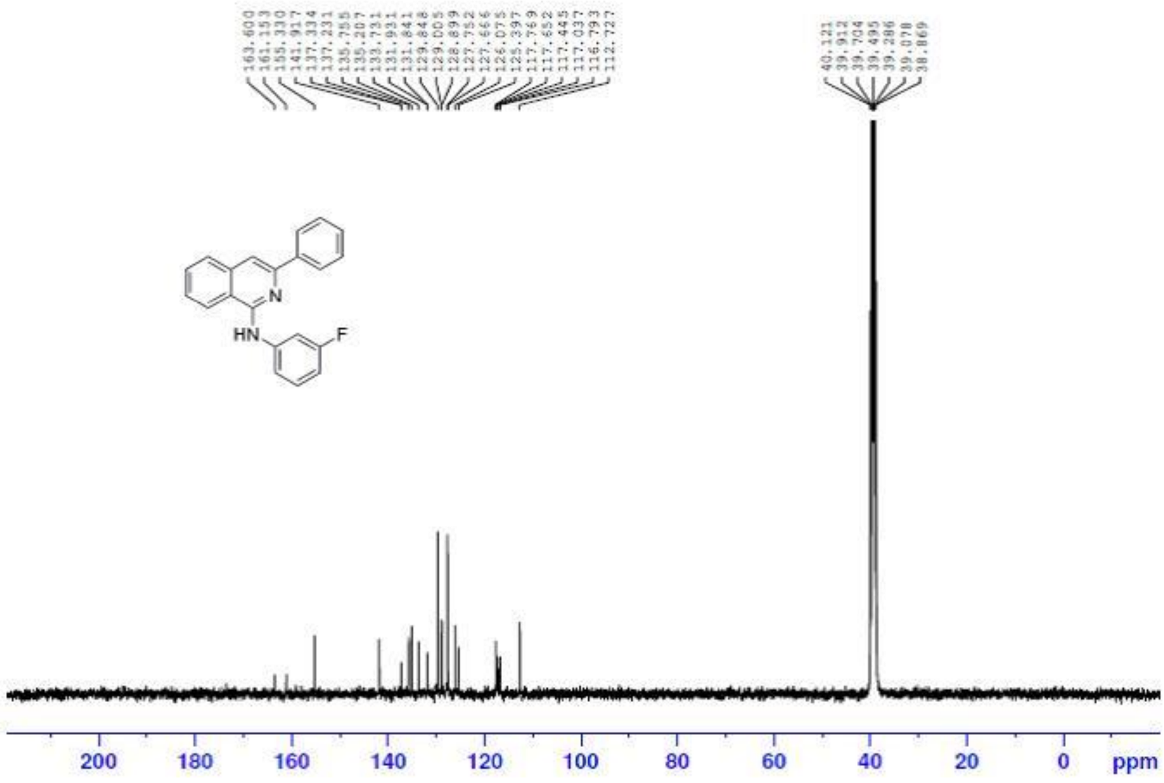

^{19}F-NMR Spectrum of **5j**

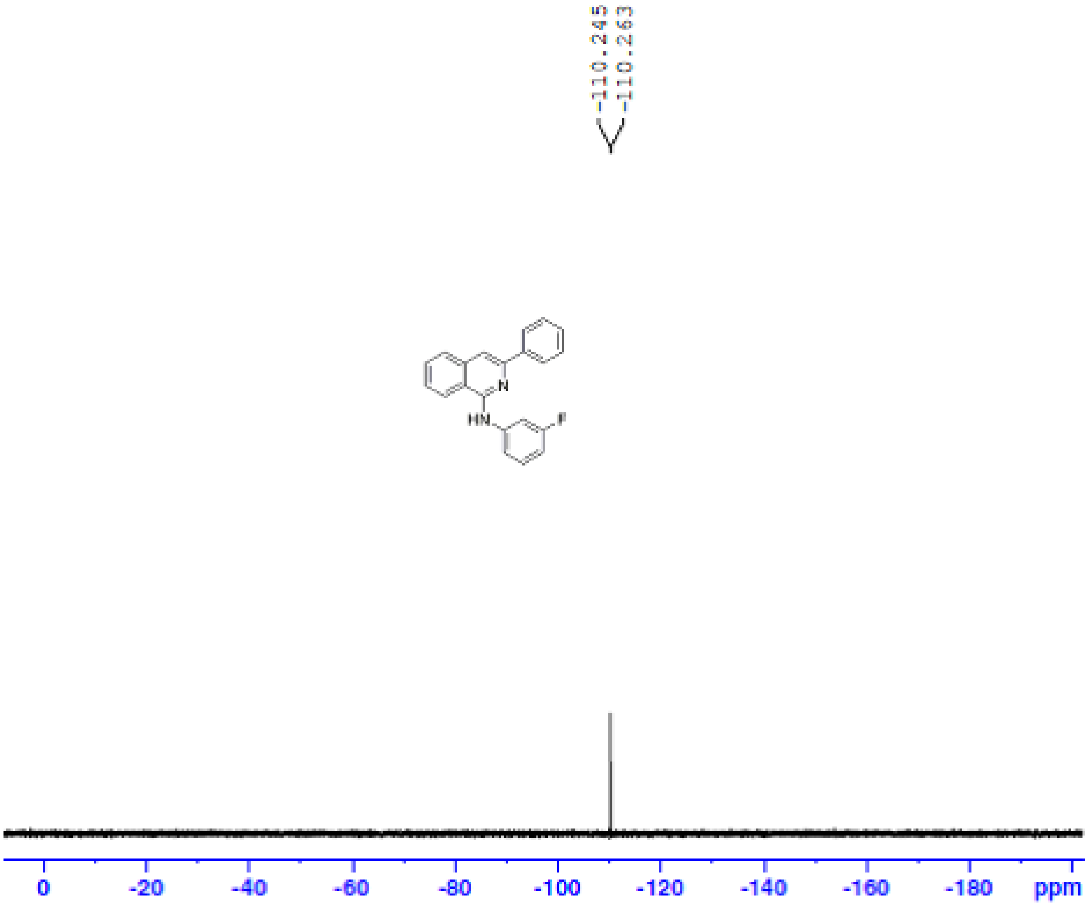

^{1}H NMR Spectrum of **5k**

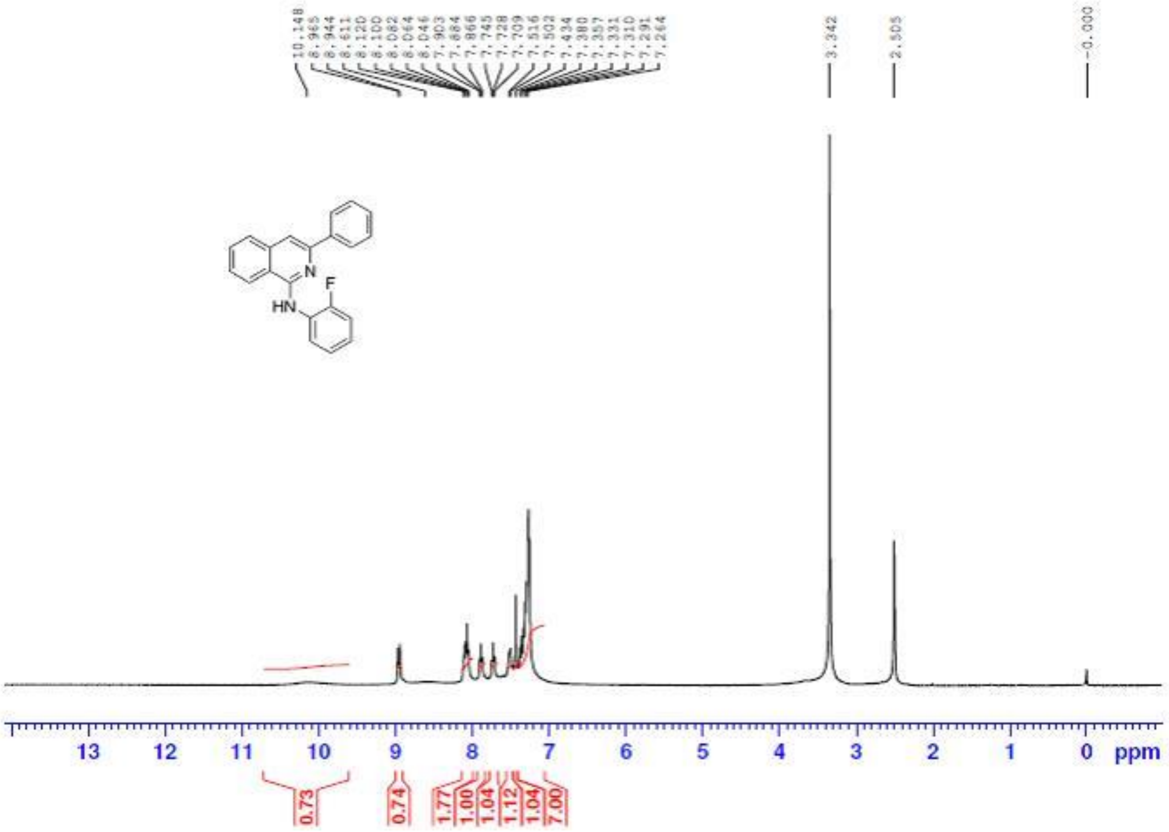

^{13}C NMR Spectrum of **5k**

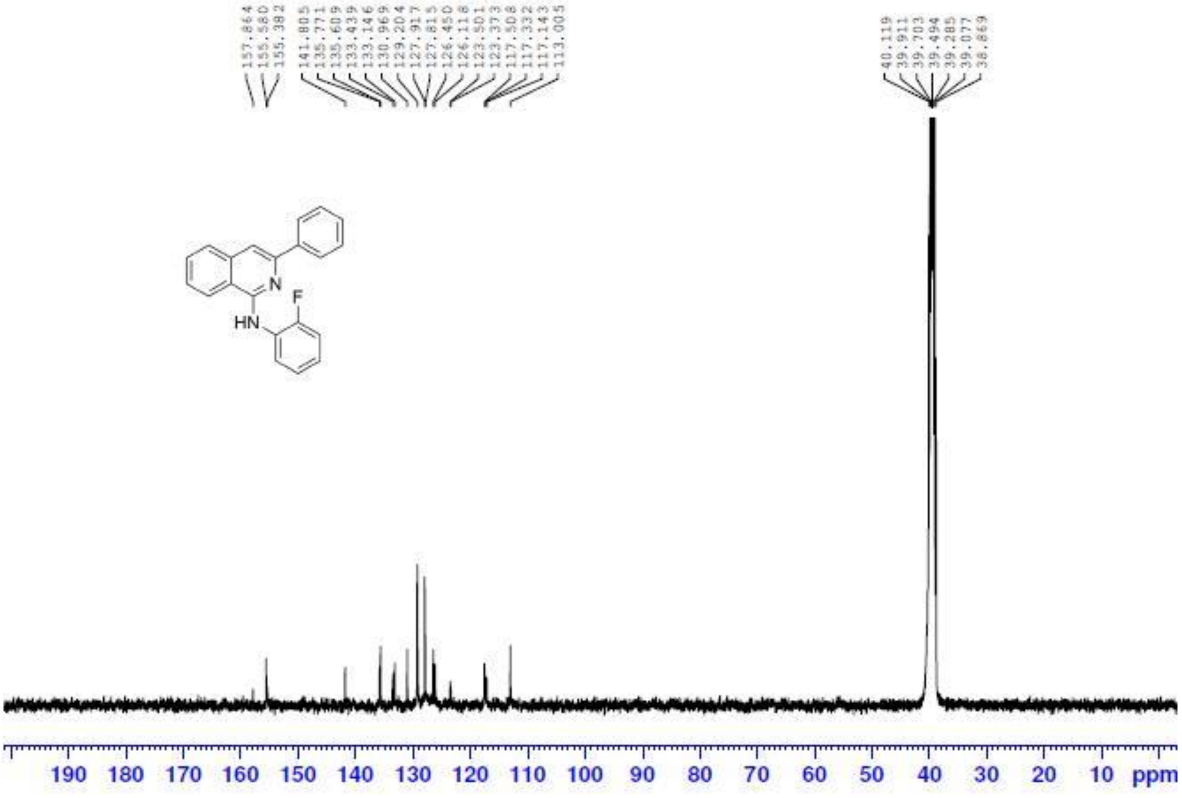

^{19}F-NMR Spectrum of **5k**

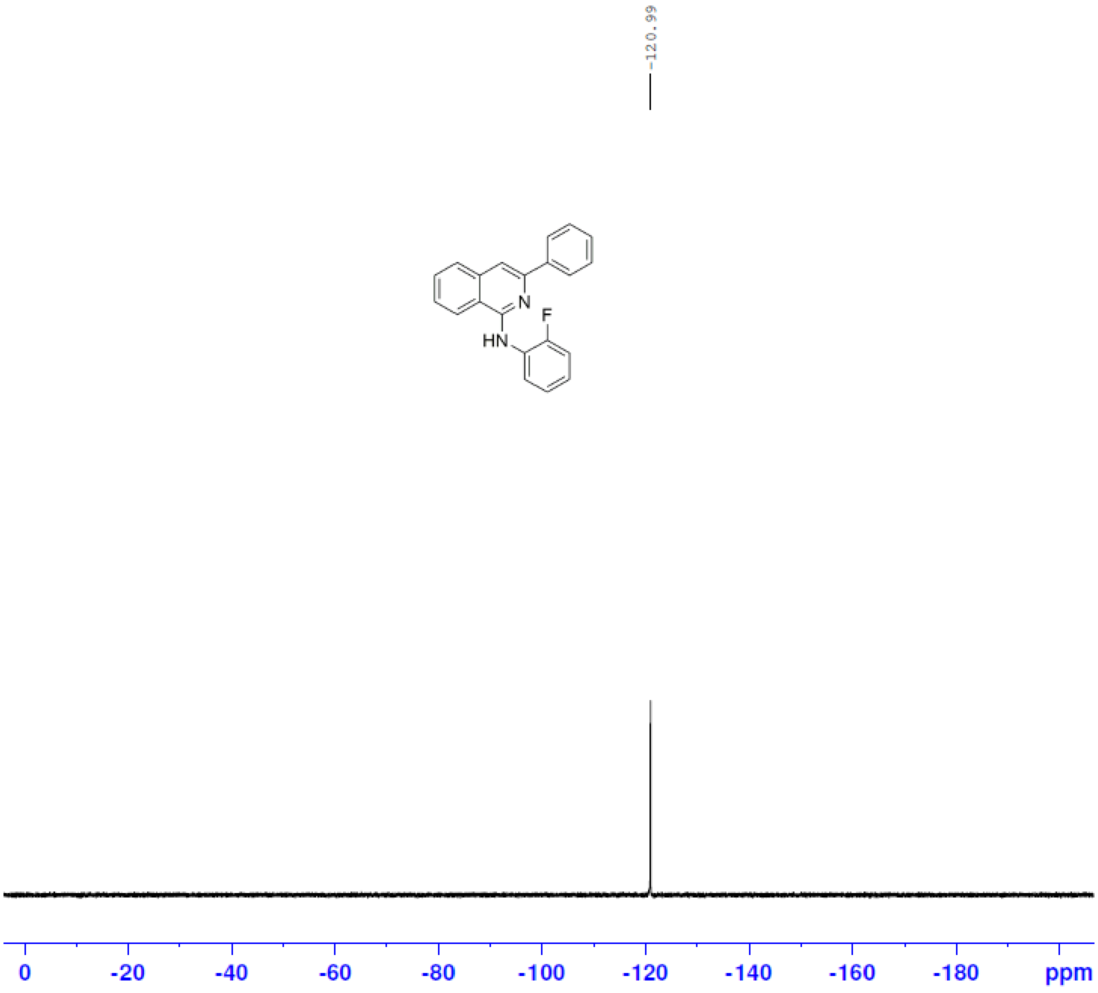

^{1}H NMR Spectrum of **5l**

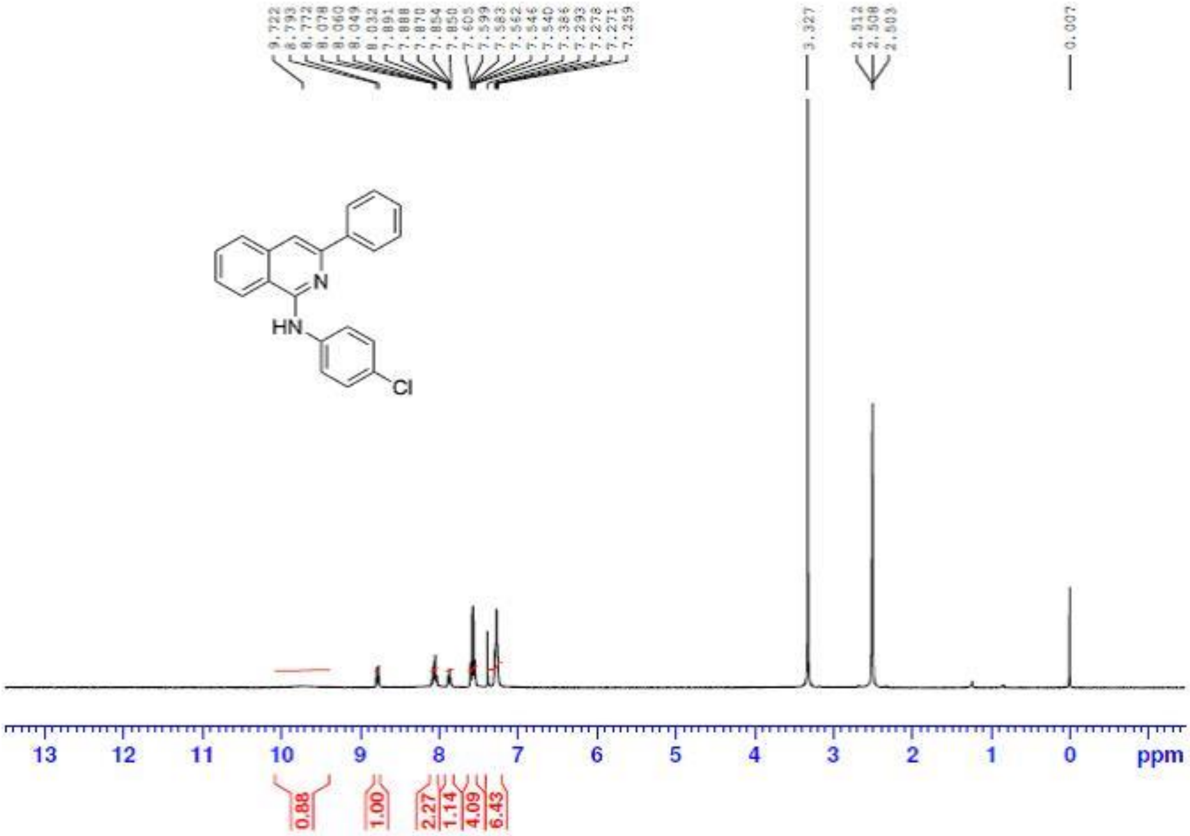

^{13}C NMR Spectrum of **5l**

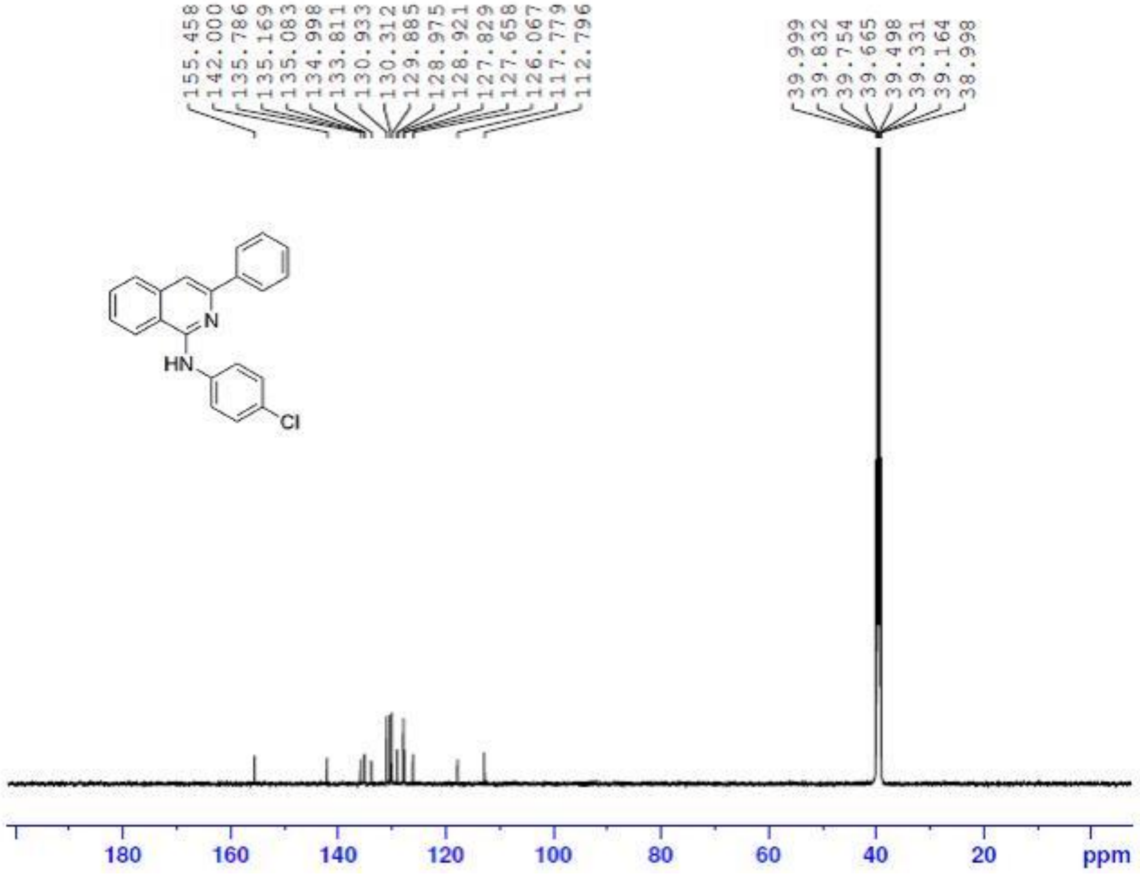

^{1}H NMR Spectrum of **5m**

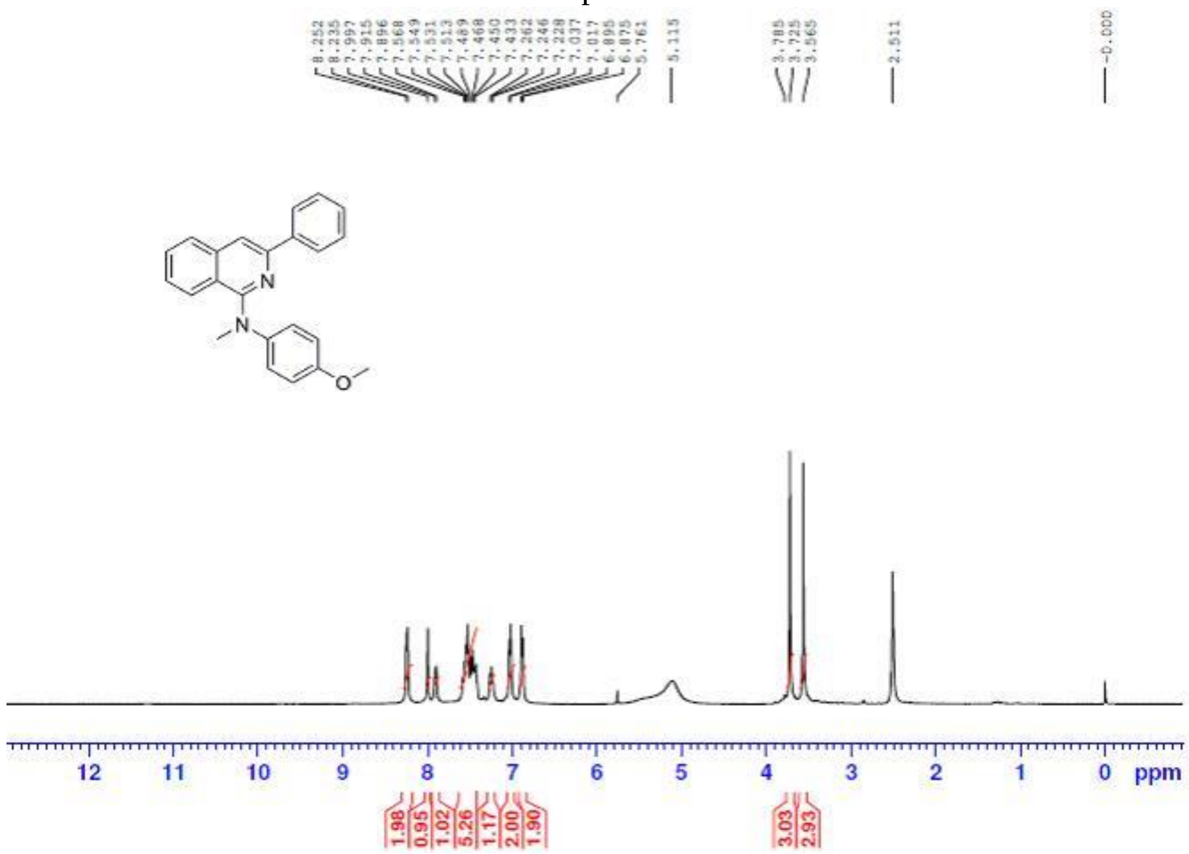

^{13}C NMR Spectrum of **5m**

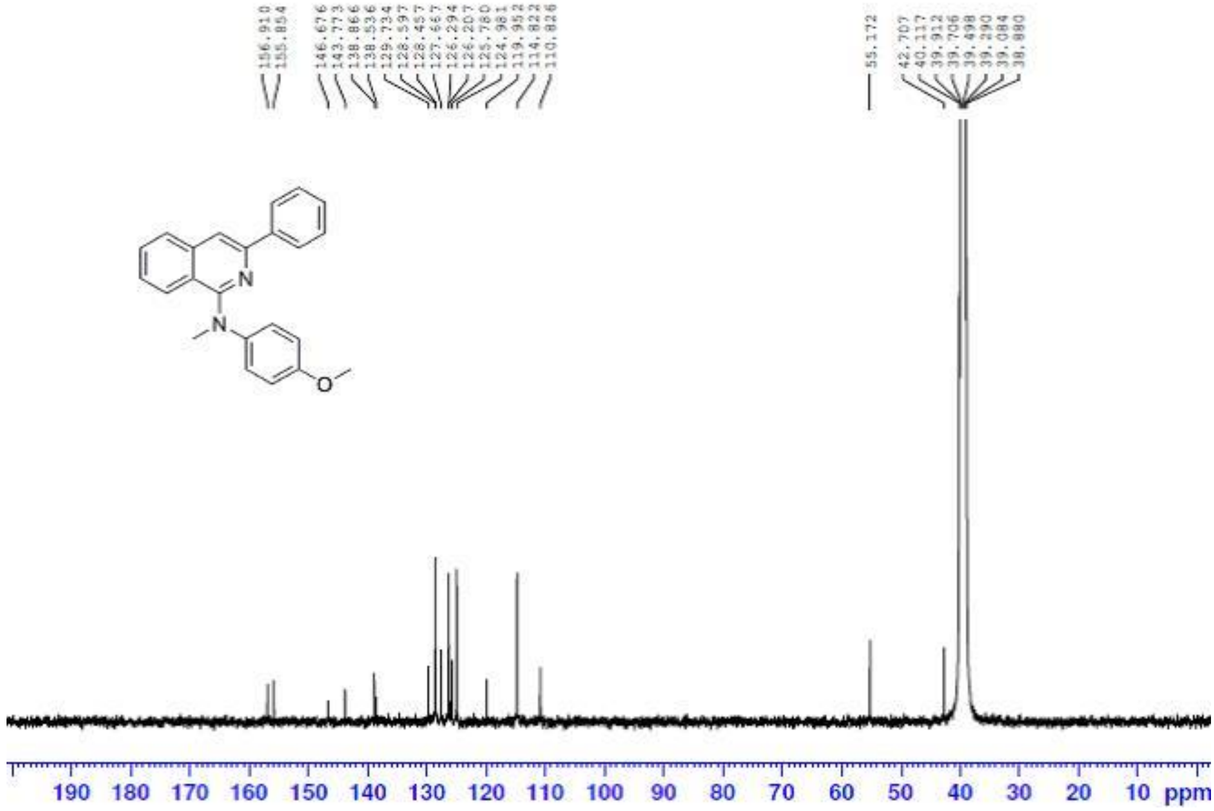

^{1}H NMR Spectrum of **5n**

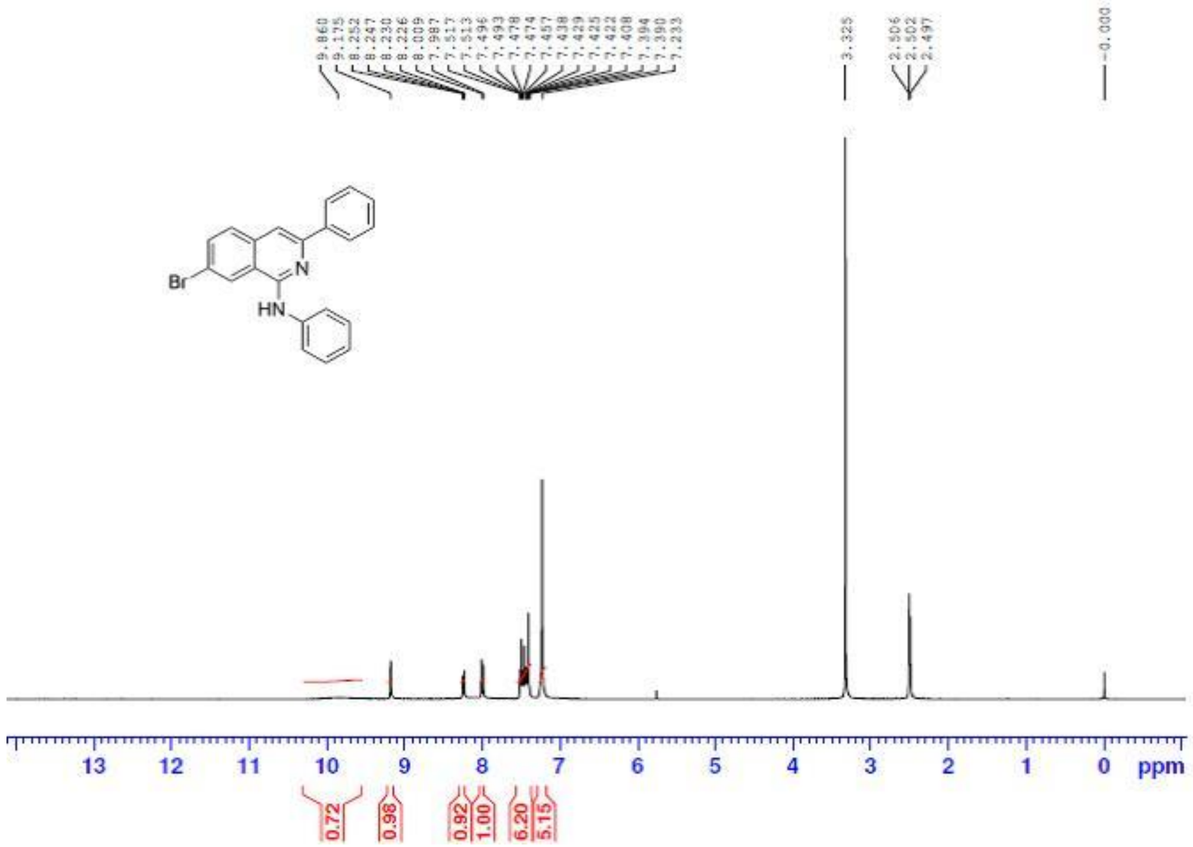

^{13}C NMR Spectrum of **5n**

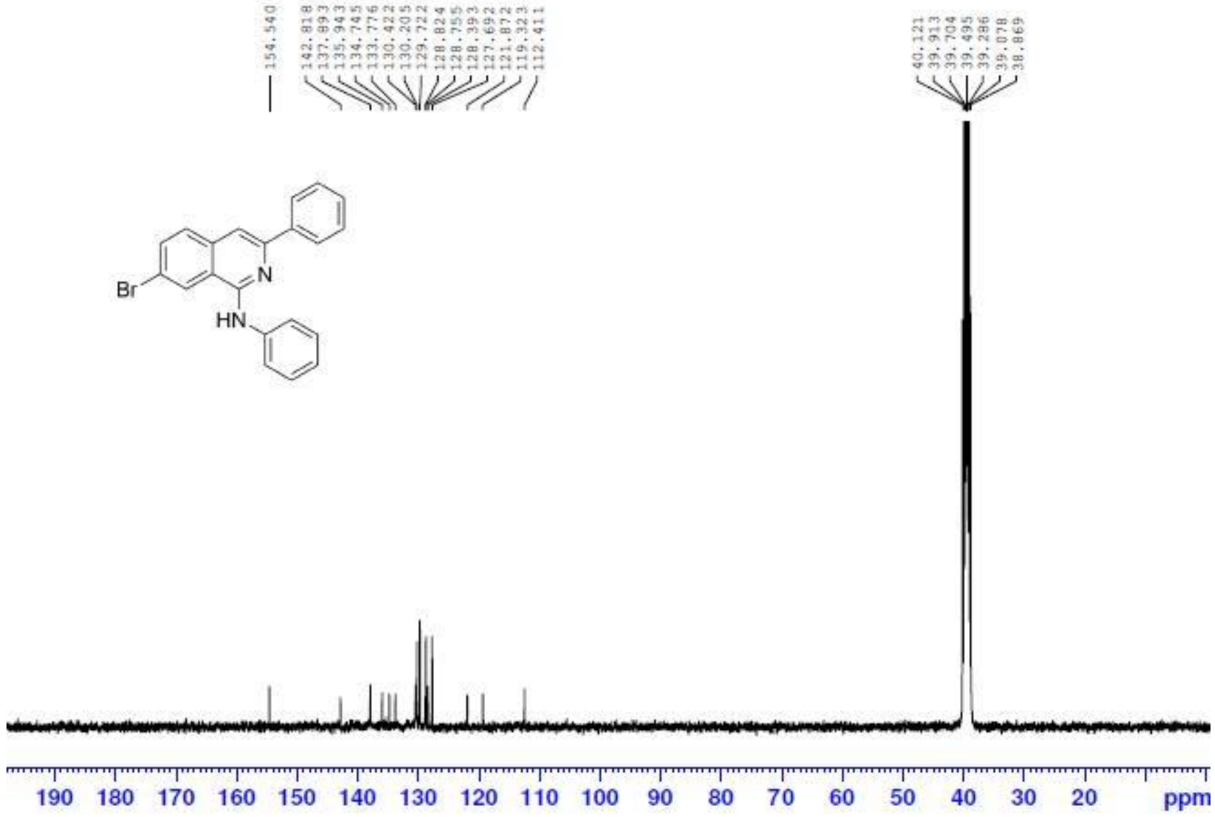

^{1}H NMR Spectrum of **5o**

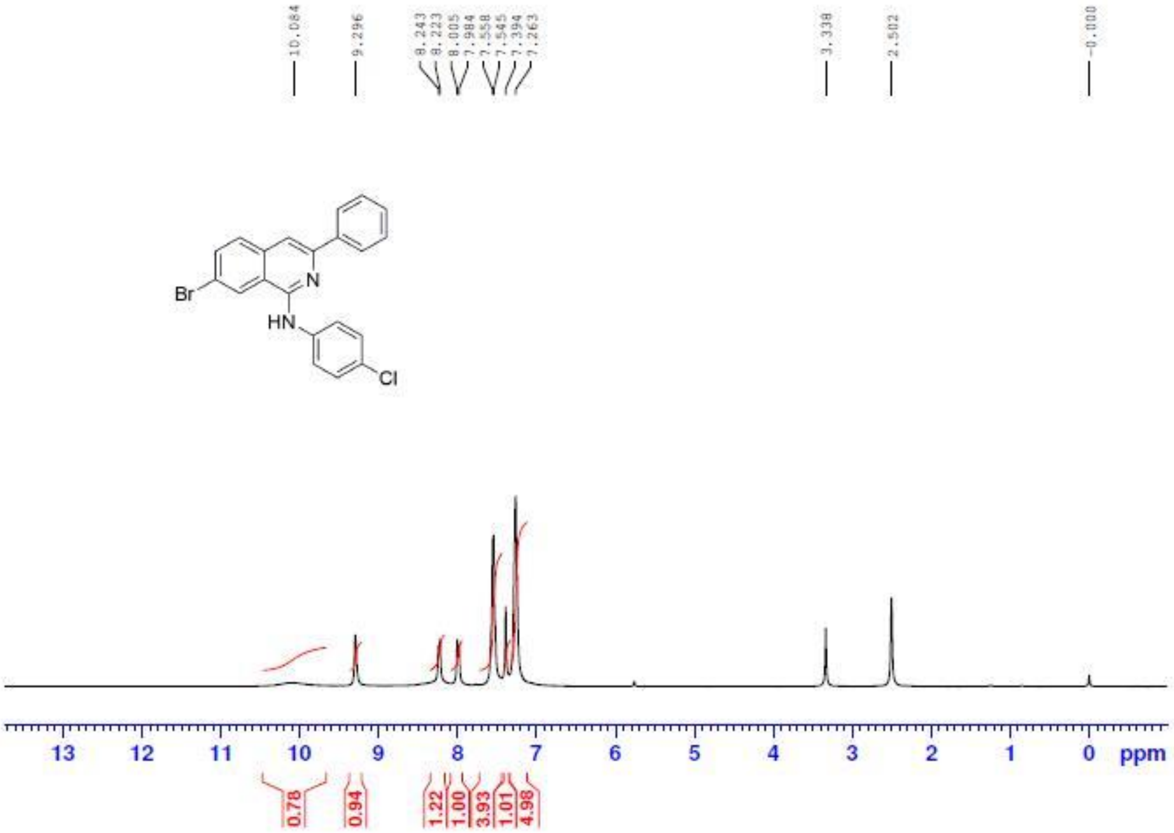

^{13}C NMR Spectrum of **5o**

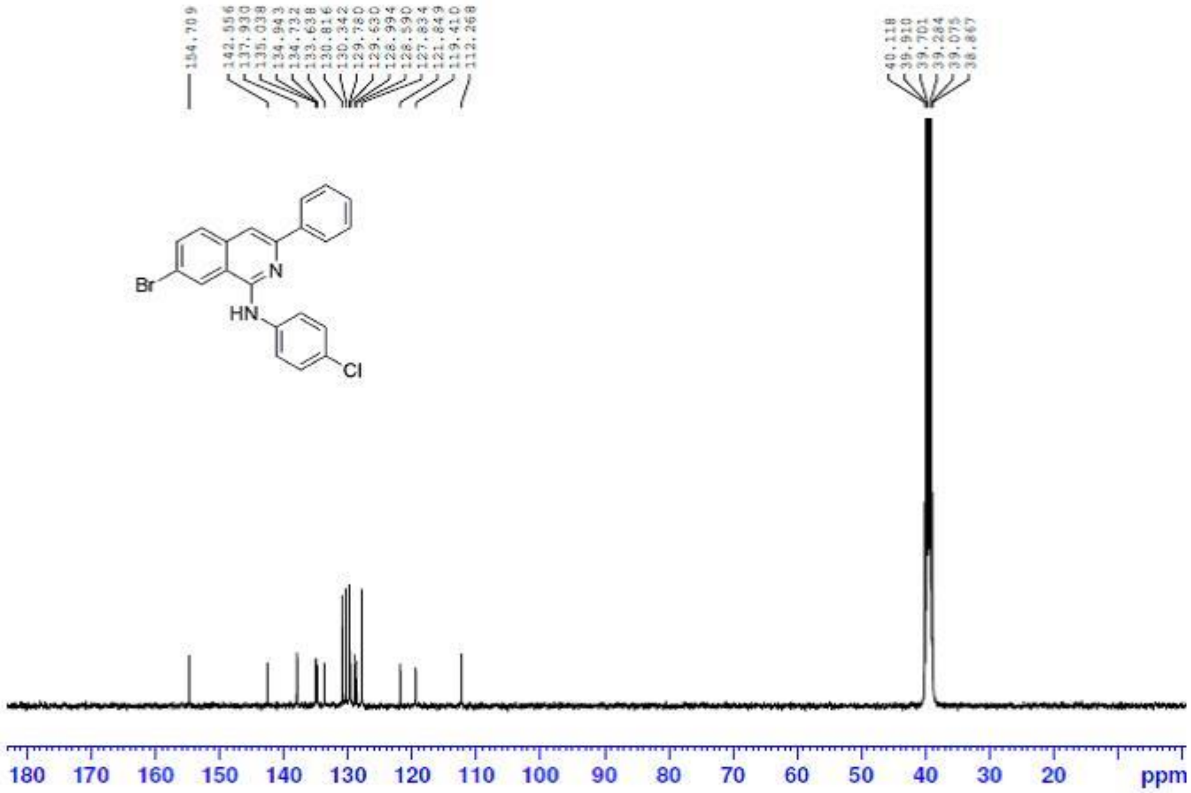

^{1}H NMR Spectrum of **5p**

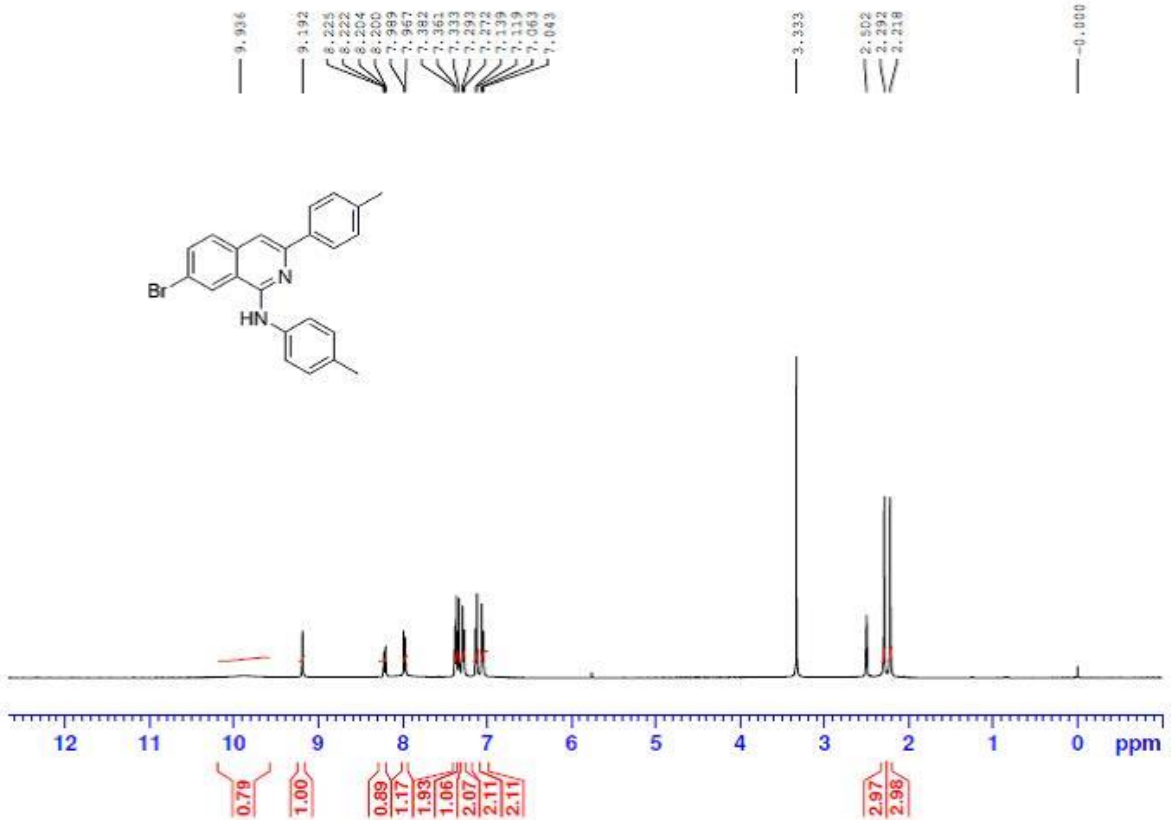

^{13}C NMR Spectrum of **5p**

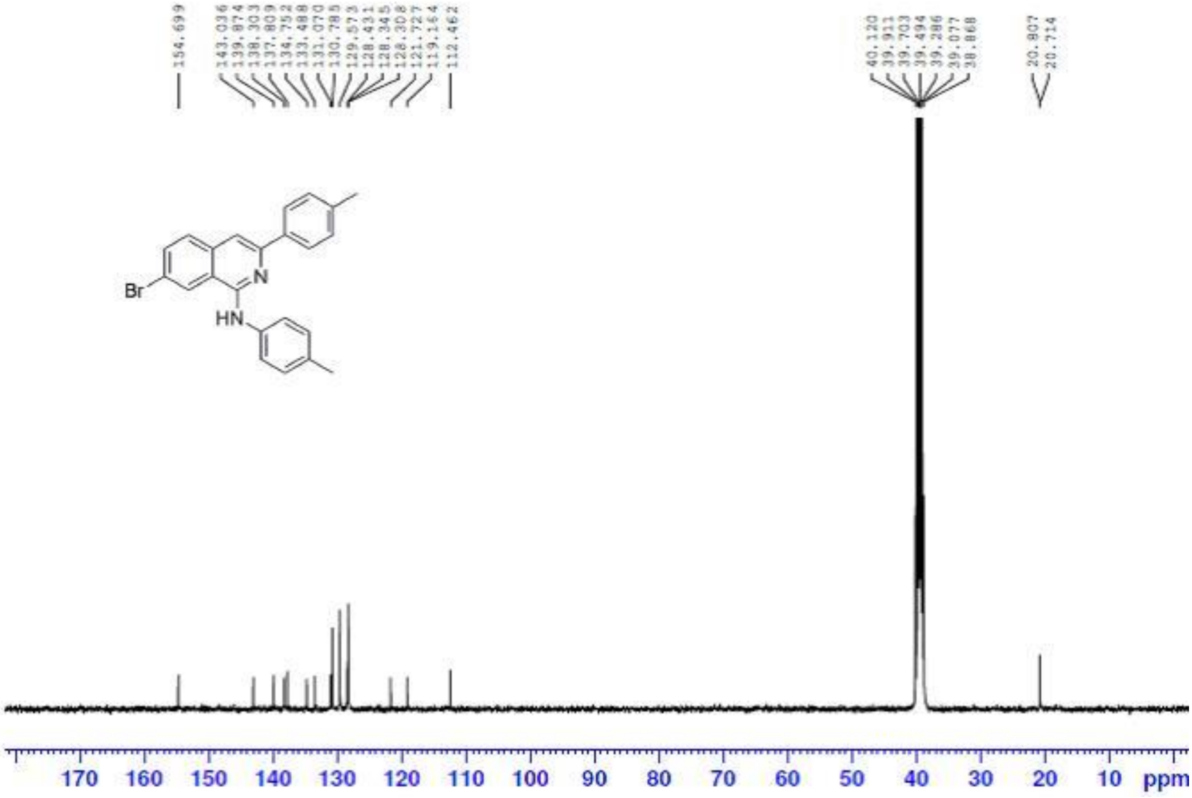

^{1}H NMR Spectrum of **5q**

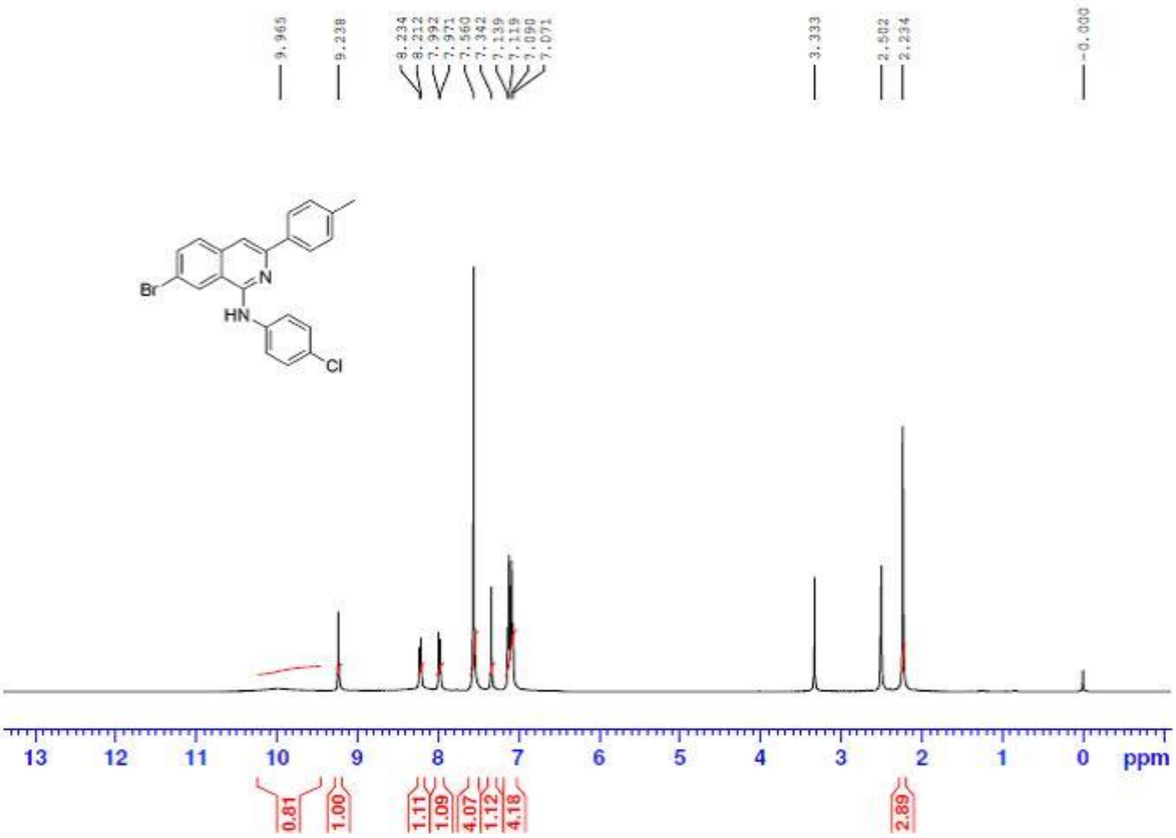

^{13}C NMR Spectrum of **5q**

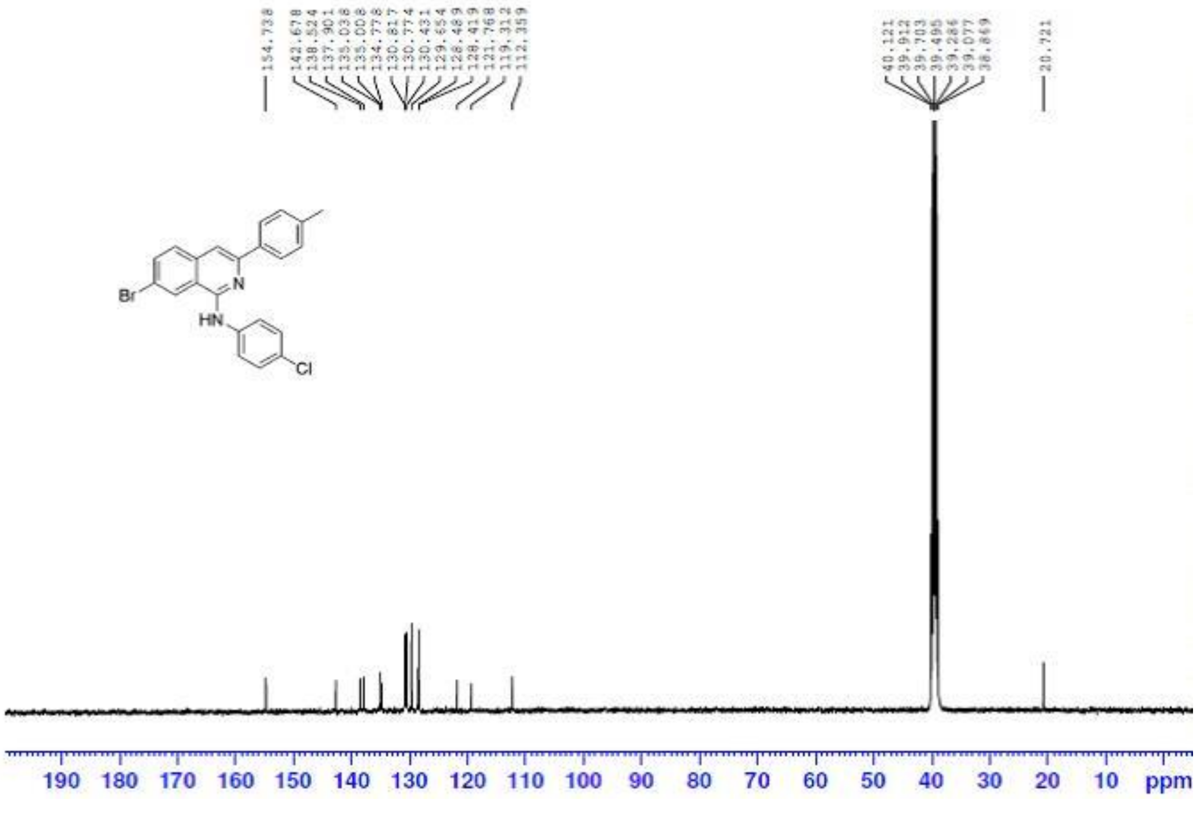

^{1}H NMR Spectrum of **5r**

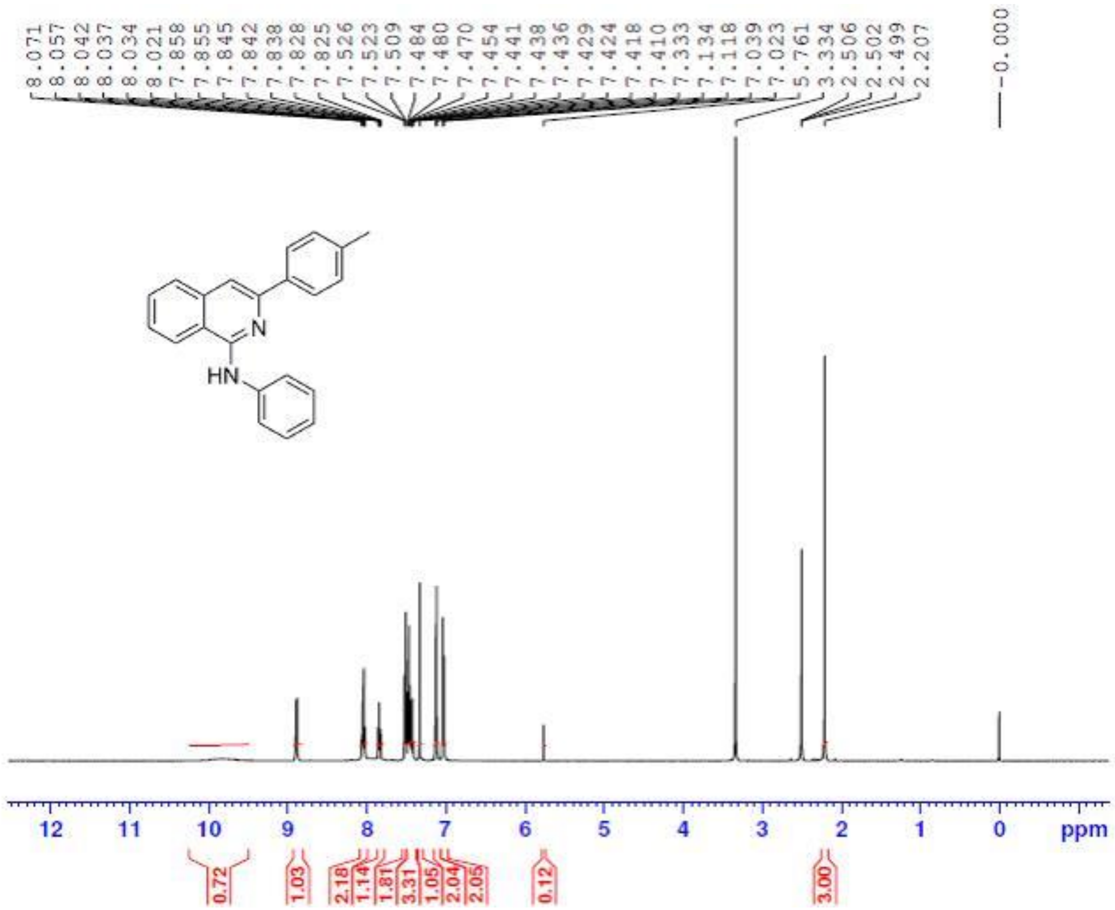

^{13}C NMR Spectrum of **5r**

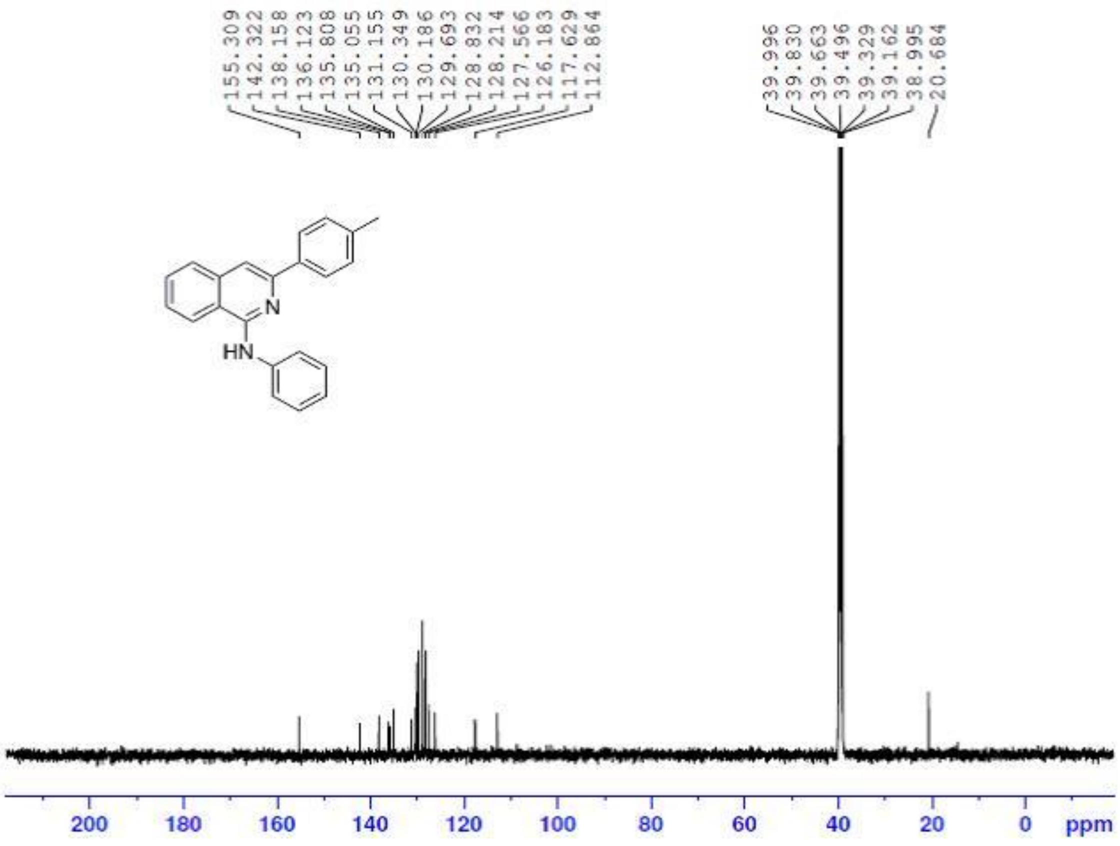

^{1}H NMR Spectrum of **5s**

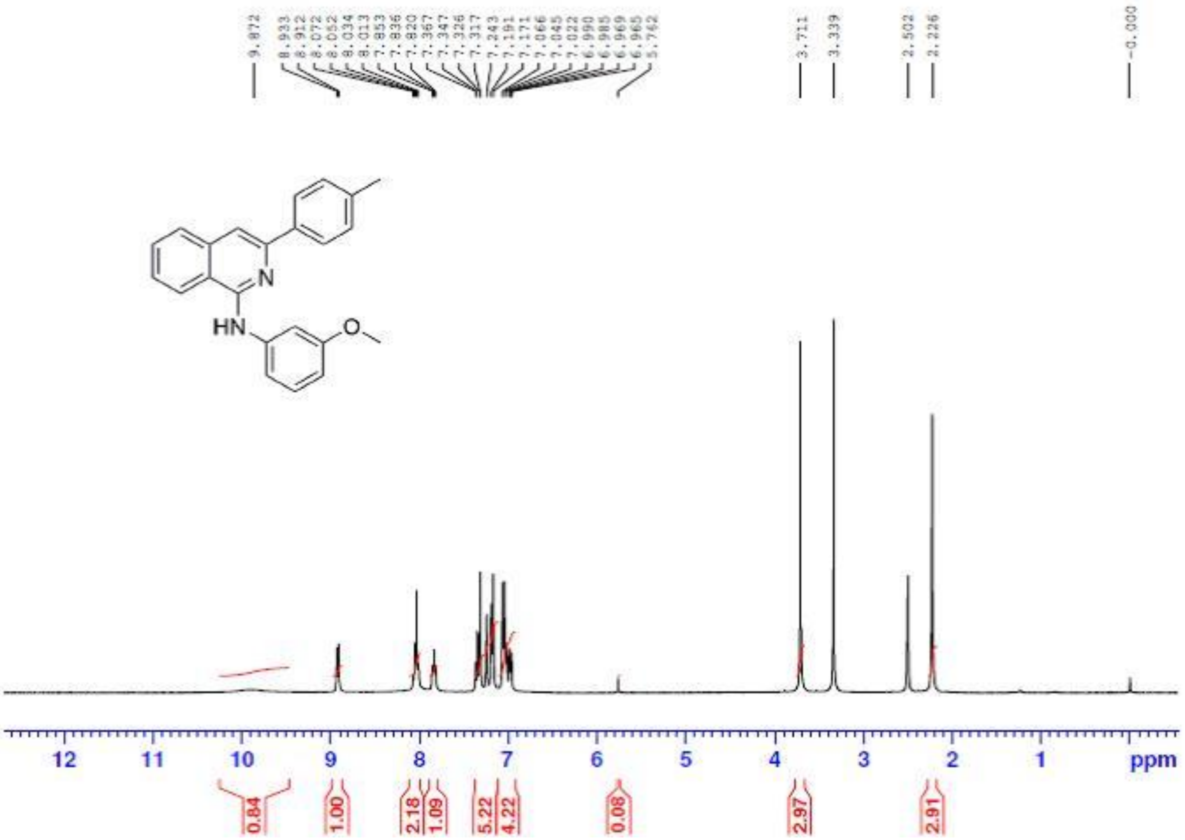

^{13}C NMR Spectrum of **5s**

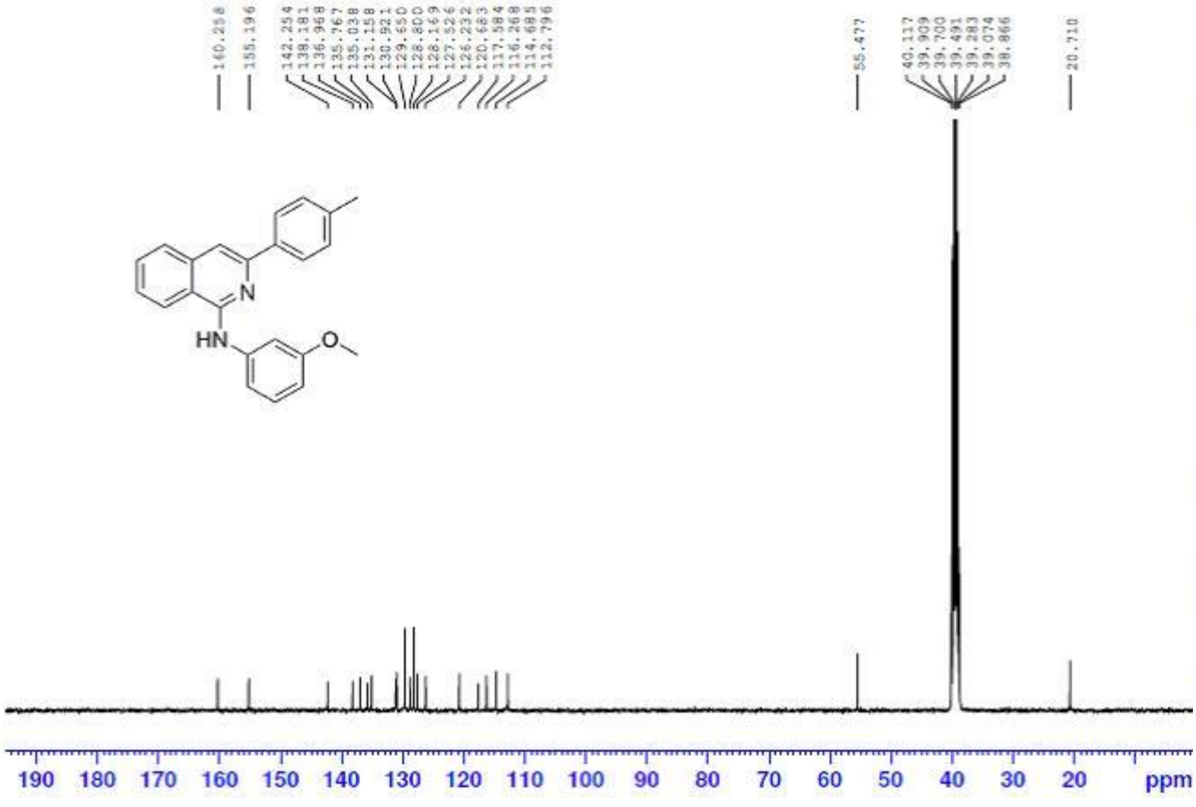

^{1}H NMR Spectrum of **5t**

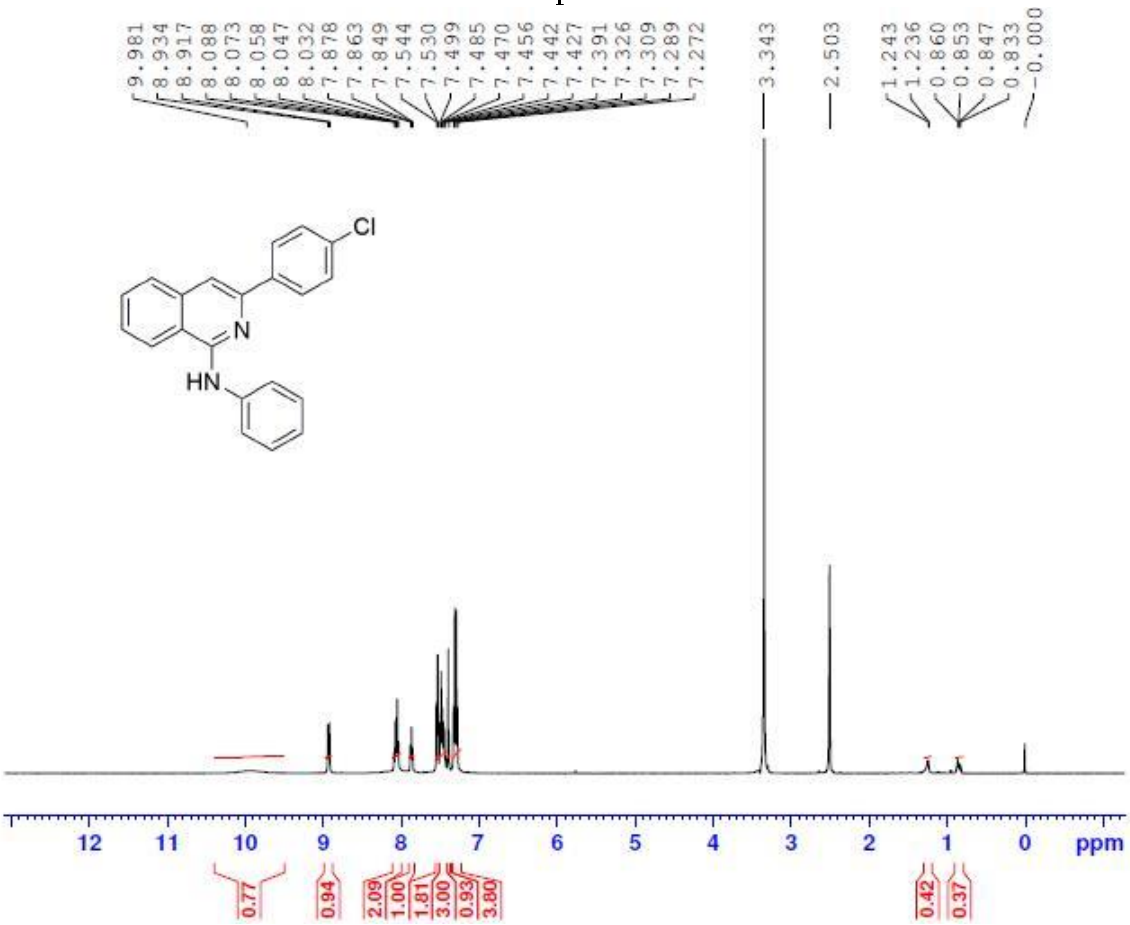

^{13}C NMR Spectrum of **5t**

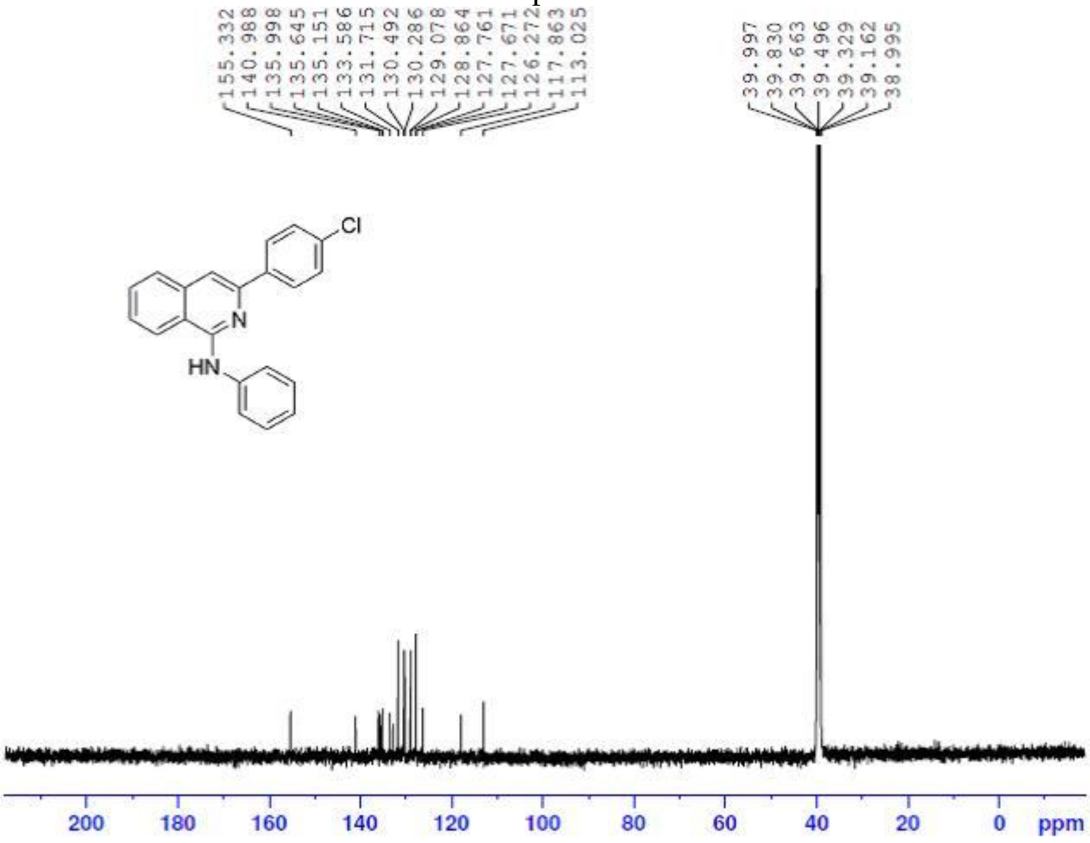

^{1}H NMR Spectrum of **5u**

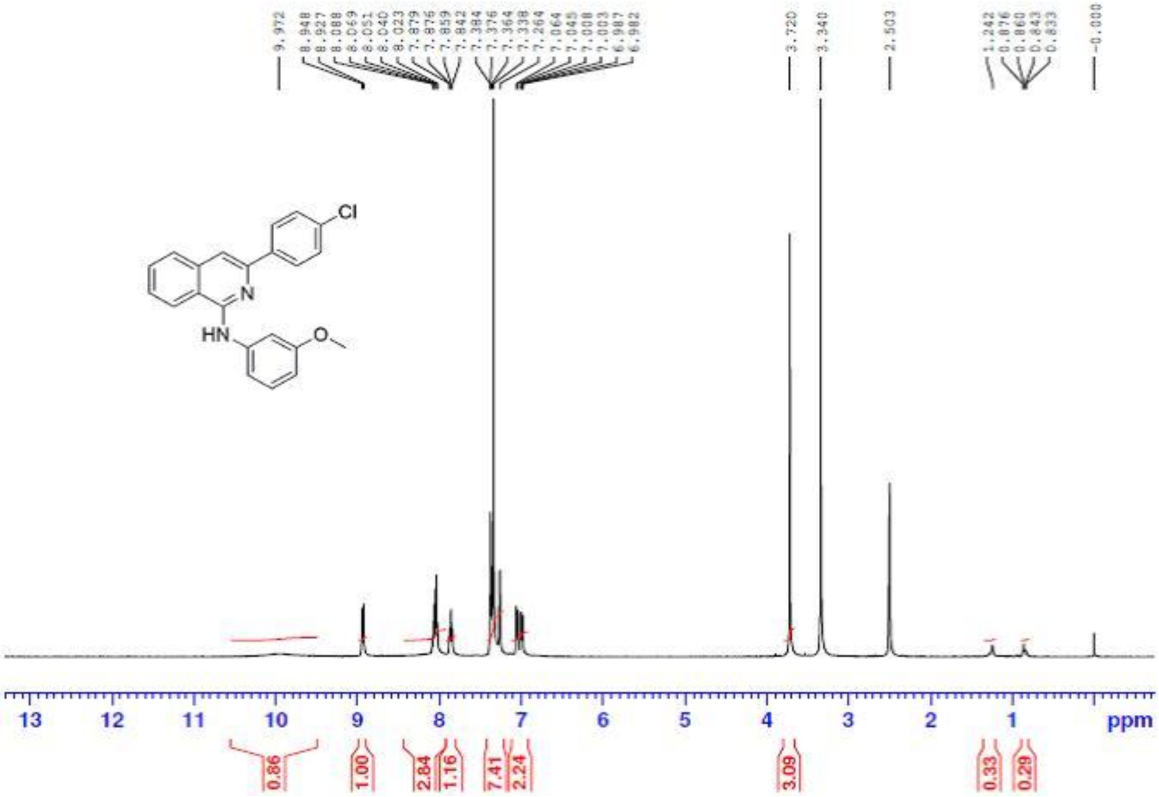

^{13}C NMR Spectrum of **5u**

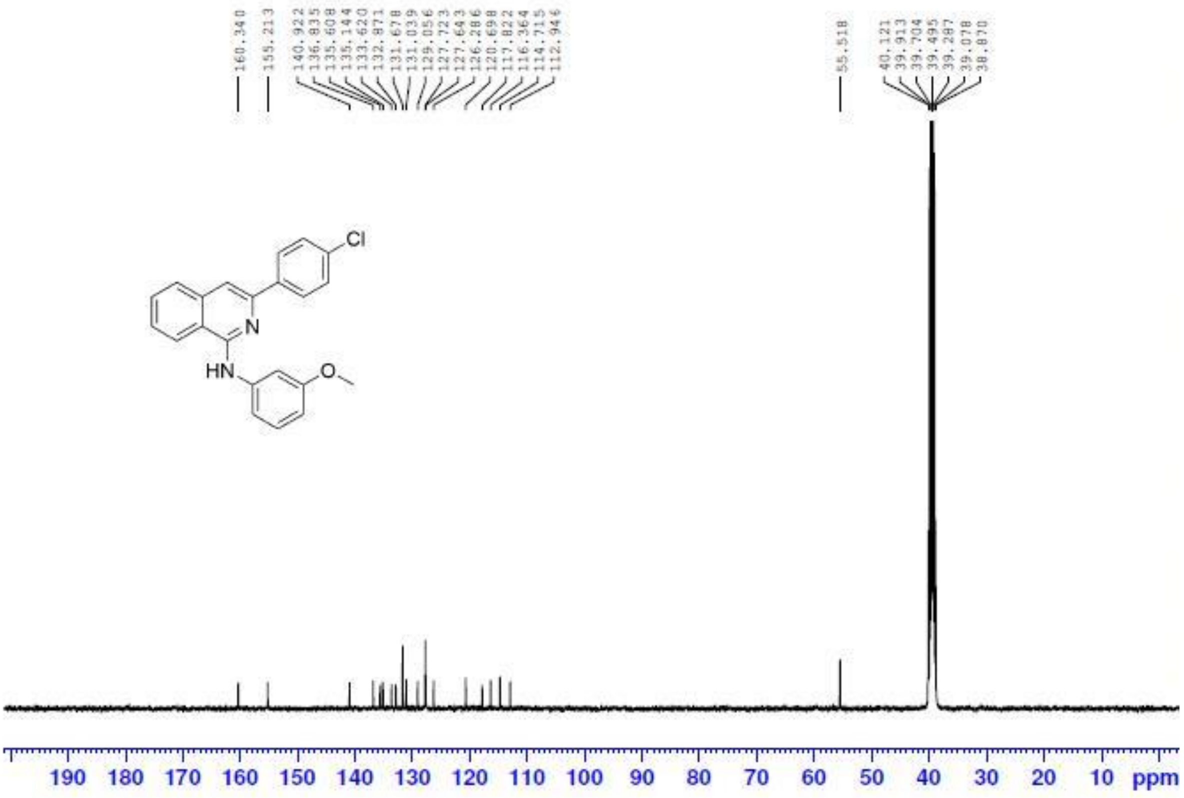

^{1}H NMR Spectrum of **5v**

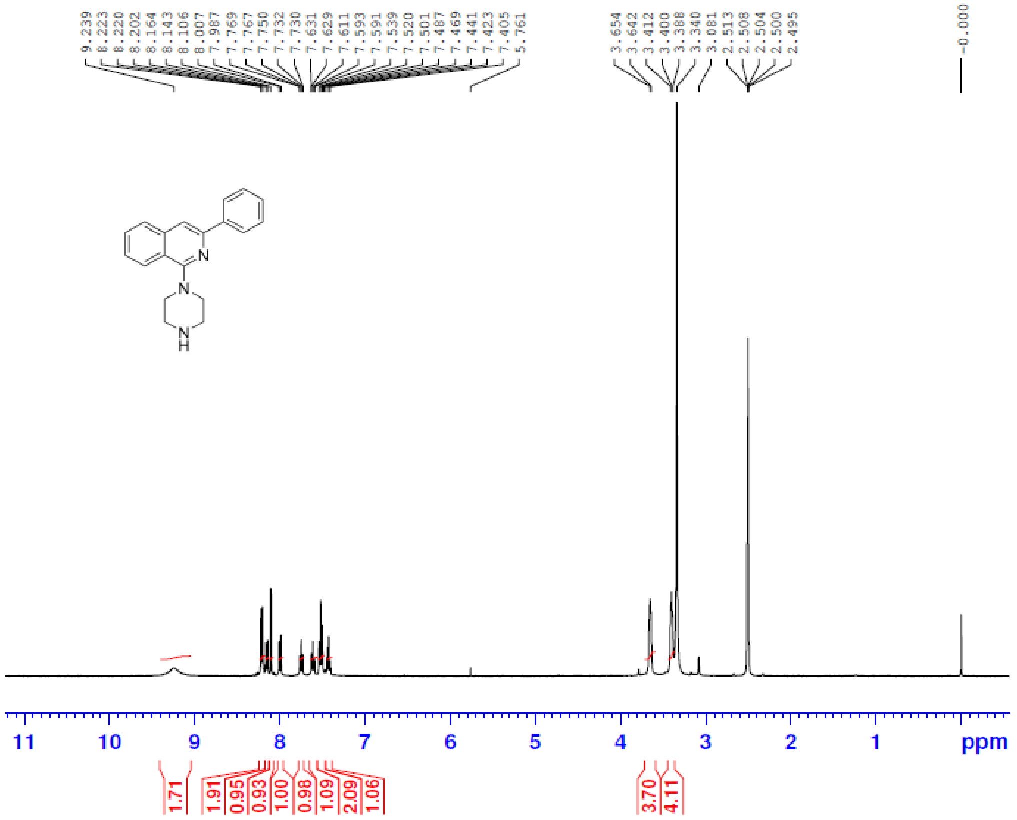

^{13}C NMR Spectrum of **5v**

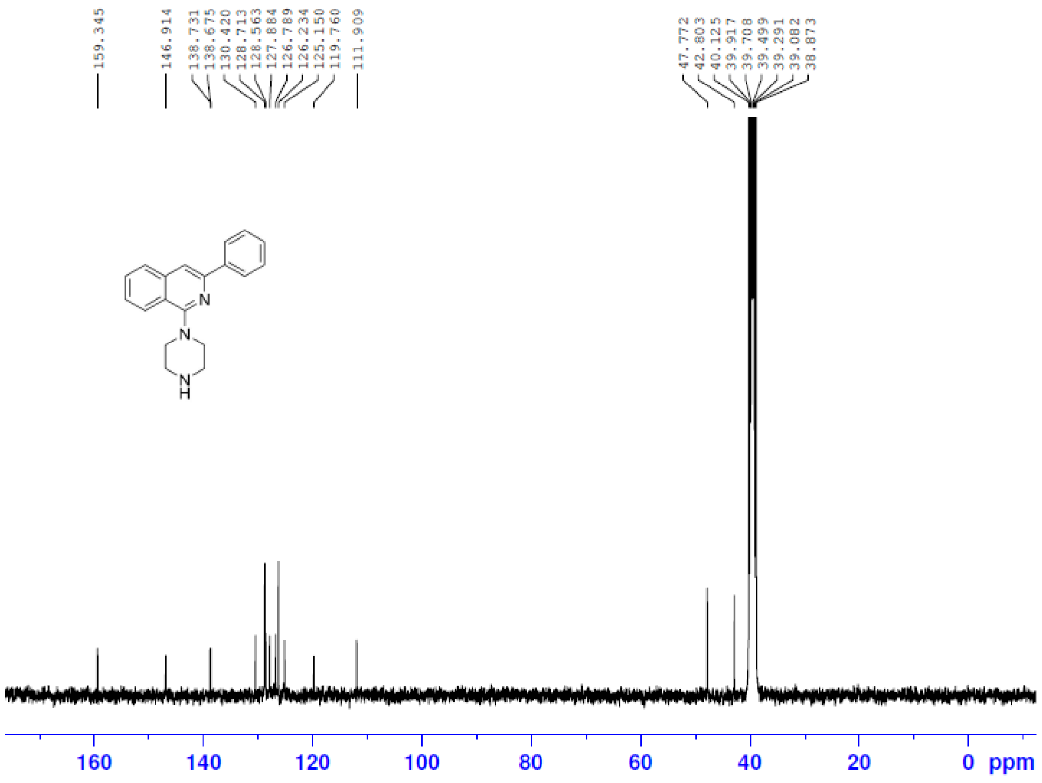

^{1}H NMR Spectrum of **5w**

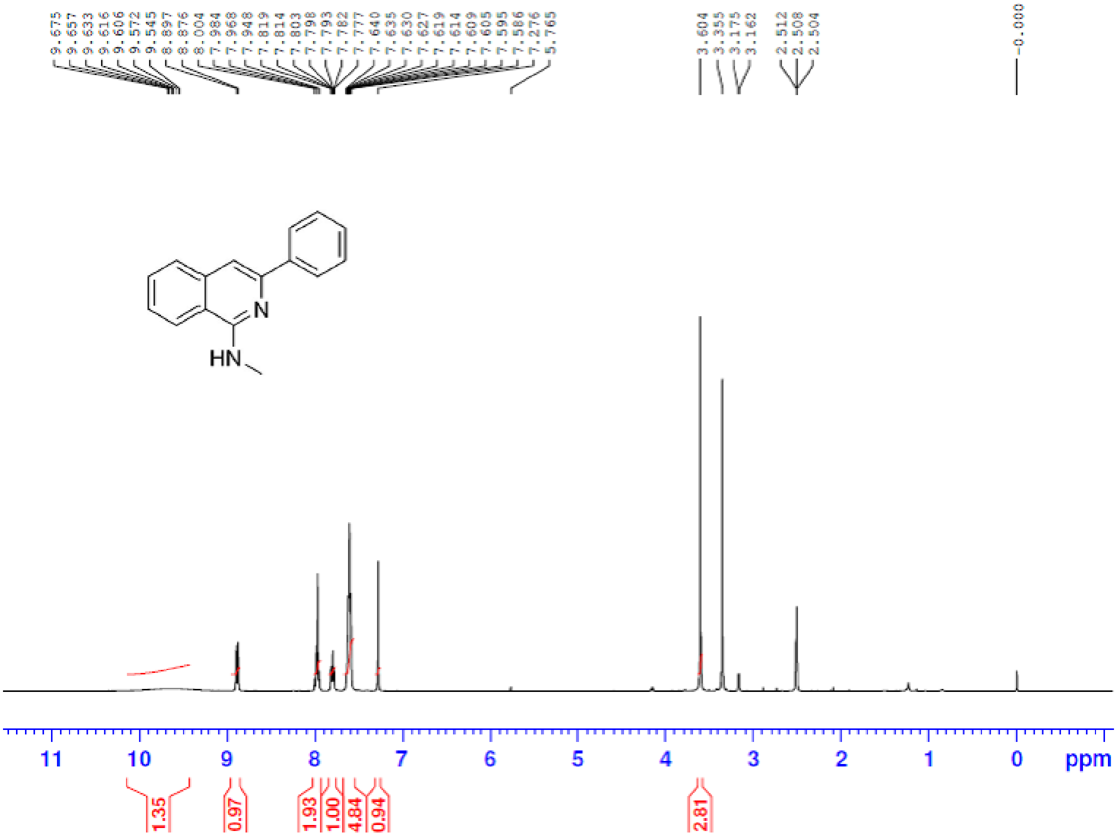

^{13}C NMR Spectrum of **5w**

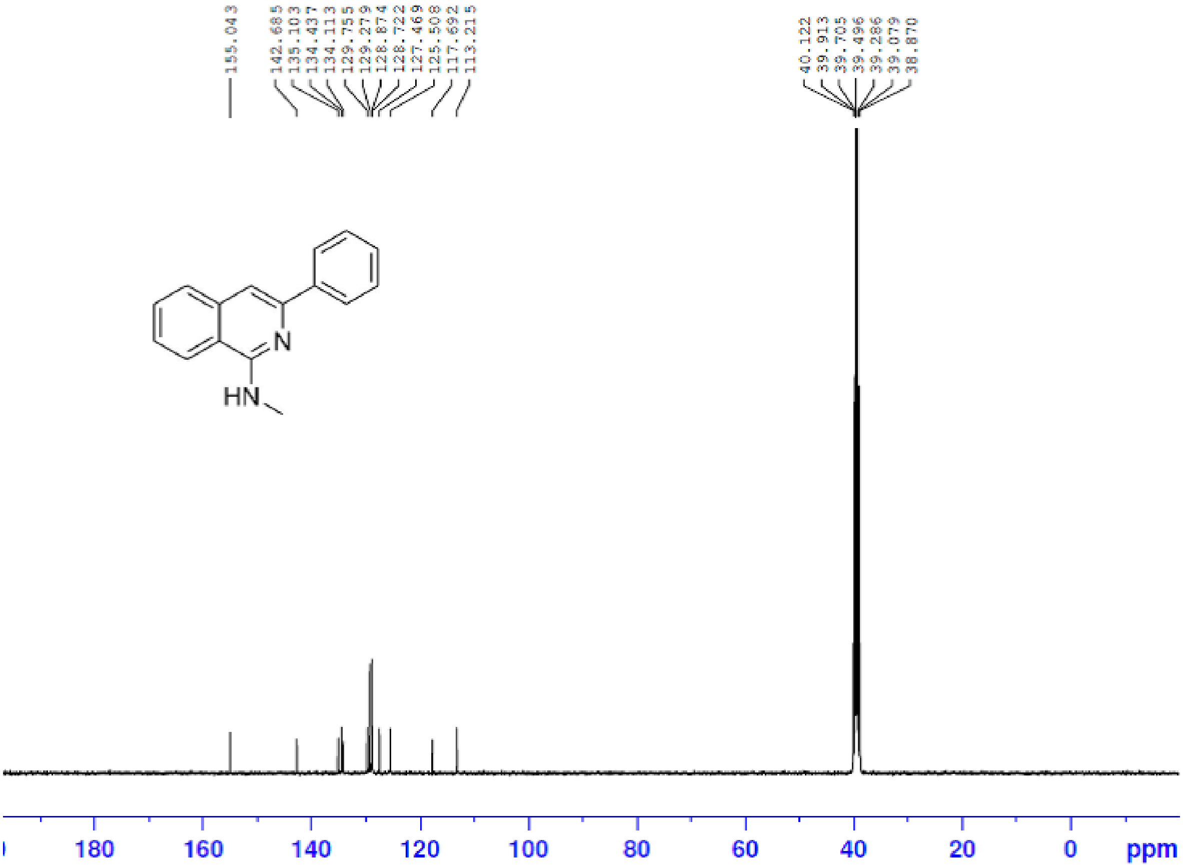

^{1}H NMR Spectrum of **5x**

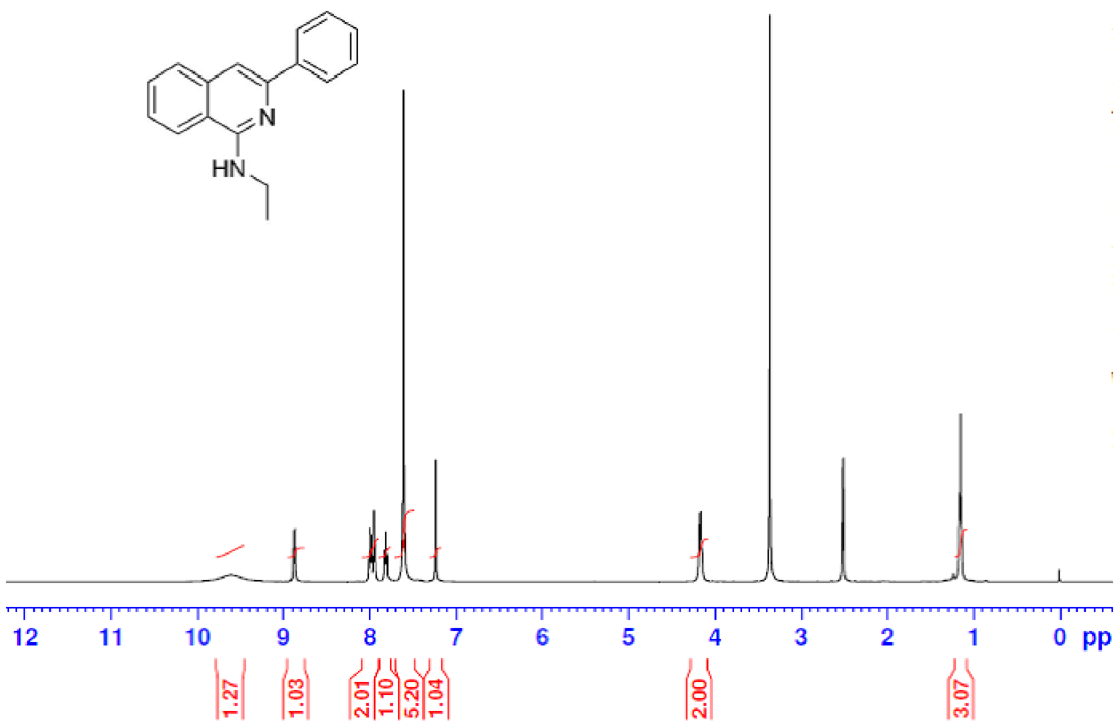

^{13}C NMR Spectrum of **5x**

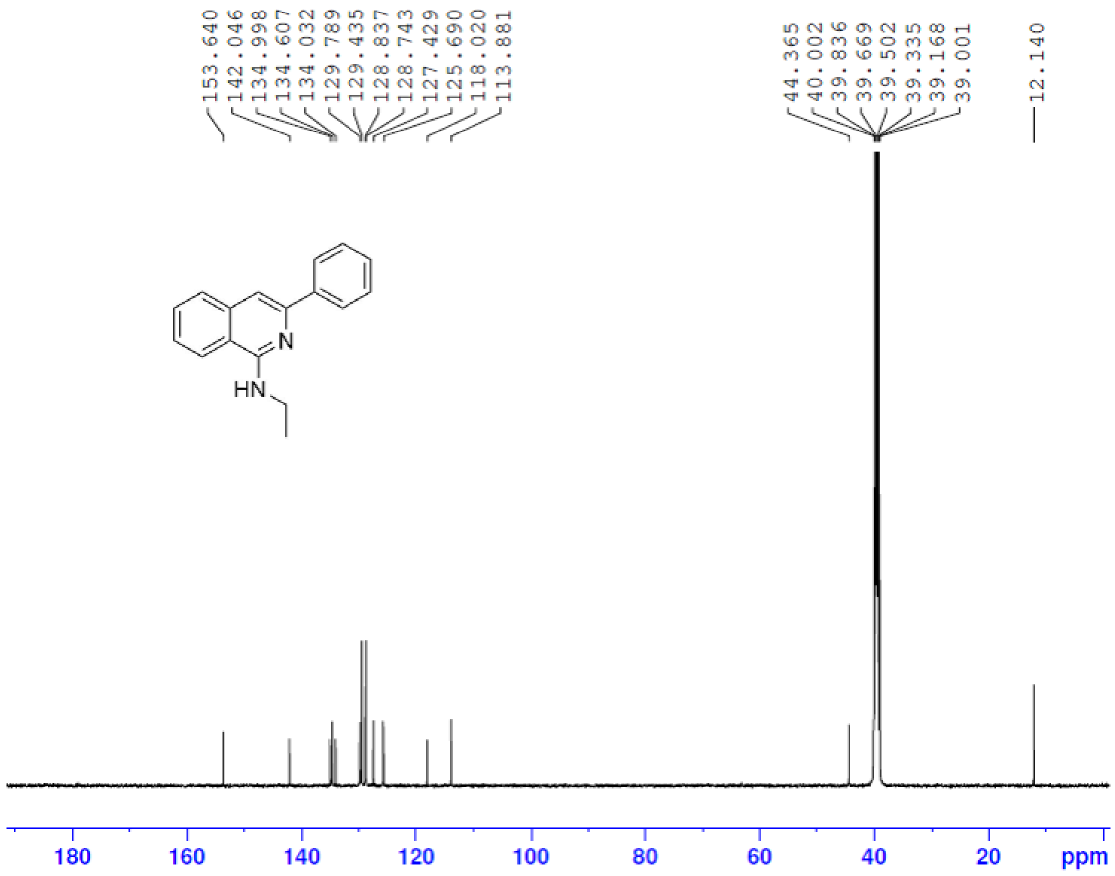

Supplement: File 1 — Experimental and analytical data. [file Beilstein_J_Org_Chem-17-2765-s001.pdf]
